# BreakTrans: uncovering the genomic architecture of gene fusions

**DOI:** 10.1186/gb-2013-14-8-r87

**Published:** 2013-08-23

**Authors:** Ken Chen, Nicholas E Navin, Yong Wang, Heather K Schmidt, John W Wallis, Beifang Niu, Xian Fan, Hao Zhao, Michael D McLellan, Katherine A Hoadley, Elaine R Mardis, Timothy J Ley, Charles M Perou, Richard K Wilson, Li Ding

**Affiliations:** 1 Department of Bioinformatics and Computational Biology, The University of Texas MD Anderson Cancer Center, 1515 Holcombe Blvd, Houston, Texas 77030, USA; 2 Department of Computer Science, Rice University, 6100 Main St, Houston, Texas 77251, USA; 3 Department of Genetics, The University of Texas MD Anderson Cancer Center, 1515 Holcombe Blvd, Houston, Texas 77030, USA; 4 The Genome Institute, Washington University School of Medicine, 4444 Forest Park Ave, St Louis, MO 63108, USA; 5 Department of Genetics, University of North Carolina School of Medicine, 120 Mason Farm Road, Chapel Hill, NC 27599, USA; 6 Department of Medicine, Washington University School of Medicine, 660 S. Euclid Ave, St Louis, MO 63110, USA

## Abstract

Producing gene fusions through genomic structural rearrangements is a major mechanism for tumor evolution. Therefore, accurately detecting gene fusions and the originating rearrangements is of great importance for personalized cancer diagnosis and targeted therapy. We present a tool, BreakTrans, that systematically maps predicted gene fusions to structural rearrangements. Thus, BreakTrans not only validates both types of predictions, but also provides mechanistic interpretations. BreakTrans effectively validates known fusions and discovers novel events in a breast cancer cell line. Applying BreakTrans to 43 breast cancer samples in The Cancer Genome Atlas identifies 90 genomically validated gene fusions. BreakTrans is available at http://bioinformatics.mdanderson.org/main/BreakTrans

## Rationale

Many cancers are driven by pathogenic expression of mRNA fusion transcripts produced by genomic structural rearrangements (GSRs) in tumor cells. Classic examples include *BCR-ABL1 *in chronic myelogenous leukaemia [1], *PML-RARa *in acute promyelocytic leukemia [2], and *TMPRSS2-ERG *in prostate cancer [3]. These fusions can arise from not only simple translocations of two distal genomic loci [4] but also complex GSRs that involve multiple distal loci [5-8]. Accurately identifying these pathogenic transcripts and the originating GSRs will have a major impact in personalized cancer diagnosis and targeted therapy [4,9].

Since 2008, next generation sequencing (NGS) technologies have been applied to identify GSR breakpoints and gene fusions. Many bioinformatics tools such as BreakDancer [10], VariationHunter [11], and CREST [12] have been developed to detect GSRs from whole genome sequencing (WGS) data. These tools predict individual genomic breakpoints by searching for clusters of abnormally mapped reads. Although generally useful, they often produce an appreciable number of false positives and false negatives introduced by insufficient coverage, short insert size, misaligned reads, GC content bias, base calling errors, and repeats [13]. Limitation in data quality and the complexity of rearrangements make it a challenging task to infer the structure of complex GSRs (or so-called genome architecture) from predicted individual breakpoints [14,15]. Meanwhile, many tools such as Tophat-fusion [16], deFuse [17], MapSplice [18], and BreakFusion [19] have been developed to detect gene fusions from whole transcriptome sequencing (WTS) data. These tools are algorithmically similar to their genomic counterparts, although they have more emphasis on mapping and ascertaining novel sequence junctions produced by mRNA-splicing and are more robust in modeling the coverage (expression). Again, these tools are associated with various types of false positives and false negatives [20] and often do not have good concordance.

When both WGS and WTS data are available, we can compare them to identify GSRs that lead to gene fusions. Because of the technical independency of these two data sources, their comparison can serve as a form of validation. In addition to improving results, mapping fusions to GSRs also elucidates the mechanistic origins of these fusions and their potential clinical values. However, such analysis is complicated by several factors. First, because of mRNA splicing, the genomic breakpoints responsible for a fusion may not be located near the fusion boundaries. Second, a fusion may be produced via multiple genomic breakpoints that join segments from distal regions of the genome. Several types of such complex GSRs have been recently revealed by WGS in various cancer types [5-7,21,22]. Third, not all GSRs produce new genes that can be transcribed. The properties (for example, location, type, and strand) of individual GSR breakpoints and the potential of producing valid open reading frames from existing genes need to be accounted for so as to produce biologically meaningful results. Fourth, current NGS data have limited power to accurately determine the genomic architectures of underlying alleles [23]. The technological limitations in resolving repeats and phase and the lack of physical coverage make it difficult to derive correct results.

To sufficiently address these challenges, systematic approaches are in demand. Recently, two bioinformatics tools, Comrad [24] and nFuse [25], were developed to address this challenge. Both tools align raw WGS and WTS reads while simultaneously corroborating fusions and GSRs. As an early effort, Comrad only maps a single fusion breakpoint to a single genomic breakpoint through the application of a set of *ad hoc *rules. As an update, nFuse maps fusion breakpoints to complex GSRs using a graph-theoretic approach. A design advantage of these tools is that they can account for ambiguous read alignment and therefore potentially minimize errors caused by misalignments. However, Comrad was only able to analyze low-path WGS data that have limited power in discovering GSR. Moreover, the self-contained design restricts them from examining hypotheses produced by other well-attested algorithms such as Tophat-fusion, MapSplice, BreakDancer and CREST.

To overcome these limitations, a modularly designed tool that focuses on mapping fusions to GSRs without re-performing breakpoint discovery may better serve the analytical demand and utilize existing resources. In this paper, we present such a bioinformatics tool, BreakTrans, that integrates the results of various fusion and GSR prediction algorithms and returns a set of genomically validated fusions with their originating alleles.

## Results

### Overview of BreakTrans

**Figure 1:**
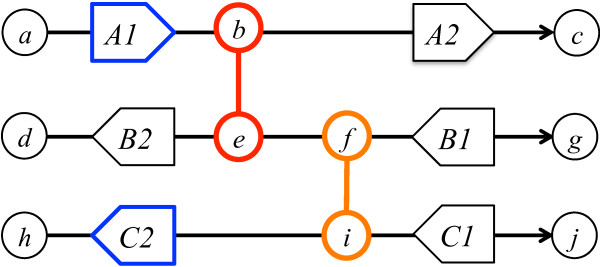
**Schematic overview of BreakTrans**. Plotted as an example are three genes, *A*, *B *and *C*, that range from genomic positions (black nodes) *a *to *c*, *d *to *g*, and *h *to *j*, respectively. Each gene contains two exons (arrow boxes) that can be transcribed from 5' to 3'. Gene *A *is on the positive (+) strand, while genes *B *and *C *are on the negative (-) strand. Two sets of putative novel genomic breakpoints are identified from alignments: *b*+

BreakTrans is designed to map gene fusions predicted by a set of fusion prediction programs, such as deFuse, MapSplice, Tophat-fusion and BreakFusion, to GSR breakpoints predicted by a set of GSR prediction algorithms, such as BreakDancer, CREST, and VariationHunter (Figure 1; Materials and methods). BreakTrans includes four major steps: 1) parse and read in GSR and fusion breakpoints produced by front-end tools; 2) construct a genomic breakpoint graph from GSR breakpoints; 3) search for genomic alleles (paths in the breakpoint graph) that support fusion hypotheses; and 4) output validated fusions and associated genomic alleles.

### Cell-line SK-BR-3

We applied BreakTrans to study the genome and transcriptome of SK-BR-3, a breast cancer cell line. We downloaded WTS data from the NCBI Sequence Read Archive [SRA:SRP003186]. We collected fusion breakpoints from three different sources. First, we analyzed the WTS data using Tophat-fusion-0.1.0 (beta) and obtained 27 fusion breakpoints. Second, we analyzed the data using BWA [26] and BreakDancer [10] with NCBI human assembly build 36 as the reference. From this analysis, we obtained 2,065 putative fusion breakpoints that contained 6 of the 10 known fusion genes in SK-BR-3 (Table 1) [27]. To further increase sensitivity, we included 28 Tophat-fusion breakpoints and 1,395 deFuse breakpoints that were previously published using the same set of WTS data [28]. This set included seven known fusion genes. Altogether, 3,498 unique fusion breakpoints were obtained (Additional file 1) that included 7 of 10 known fusion genes.

**Table 1:** Fusion genes and breakpoint paths predicted by BreakTrans-0.0.6

Number	Fusion genes	Breakpoint paths
1	*ANKHD1-EIF4EBP3>PCDH1*	5:139807117+65|5:141217466-
2	*CCDC85C>SETD3*	14:99059254-7|14:98966917-
3	*RARA>PKIA*	17:35727917+45|8:79637984+
4	*SUMF1>LRRFIP2*	3:4338455-34|3:37158400-
5	*TATDN1>GSDMB*	8:125618280-93|17:35321200-
6	*WDR67>ZNF704*	8:81882283-4|8:81882470+0|8:81916434+18|8:124171162-0|8:124158930-36|8:124161970+
		8:124171162+18|8:81916434-
7	*MTBP>SAMD12*	8:121547851+33|8:119503797+0|8:119661057+2|8:119666167-0|8:119662603-33|8:118985543+0|8:118990300+28|8:118992237-0|8:118985543-33|8:119662603+0|8:119666167+2|8:119661057-
8	*PREX1>CPNE1*	20:46795673-17|20:33925625-0|20:33923847-18|20:33679982-
9	*DHX35-ITCH*	
10	*NFS1-PREX1*	
11	*CYTH1-EIF3H*	
12	*CSE1L-ENSG00000236127*	

Eight gene fusions were predicted by BreakTrans-0.0.6, including 6 previously known fusions (1 to 6) and 2 novel ones (7 and 8). Four previously known fusions (9 to 12) were not predicted due primarily to lack of coverage. For each fusion, at least one underlying genomic allele is found, represented as a breakpoint path that consists of a serial of breakpoint strings (Materials and methods). '>' represents the predicted (5' to 3') order of a gene fusion.

To obtain a set of genomic breakpoints, we generated 80-fold 100 bp paired-end WGS reads (Illumina) from genomic DNA [SRA:SRP028176]. We mapped the WGS reads against build 36 reference using BWA and performed BreakDancer and CREST [29] analysis. BreakDancer and CREST predicted 23,567 (>1,000 bp) and 18,048 genomic breakpoints, respectively. Altogether, 41,615 unique genomic breakpoints were obtained (Additional file 2).

We ran BreakTrans-0.0.6 on these two sets of fusion and genomic breakpoints and obtained a set of 40 redundant fusion breakpoints that are supported by genomic alleles (Additional file 3). These fusion breakpoints are redundant (in location) due to our inclusion of multiple sources at variable nucleotide resolutions. Altogether, these 40 breakpoints nominated 8 unique fusion genes (Table 1), including 6 of the 10 known fusion genes and 2 novel ones.

Of the four known fusion genes that we missed, *DHX35-ITCH *and *NFS1-PREX1 *were likely due to insufficient coverage of the transcriptome, as indicated by a previous study [28]. *CYTH1-EIF3H *was due to insufficient coverage of the genome: neither BreakDancer nor CREST detected any genomic rearrangements that can be associated with this fusion. Although the WGS data we used have great sequence coverage (80-fold), their physical coverage is quite limited: the average insert size is only 211 bp with a read length of 100 bp. *CSE1L-ENSG00000236127 *has become obsolete because of the exclusion of *ENSG00000236127 *from the Ensembl database, as previously explained [28]. BreakTrans was able to validate all known fusions with sufficient coverage from this dataset, indicating its high sensitivity.

For comparison purposes, we ran nFuse-0.1.4 on the same WTS and WGS datasets using default parameters. Among the 1,994 predicted fusion breakpoints (Additional file 4), only 2 of the known fusion genes (*ANKHD1-PCDH1 *and *SUMF1-LRRFIP2*) were identified.

**Figure 2:**
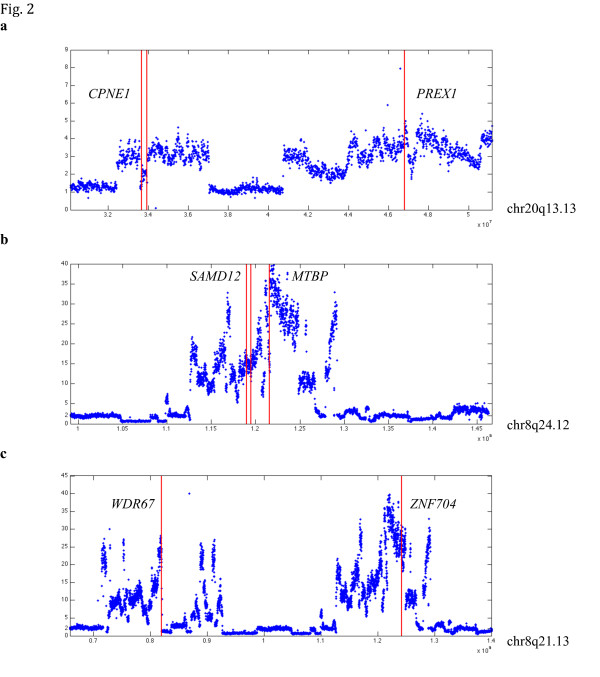
**Copy number profile in the SK-BR-3 genome**. Plotted are three gene fusions predicted by BreakTrans: **(a) ***PREX1*>*CPNE1*, **(b) ***MTBP*>*SAMD12*, **(c) ***WDR67*>*ZNF704*. The x-axis represents genomic positions and the y-axis represents absolute copy number in non-overlapping 10 kb windows. The vertical red lines mark the locations of the GSR breakpoints that led to these fusions.

The two novel fusion genes *PREX1-CPNE1 *and *MTBP-SAMD12 *detected by BreakTrans were both nominated by deFuse and are both likely to be valid. *PREX1*, *CPNE1*, and *SAMD12 *have, respectively, fused with other genes in breast cancer cell-lines: *NFS1-PREX1 *in SK-BR-3, *CPNE1-PI3 *in BT-474 [27], and *PHF20L1-SAMD12 *in HCC1954 [25,30]. The *PREX1-CPNE1 *fusion occurs precisely at the known exon boundaries (Figure S1 in Additional file 5). Genomic regions containing these genes underwent substantial copy number alterations (CNAs) (Figure 2), which have been shown to co-occur with gene fusions [27]. Furthermore, both *PREX1 *and *MTBP *have been previously implicated in breast cancer progression [31-33].

**Figure 3:**
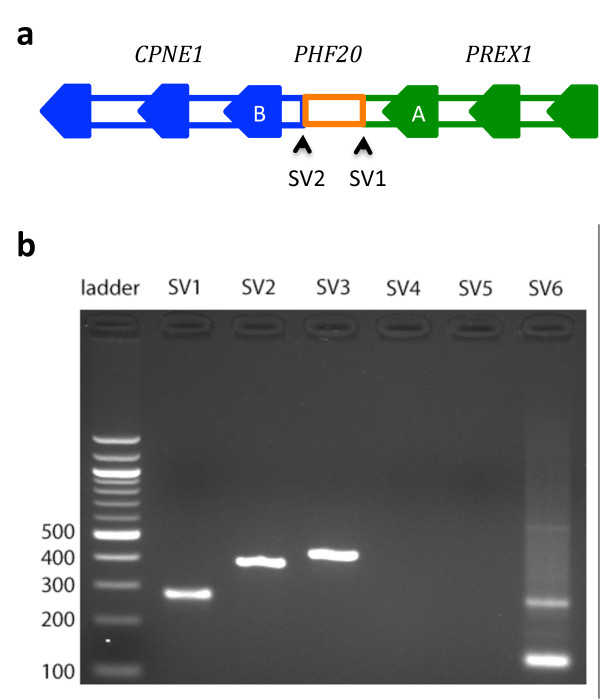
**Breakpoints of novel fusions in SK-BR-3**. **(a) **The novel tumor allele supporting *PREX1*>*CPNE1 *consists of the first three exons of *PREX1 *(green), an intronic segment of *PHF20 *(orange) and the last three exons of *CPNE1 *(blue). The RNA *PREX1*>*CPNE1 *breakpoint between exons *A *and *B *was nominated by deFuse, while the genomic breakpoints SV1 and SV2 were detected by CREST. **(b) **Six genomic breakpoints from three gene fusions were selected for PCR validation (Additional file 8). SV1 and SV2 are from *PREX1*>*CPNE1*, SV3 and SV4 from *MTBP-SAMD12*, and SV5 and SV6 from *WDR67-ZNF704*. Clean PCR bands were observed at SV1, SV2, SV3 and SV6.

These eight fusion genes were supported by nine unique alleles, as shown by the breakpoint paths in Table 1. Six of the nine alleles contain one unique genomic breakpoint, representing the simplest way of generating fusion. The allele that encodes *PREX1-CPNE1 *contains two breakpoints, which connect DNA segments from three different genes on chromosome 20. Included are the first three exons of *PREX1*, an intronic segment of *PHF20*, and the last three exons of *CPNE1 *(Figure 3a). These breakpoints are highly supported by WGS data: 17 soft-clipped reads were found at the *PREX1-PHF20 *breakpoint and 18 at the *PHF20-CPNE1 *breakpoint (Figures S2 to S5 in Additional file 5). They also overlap precisely with CNA boundaries (Figure 2a). The *MTBP-SAMD12 *allele contains five unique breakpoints, all located in a single complex amplicon on chromosome 8. At least three breakpoints clearly associate CNA boundaries (Figure 2b) with clusters of soft-clipped reads identified (Figures S6 to S9 in Additional file 5). The breakpoint path indicates an inverted duplication, a type of genomic rearrangement that has been commonly observed in breast cancer cell lines [21]. The *WDR67-ZNF704 *fusion was supported by two different alleles, containing three breakpoints and one breakpoint, respectively. These breakpoints also associate the boundaries of two distal amplicons on chromosome 8 (Figure 2c) with soft-clipped reads identified (Figures S10 and S11 in Additional file 5).

To validate these novel fusion breakpoints, we generated two independent paired-end RNA-seq datasets (SKBR3-1 and SKBR3-2; 76 bp read length) using the SK-BR-3 lines in our lab [SRA:SRP028176]. Both *PREX1-CPNE1 *and *MTBP-SAMD12 *were rediscovered at identical breakpoints using Tophat-fusion and BreakFusion, together with nine of the previous known fusions (Additional files 6 and 7). Note that both novel fusions were originally nominated using publicly available RNA-seq data (50 bp read length) [SRA:SRP003186] by deFuse [28], which employs alignment and fusion-calling algorithms very different from either Tophat-fusion or BreakFusion. Such independence in the data and in the analytical approaches supports both novel fusions predicted by BreakTrans as being real biological events. Interestingly, we also re-identified the genomic *PREX1-PHF20 *breakpoint in one of the RNA-seq datasets (SKBR3-2) (Additional file 7), which validated the existence of this breakpoint in the pre-mRNAs.

We further validated a set of associated genomic breakpoints using PCR (Additional file 8). If these genomic breakpoints were real, we should be able to observe PCR bands at expected DNA amplicon sizes. Out of the six breakpoints that we were able to design primers for, four amplicons had very clean bands (Figure 3b), which included both of the two breakpoints for *PREX1-CPNE1*, one for *MTBP-SAMD12 *and one for *WDR67-ZNF704*. Interestingly, two different PCR bands were observed at one of the *WDR67-ZNF704 *breakpoints (Figure 3b), consistent with our prediction that the *WDR67-ZNF704 *fusion is associated with two different genomic alleles.

### BreakTrans analysis of The Cancer Genome Atlas breast cancer WGS and WTS datasets

We applied BreakTrans to 43 breast cancer samples with both WTS and WGS data from The Cancer Genome Atlas (TCGA; 12/30/2012) [34]. mRNA fusion breakpoints were nominated by BreakDancer by identifying clusters of read pairs that span different genes from the WTS BAM files produced by MapSplice [18]. The genomic breakpoints were detected by two programs, BreakDancer and SquareDancer (K Chen *et al*., unpublished), which examine discordant read pairs and soft-clipped reads, respectively. Together, we obtained a set of 156,955 redundant mRNA fusion breakpoints (an average of 3,650 per sample) and another set of 305,743 genomic breakpoints (an average of 6,794 per sample). We applied BreakTrans on these sets of breakpoints in conjunction with gene models specified in TCGA Genome Annotation Format (GAF) version 2.1, provided by the University of California Santa Cruz.

BreakTrans identified 177 redundant fusion breakpoints with convincing genomic evidence, which corresponded to 90 unique sample gene pairs (Additional file 9).

None of the fusions was found to be recurrent with identical gene pairs, suggesting a high level of heterogeneity in breast cancer as consistently demonstrated by previous studies [35]. However, we found a set of genes that recurrently partnered with others: *CBX3*, *C15orf57*, *BCAS3*, *RARA*, *USP15*, *PTPRN2*, *USP32*, *FBXL20*, *SNX27*, *WIPF2*, *NF1 *and *RAD51C*. Notably, the *USP *family members (*USP13*, *USP15*, and *USP32*) were frequently involved (in five fusions). Several fusions involved a kinase at the 3' end and are potentially viable therapeutic targets: *USP13*-*PIK3CA*, *GPR160-PRKCI*, and *FBXL20-TLK2*. Among the 43 samples, 33 were found to have more than 2 gene fusions, with one (A09I) containing 10 fusion genes. Sample A09I also demonstrated extensive genomic instability with many CNAs, including focal amplification of over 60-fold (Figure S12 in Additional file 5).

Most of the 90 fusions (83.3%) involved one genomic breakpoint and 2 distal loci. The rest involved multiple genomic breakpoints. For example, the *NF1-NLE1 *fusion in A09I involved two breakpoints and three genes (*NF1*, *CA0 *and *NLE1*; Figures S13 to S18 in Additional file 5), the *PPP1R1B*-*PIPOX *fusion in A0D1 involved three breakpoints and three genes (*PPP1R1B*, *NOS2 *and *PIPOX*; Figures S19 to S27 in Additional file 5), and the *PPP3R1*-*TTC27 *fusion in A0YG involved three breakpoints and four genes (*PPP3R1*, *USP34*, *LTBP1 *and *TTC27*; Figures S28 to S37 in Additional file 5). Both *NF1-NLE1 *and *PPP1R1B*-*PIPOX *occurred on the chr17q hotspot with GSR boundaries precisely overlapping CNA boundaries (Figures S12, S14, S20 and S21 in Additional file 5). *PPP3R1*-*TTC27 *occurred on chromosome 2 and was evidently associated with chromothripsis (Figures S29 to S31 in Additional file 5) [6].

To prove the validity of BreakTrans predictions, we performed PCR validation on 20 genomic breakpoints (Additional file 10), including 9 that were associated with the above 3 multi-breakpoint fusions and 11 that we randomly selected from 9 samples. Out of these 20 breakpoints, 15 were validated as somatic, 1 as germline, and 4 as wild type (Additional file 11 and Figures S38 to S41 in Additional file 5). Further capillary sequencing of the PCR products confirmed the existence of one more breakpoint (Figure S42 in Additional file 5). Among the validated breakpoints were both of the two breakpoints underlying *NF1-NLE1*, all of the three breakpoints underlying *PPP3R1-TTC27*, and two of the three breakpoints underlying *PPP1R1B-PIPOX*.

## Discussion

In this work, we present a novel bioinformatics approach, BreakTrans, that systematically maps detected gene fusions to novel genomic alleles produced by GSRs, thereby validating both sets of hypotheses and providing mechanistic interpretation to validated fusions. Our analysis and experimental validation indicated very high specificity of BreakTrans. The true specificity is likely higher than our estimation (60 to 80%), given the difficulties in performing PCR validation in repetitive regions.

Our results indicated that BreakTrans could achieve higher sensitivity through integration of multiple predictors without demonstrably increasing false positive rate. This is particularly important for current practice as individual predictors tend to be conservatively configured to achieve individually low false positive rates at the cost of increasing false negative rates. This phenomenon is particularly evident in our SK-BR-3 analysis, where we observed a large proportion of calls unique to a predictor. Conventional strategies that summarize results based on majority rules have been shown to be helpful in reducing false positives [36]. However, the further loss in sensitivity was usually not characterized. Applying BreakTrans to integrate multiple call sets is clearly a different and more effective strategy, as it integrates additional data. Indeed, the two novel fusions in the SK-BR-3 set were only nominated by deFuse and would have been eliminated if a simple consensus approach were taken. Our modular design allowed users to utilize their favorite predictors and include hypotheses from any source (for example, literature). This feature relieves users from trying to determine the best predictors and post-processing strategies for their data, a non-trivial task.

Another contribution of our work is that we proposed a convention (breakpoint string) to represent individual breakpoints and breakpoint graphs, as well as simple or complex alleles that encompass one or more breakpoints. This allows the reporting and communicating of large numbers of complex hypotheses in a concise and accurate way, an important requirement for large-scale sequencing and clinical sequencing efforts [37]. It also relieves researchers from manually piecing together alleles from individual breakpoints, a complex and error-prone task.

Our current version does not contain a scoring system to characterize the confidence of output fusions and alleles. This is mainly due to the complexity in integrating heterogeneous predictions from different sources, which are associated with heterogeneous scoring systems and precision. With this version in place, we are actively working on approaches to re-score breakpoints and alleles using a genotype-likelihood framework [13,36], which will be implemented in a future version of BreakTrans.

Although BreakTrans can effectively eliminate false breakpoints by leveraging the independence of WGS and WTS data and the existing knowledge of the human transcriptome, the quality of the results is clearly dependent on the quality of the input. If a large number of false breakpoints were included and true breakpoints excluded, any approach will have difficulty deriving correct answers. Improving breakpoint accuracy itself is a non-trivial task given the complexity of the cancer genome and the limitation of NGS [13]. Therefore, it is important to apply modular design that allows problems and efforts to be distributed. BreakTrans makes it possible to separate the problem of breakpoint integration from that of breakpoint identification. Further improvement in either area will synergistically improve the final results.

Similar to other programs, BreakTrans requires sufficient coverage on both genomic and transcriptomic breakpoints to validate an event. Failure to validate an event does not necessarily negate its existence. This is a fundamental problem in analyzing heterogeneous tumor samples that often contain multiple clones of tumor cells [38] - that is, subclonal breakpoints may not receive sufficient coverage from standard bulk sample sequencing. However, as NGS continues evolving and its cost continues reducing, it becomes increasingly feasible to obtain deep coverage on both the genome and the transcriptome of subclonal cell populations [38] or even single cells [39].

## Summary

We have developed a bioinformatics tool, BreakTrans, that systematically maps gene fusions to GSRs, an application that is important for molecular diagnosis and targeted therapy. Instead of re-performing breakpoint discovery, BreakTrans integrates breakpoint hypotheses from various sources using a novel breakpoint graphic approach. Our examination using the WGS and WTS data from breast cancer cell-line SK-BR-3 indicates that BreakTrans has achieved higher sensitivity and specificity than existing approaches. Applying BreakTrans to the 43 breast cancer samples in TCGA, we have identified a set of 'genomically validated' gene fusions that are promising for further functional study. As sequencing coverage continues to increase, we anticipate wide application of BreakTrans in both research and clinical settings.

## Materials and methods

### Representing genomic breakpoints

**Figure 4:**
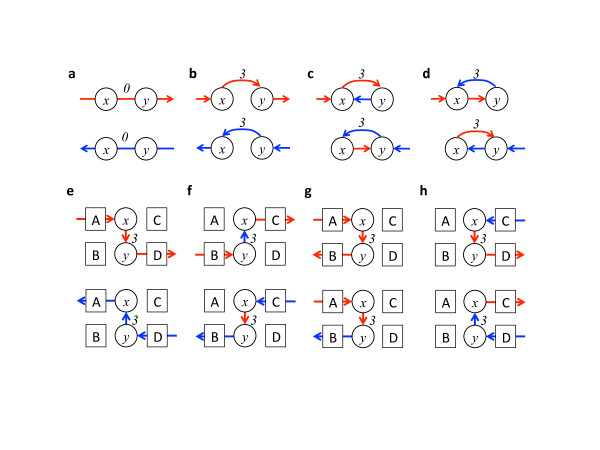
**Definition of breakpoints and breakpoint strings**. For intra-chromosome rearrangements, four types of breakpoints - **(a) **null, **(b) **jump, **(c) **inverse and **(d) **repeat between genomic positions *x *and *y *- can be created that involve DNA on either the positive (red arrow) or the negative (blue arrow) strands. Edges are labeled with a number (for example, 3) representing confidence scores (for example, number of supporting reads) for the predicted adjacency. Edges without a number or with the number '0' represent reference adjacency (null breakpoints). For inter-chromosomal rearrangements, four possible novel alleles - **(e) **A-D, **(f) **B-C, **(g) **A-B and **(h) **C-D - can be created by joining four breakends (A, B, C, D) from two wild-type alleles (A-C, B-D) through genomic breakpoints *x *and *y*. Each allele can be represented in two different orientations involving combinations of either the positive (red arrow) or the negative (blue arrow) strands. **(i) **Breakpoint strings corresponding to the above configurations are listed, where '+' represents the positive strand and '-' the negative strand. The syntax of breakpoint strings is further explained in the Materials and methods.

Existing GSR detection programs such as BreakDancer and CREST predict individual breakpoints from clusters of abnormally aligned paired-end reads or soft-clipped reads. Each breakpoint represents a joining of two non-adjacent DNA segments (break-ends) that are adjacent in the reference genome. These breakpoints can be created by either simple genomic rearrangements, such as deletion, insertion, and duplication, or complex genome rearrangements, such as chromothripsis or close-chain translocation that creates multiple breakpoints [5-7,25]. The resulting relationship between the two break-ends in the subject genome is called novel adjacency, as it does not exist in the reference genome. Such a breakpoint can be represented using a graphic representation known as a breakpoint graph [40]. Here, we define a breakpoint representation in the same vein, although it is more compact to use in our context. We define a 'breakpoint string' to specify exactly how two DNA break-ends are joined together at the breakpoint (Figure 4). A breakpoint string consists of two break-ends: an in-end and an out-end. The in-end represents the end point of a DNA segment before entering the breakpoint. The out-end represents the start point of another DNA segment after exiting the breakpoint. The ends are directional (double stranded). We use '+' to represent the positive strand and '-' to represent the negative strand. Each break-end is uniquely specified by a reference genomic coordinate *x *(consisting of a chromosome and a position) and a direction. We use a score *f *to quantify the confidence of the existence of the breakpoint. Popularly used scores include the number of reads or read pairs spanning the breakpoint or a genotype likelihood [41]. For notational convenience, we use a vertical bar '|' to represent the connection between an in-end and an out-end.

The definitions above allow us to specify breakpoints produced by various types of genomic structural rearrangements in a consistent and concise format (for example, *x+f|y+*). We further define four types of intra-chromosomal rearrangement breakpoints: 'null', 'jump', 'inverse', and 'repeat' (Figure 4a-d). A 'null' breakpoint represents no breakpoint between *x *and *y *and the sequence between them is identical to the reference genome. We use a special score *f *= 0 to denote such a 'null' breakpoint. A 'jump' breakpoint joins together two non-adjacent segments on the same strand and skips the sequence between *x *and *y*. A breakpoint resulting from a deletion can be represented as a 'jump'. An 'inverse' breakpoint joins together two non-adjacent segments in opposite strand/orientation; it can represent breakpoints produced by inversions or inverted duplication. Finally, a 'repeat' breakpoint connects *x *back to an upstream position *y *on the same strand; it can represent breakpoints produced by tandem duplication. Similarly, we can use a breakpoint string to represent an inter-chromosome breakpoint, resulting from four different ways of joining the break-ends (Figure 4e-h). Taken together, breakpoint strings defined by our rules can encode most, if not all, rearrangement breakpoints. Similar to DNA, a breakpoint string can be reverse-complemented by swapping the positions of *x *and *y *and flipping the orientations - that is, x+3|y+ is identical to y-3|x-albeit on the opposite strand. This feature allows us to encode breakpoints as undirected edges while enabling strand-aware search.

### Constructing the breakpoint graph

All of the existing NGS structural variant detection software output breakpoints individually, representing aberrant adjacencies in the subject genome. We can connect these breakpoints together to form a breakpoint graph, in which a node represents a genomic position that either terminates or leads a break-end, and an edge represents a breakpoint. The edges are undirected and are specific to various types of breakpoints, as specified by the breakpoint strings. In a polyploid genome, multiple alleles (chromosomes) are present. A node can thereby have multiple edges, each representing a different allele. Where no aberrancy is detected, the subject genome is assumed to have the same allele as the reference genome. To represent a complete genomic architecture, edges with null breakpoints are added to represent the reference alleles that connect the breakpoints. Note that our representation is different from those used by nFuse, in which a node represents a break-end on a specified strand. In our case, a node only represents a position; whether it leads or terminates a break-end on a specific strand depends on specifications on the connecting edges.

With a breakpoint graph constructed, the task of decoding chromosomal architecture involves identifying paths that start at the beginning and extend to the end of the chromosome. It is clearly a computationally challenging problem to identify correct paths in a graph that contains lots of nodes and edges.

### Transcriptome-guided search

To achieve accuracy and efficiency, it is desirable to simplify the graph. Rather than trying to decode the complete genome (global optimization), we can focus on expressed regions (local optimization). We ignore read-through events, which are out of our current scope, by disconnecting the reference allele (null edges) between the end nodes of neighboring genes. We can always restore these connections if read-though events are of interest.

Similar to a genomic breakpoint, a fusion (transcriptome) breakpoint predicted from mapping RNA-seq reads to the reference genome can be specified by two genomic positions *x *and *y *that are located in two different genes. To determine the underlying allele from which the fusion is transcribed, we first identify the nearest genomic breakpoints (*x*_0_, *y*_0_) downstream of *x *and *y *in the breakpoint graph. We then start at *x*_0 _and perform a recursive breadth-first search:

*p*(*x*_0_) = *x*_0_+*p*(*n*(*x*_0_))

where the function *p*(*x*) denotes the alleles starting at *x*, *n*(*x*) denotes the set of nodes that connect to *x *and + represents path extension. A path terminates if it hits either node *y*_0 _or the end of a gene. This search algorithm returns all genomic alleles (or breakpoint paths) in the breakpoint graph that support a fusion hypothesis.

### Data accessibility

BreakTrans code is available to download at [42]. The SK-BR-3 WGS and RNA-seq data are available in the NCBI SRA [SRA:SRP028176]. The TCGA breast cancer WGS and RNA-seq data can be obtained through dbGAP (accession number phs000178.v7.p6).

## Abbreviations

bp: base pair; CNA: copy number alteration; GSR: genomic structural rearrangement; NGS: next generation sequencing; PCR: polymerase chain reaction; SRA: Sequence Read Archive; TCGA: The Cancer Genome Atlas; WGS: whole genome sequencing; WTS: whole transcriptome sequencing.

## Competing interests

The authors declare that they have no competing interests.

## Authors' contributions

KC and LD conceived the study; KC designed and implemented the code; KC, JW, BN, MM, HZ and XF performed the analysis; NN provided SK-BR-3 WGS and RNA-seq data; YW and HS performed PCR validation, KC, NN and LD wrote the manuscript; LD, CP, KH, TL, RW and EM provided oversight and coordination of TCGA analysis. All authors read, revised and approved the final manuscript.

## Supplementary Material

Additional file 1 **A list of 3,498 candidate gene fusion breakpoints in SK-BR-3, used as input to BreakTrans**. Click here for file

Additional file 2 **A list of 41,615 candidate genomic breakpoints in SK-BR-3, used as input to BreakTrans**. Click here for file

Additional file 3 **A list of 40 genomically validated fusion breakpoints output by BreakTrans-0.0.6**. Click here for file

Additional file 4 **A list of 1,994 candidate gene fusions predicted by nFuse-0.1.4**. Click here for file

Additional file 5 **Figures S1 to S42**. Figure S1: *PREX1-CPNE1 *fusion detected from SK-BR-3 WTS data. Figures S2 to S5: integrative genomics views (IGVs) of read alignments at the two GSR breakpoints (four break-ends) underlying *PREX1-CPNE1*. Figures S6 to S9: IGVs of GSR breakpoints underlying *MTBP-SAMD12*. Figures S10 and S11: IGVs of GSR breakpoints underlying *WDR67-ZNF704*. Figure S12: whole genome somatic copy number alteration (log2) of A09I. Figure S13: the *NF1-NLE1 *fusions detected in A09I. Figure S14: somatic copy number alteration on chromosome 17 of A09I with red vertical lines marking the GSR breakpoints that support the *NF1-NLE1 *fusion. Figures S15 to S18: IGV of the two GSR breakpoints (four break-ends) that underlie *NF1-NLE1*. Figure S19: the *PPP1R1B-PIPOX *fusion detected in A0D1. Figure S20: whole-genome somatic copy number alteration (log2) of A0D1. Figure S21: somatic copy number alteration on chromosome 17 of A0D1 with red vertical lines marking the GSR breakpoints that support the *PPP1R1B-PIPOX *fusion. Figures S22 to S27: IGVs of the three GSR breakpoints (6 break-ends) that underlie *PPP1R1B-PIPOX*. Figure S28: the *PPP3R1-TTC27 *fusion detected in A0YG. Figure S29: whole-genome somatic copy number alteration (log2) of A0YG. Figure S30: somatic copy number alteration on chromosome 2 of A0YG with red vertical lines marking the GSR breakpoints that support the *PPP3R1-TTC27 *fusion. Figure S31: zoomed-in view of Figure S29 at the chromosome 2 chromothripsis that harbors the fusion. Figures S32 to S37: IGVs of the three GSR breakpoints (6 break-ends) that underlie *PPP3R1-TTC27*. Figures S38 to S41: PCR validation of TCGA BRCA genomic breakpoints. Figure S42: capillary sequencing trace of a PCR product that was not visible in the gel. Click here for file

Additional file 6 **A list of 124 fusion breakpoints (42 gene pairs) produced by Tophat-fusion from the validation RNA-seq data**. Click here for file

Additional file 7 **A list of 115 fusion breakpoints (62 gene pairs) produced by BreakFusion-1.0.1 from the validation RNA-seq data**. Click here for file

Additional file 8 **Breakpoints and PCR primers for SK-BR-3 validation**. Click here for file

Additional file 9 **A list of 177 gene fusions and corresponding genomic breakpoints produced by BreakTrans from 43 TCGA breast cancer samples**. Click here for file

Additional file 10 **Experimental design for validating 20 TCGA GSR breakpoints**. Click here for file

Additional file 11 **PCR validation of 20 TCGA GSR breakpoints in 9 TCGA samples**. Click here for file

## References

[B1] Rowley JD Letter: A new consistent chromosomal abnormality in chronic myelogenous leukaemia identified by quinacrine fluorescence and Giemsa staining. Nature 1973 14 290 293 10.1038/243290a0 4126434

[B2] Huang ME Ye YC Chen SR Chai JR Lu JX Zhoa L Gu LJ Wang ZY Use of all-trans retinoic acid in the treatment of acute promyelocytic leukemia. Blood 1988 14 567 572 3165295

[B3] Tomlins S Mehra R Rhodes D Cao X Wang L Dhanasekaran S Kalyana-Sundaram S Wei J Rubin M Pienta K Shah R Chinnaiyan A Integrative molecular concept modeling of prostate cancer progression. Nat Genet 2006 14 41 51 17173048 10.1038/ng1935

[B4] Mitelman F Johansson B Mertens F The impact of translocations and gene fusions on cancer causation. Nat Rev Cancer 2007 14 233 245 10.1038/nrc2091 17361217

[B5] Berger MF Lawrence MS Demichelis F Drier Y Cibulskis K Sivachenko AY Sboner A Esgueva R Pflueger D Sougnez C Onofrio R Carter SL Park K Habegger L Ambrogio L Fennell T Parkin M Saksena G Voet D Ramos AH Pugh TJ Wilkinson J Fisher S Winckler W Mahan S Ardlie K Baldwin J Simons JW Kitabayashi N MacDonald TY The genomic complexity of primary human prostate cancer. Nature 2011 14 214 220 10.1038/nature09744 21307934 PMC3075885

[B6] Stephens PJ Greenman CD Fu B Yang F Bignell GR Mudie LJ Pleasance ED Lau KW Beare D Stebbings LA McLaren S Lin ML McBride DJ Varela I Nik-Zainal S Leroy C Jia M Menzies A Butler AP Teague JW Quail MA Burton J Swerdlow H Carter NP Morsberger LA Iacobuzio-Donahue C Follows GA Green AR Flanagan AM Stratton MR Massive genomic rearrangement acquired in a single catastrophic event during cancer development. Cell 2011 14 27 40 10.1016/j.cell.2010.11.055 21215367 PMC3065307

[B7] Stephens PJ McBride DJ Lin ML Varela I Pleasance ED Simpson JT Stebbings LA Leroy C Edkins S Mudie LJ Greenman CD Jia M Latimer C Teague JW Lau KW Burton J Quail MA Swerdlow H Churcher C Natrajan R Sieuwerts AM Martens JW Silver DP Langerod A Russnes HE Foekens JA Reis-Filho JS van 't Veer L Richardson AL Borresen-Dale AL Complex landscapes of somatic rearrangement in human breast cancer genomes. Nature 2009 14 1005 1010 10.1038/nature08645 20033038 PMC3398135

[B8] Rausch T Jones DT Zapatka M Stutz AM Zichner T Weischenfeldt J Jager N Remke M Shih D Northcott PA Pfaff E Tica J Wang Q Massimi L Witt H Bender S Pleier S Cin H Hawkins C Beck C von Deimling A Hans V Brors B Eils R Scheurlen W Blake J Benes V Kulozik AE Witt O Martin D Genome sequencing of pediatric medulloblastoma links catastrophic DNA rearrangements with TP53 mutations. Cell 2012 14 59 71 10.1016/j.cell.2011.12.013 22265402 PMC3332216

[B9] Welch JS Ley TJ Link DC Miller CA Larson DE Koboldt DC Wartman LD Lamprecht TL Liu F Xia J Kandoth C Fulton RS McLellan MD Dooling DJ Wallis JW Chen K Harris CC Schmidt HK Kalicki-Veizer JM Lu C Zhang Q Lin L O'Laughlin MD McMichael JF Delehaunty KD Fulton LA Magrini VJ McGrath SD Demeter RT Vickery TL The origin and evolution of mutations in acute myeloid leukemia. Cell 2012 14 264 278 10.1016/j.cell.2012.06.023 22817890 PMC3407563

[B10] Chen K Wallis JW McLellan MD Larson DE Kalicki JM Pohl CS McGrath SD Wendl MC Zhang QY Locke DP Shi XQ Fulton RS Ley TJ Wilson RK Ding L Mardis ER BreakDancer: an algorithm for high-resolution mapping of genomic structural variation. Nat Methods 2009 14 677 U676 10.1038/nmeth.1363 19668202 PMC3661775

[B11] Hormozdiari F Alkan C Eichler EE Sahinalp SC Combinatorial algorithms for structural variation detection in high-throughput sequenced genomes. Genome Res 2009 14 1270 1278 10.1101/gr.088633.108 19447966 PMC2704429

[B12] Wang J Mullighan C Easton J Roberts S Heatley S Ma J Rusch M Chen K Harris C Ding L Holmfeldt L Payne-Turner D Fan X Wei L Zhao D Obenauer J Naeve C Mardis E Wilson R Downing J Zhang J CREST maps somatic structural variation in cancer genomes with base-pair resolution. Nat Methods 2011 14 652 654 10.1038/nmeth.1628 21666668 PMC3527068

[B13] Alkan C Coe BP Eichler EE Genome structural variation discovery and genotyping. Nat Rev Genet 2011 14 363 376 10.1038/nrg2958 21358748 PMC4108431

[B14] Greenman CD Pleasance ED Newman S Yang F Fu B Nik-Zainal S Jones D Lau KW Carter N Edwards PA Futreal PA Stratton MR Campbell PJ Estimation of rearrangement phylogeny for cancer genomes. Genome Res 2011 14 346 361 21994251 10.1101/gr.118414.110 PMC3266042

[B15] Raphael BJ Volik S Collins C Pevzner PA Reconstructing tumor genome architectures. Bioinformatics 2003 19 Suppl 2 ii162 171 14534186 10.1093/bioinformatics/btg1074

[B16] Kim YK Bae GU Kang JK Park JW Lee EK Lee HY Choi WS Lee HW Han JW Cooperation of H2O2-mediated ERK activation with Smad pathway in TGF-beta1 induction of p21WAF1/Cip1. Cell Signal 2006 14 236 243 10.1016/j.cellsig.2005.04.008 15979845

[B17] McPherson A Hormozdiari F Zayed A Giuliany R Ha G Sun MG Griffith M Heravi Moussavi A Senz J Melnyk N Pacheco M Marra MA Hirst M Nielsen TO Sahinalp SC Huntsman D Shah SP deFuse: an algorithm for gene fusion discovery in tumor RNA-Seq data. PLoS Comput Biol 2011 14 e1001138 10.1371/journal.pcbi.1001138 21625565 PMC3098195

[B18] Wang K Singh D Zeng Z Coleman SJ Huang Y Savich GL He X Mieczkowski P Grimm SA Perou CM MacLeod JN Chiang DY Prins JF Liu J MapSplice: accurate mapping of RNA-seq reads for splice junction discovery. Nucleic Acids Res 2010 14 e178 10.1093/nar/gkq622 20802226 PMC2952873

[B19] Chen K Wallis JW Kandoth C Kalicki-Veizer JM Mungall KL Mungall AJ Jones SJ Marra MA Ley TJ Mardis ER Wilson RK Weinstein JN Ding L BreakFusion: targeted assembly-based identification of gene fusions in whole transcriptome paired-end sequencing data. Bioinformatics 2012 14 1923 1924 10.1093/bioinformatics/bts272 22563071 PMC3389765

[B20] Sboner A Karpikov A Chen GX Smith M Mattoon D Freeman-Cook L Schweitzer B Gerstein MB Robust-linear-model normalization to reduce technical variability in functional protein microarrays. J Proteome Res 2010 14 636 636 10.1021/pr900412k 19817483

[B21] Bignell GR Santarius T Pole JC Butler AP Perry J Pleasance E Greenman C Menzies A Taylor S Edkins S Campbell P Quail M Plumb B Matthews L McLay K Edwards PA Rogers J Wooster R Futreal PA Stratton MR Architectures of somatic genomic rearrangement in human cancer amplicons at sequence-level resolution. Genome Res 2007 14 1296 1303 10.1101/gr.6522707 17675364 PMC1950898

[B22] Inaki K Hillmer AM Ukil L Yao F Woo XY Vardy LA Zawack KF Lee CW Ariyaratne PN Chan YS Desai KV Bergh J Hall P Putti TC Ong WL Shahab A Cacheux-Rataboul V Karuturi RK Sung WK Ruan X Bourque G Ruan Y Liu ET Transcriptional consequences of genomic structural aberrations in breast cancer. Genome Res 2011 14 676 687 10.1101/gr.113225.110 21467264 PMC3083084

[B23] Alkan C Sajjadian S Eichler EE Limitations of next-generation genome sequence assembly. Nat Methods 2011 14 61 65 10.1038/nmeth.1527 21102452 PMC3115693

[B24] McPherson A Wu C Hajirasouliha I Hormozdiari F Hach F Lapuk A Volik S Shah S Collins C Sahinalp SC Comrad: detection of expressed rearrangements by integrated analysis of RNA-Seq and low coverage genome sequence data. Bioinformatics 2011 14 1481 1488 10.1093/bioinformatics/btr184 21478487

[B25] McPherson A Wu C Wyatt AW Shah S Collins C Sahinalp SC nFuse: discovery of complex genomic rearrangements in cancer using high-throughput sequencing. Genome Res 2012 14 2250 2261 10.1101/gr.136572.111 22745232 PMC3483554

[B26] Li H Durbin R Fast and accurate short read alignment with Burrows-Wheeler transform. Bioinformatics 2009 14 1754 1760 10.1093/bioinformatics/btp324 19451168 PMC2705234

[B27] Edgren H Murumagi A Kangaspeska S Nicorici D Hongisto V Kleivi K Rye IH Nyberg S Wolf M Borresen-Dale AL Kallioniemi O Identification of fusion genes in breast cancer by paired-end RNA-sequencing. Genome Biol 2011 14 R6 10.1186/gb-2011-12-1-r6 21247443 PMC3091304

[B28] Kim D Salzberg SL TopHat-Fusion: an algorithm for discovery of novel fusion transcripts. Genome Biol 2011 14 R72 10.1186/gb-2011-12-8-r72 21835007 PMC3245612

[B29] Wang J Mullighan CG Easton J Roberts S Heatley SL Ma J Rusch MC Chen K Harris CC Ding L Holmfeldt L Payne-Turner D Fan X Wei L Zhao D Obenauer JC Naeve C Mardis ER Wilson RK Downing JR Zhang J CREST maps somatic structural variation in cancer genomes with base-pair resolution. Nat Methods 2011 14 652 654 10.1038/nmeth.1628 21666668 PMC3527068

[B30] Asmann YW Hossain A Necela BM Middha S Kalari KR Sun Z Chai HS Williamson DW Radisky D Schroth GP Kocher JP Perez EA Thompson EA A novel bioinformatics pipeline for identification and characterization of fusion transcripts in breast cancer and normal cell lines. Nucleic Acids Res 2011 14 e100 10.1093/nar/gkr362 21622959 PMC3159479

[B31] Agarwal N Adhikari AS Iyer SV Hekmatdoost K Welch DR Iwakuma T MTBP suppresses cell migration and filopodia formation by inhibiting ACTN4. Oncogene 2012 14 462 470 22370640 10.1038/onc.2012.69 PMC3742333

[B32] Montero JC Seoane S Ocana A Pandiella A P-Rex1 participates in Neuregulin-ErbB signal transduction and its expression correlates with patient outcome in breast cancer. Oncogene 2011 14 1059 1071 10.1038/onc.2010.489 21042280

[B33] Sosa MS Lopez-Haber C Yang C Wang H Lemmon MA Busillo JM Luo J Benovic JL Klein-Szanto A Yagi H Gutkind JS Parsons RE Kazanietz MG Identification of the Rac-GEF P-Rex1 as an essential mediator of ErbB signaling in breast cancer. Mol Cell 2010 14 877 892 10.1016/j.molcel.2010.11.029 21172654 PMC3038344

[B34] Comprehensive molecular portraits of human breast tumours. Nature 2012 14 61 70 10.1038/nature11412 23000897 PMC3465532

[B35] Robinson DR Kalyana-Sundaram S Wu YM Shankar S Cao X Ateeq B Asangani IA Iyer M Maher CA Grasso CS Lonigro RJ Quist M Siddiqui J Mehra R Jing X Giordano TJ Sabel MS Kleer CG Palanisamy N Natrajan R Lambros MB Reis-Filho JS Kumar-Sinha C Chinnaiyan AM Functionally recurrent rearrangements of the MAST kinase and Notch gene families in breast cancer. Nat Med 2011 14 1646 1651 10.1038/nm.2580 22101766 PMC3233654

[B36] A map of human genome variation from population-scale sequencing. Nature 2010 14 1061 1073 10.1038/nature09534 20981092 PMC3042601

[B37] Roychowdhury S Iyer MK Robinson DR Lonigro RJ Wu YM Cao X Kalyana-Sundaram S Sam L Balbin OA Quist MJ Barrette T Everett J Siddiqui J Kunju LP Navone N Araujo JC Troncoso P Logothetis CJ Innis JW Smith DC Lao CD Kim SY Roberts JS Gruber SB Pienta KJ Talpaz M Chinnaiyan AM Personalized oncology through integrative high-throughput sequencing: a pilot study. Sci Transl Med 2011 14 111ra121 10.1126/scitranslmed.3003161 22133722 PMC3476478

[B38] Ding L Ley T Larson DE Miller CA Koboldt DC Welch JS Ritchey JK Young MA Lamprecht T McLellan MD McMichael JF Wallis J Lu C Shen D Harris CC Dooling DJ Fulton RS Fulton LL Chen K Schmidt H Kalicki-Veizer J Magrini V Cook L McGrath SD Vickery TL Wendl MC Heath S Watson MA Link DC Tomasson MH Clonal evolution in relapsed acute myeloid leukaemia revealed by whole-genome sequencing. Nature 2012 14 506 510 10.1038/nature10738 22237025 PMC3267864

[B39] Navin N Kendall J Troge J Andrews P Rodgers L McIndoo J Cook K Stepansky A Levy D Esposito D Muthuswamy L Krasnitz A McCombie WR Hicks J Wigler M Tumour evolution inferred by single-cell sequencing. Nature 2011 14 90 94 10.1038/nature09807 21399628 PMC4504184

[B40] Pevzner P Computational Molecular Biology: An Algorithmic Approach 2000 Cambridge, MA: MIT Press

[B41] Handsaker RE Korn JM Nemesh J McCarroll SA Discovery and genotyping of genome structural polymorphism by sequencing on a population scale. Nat Genet 2011 14 269 276 10.1038/ng.768 21317889 PMC5094049

[B42] BreakTrans. http://bioinformatics.mdanderson.org/main/BreakTrans

[B43] The Cancer Genome Atlas. http://cancergenome.nih.gov/

