# Supplementary material for: BreakTrans: uncovering the genomic architecture of gene fusions
Source: Genome Biol. 2013 Aug 23;14(8):R87. doi: 10.1186/gb-2013-14-8-r87 (PMC4054677; doi:10.1186/gb-2013-14-8-r87)
Supplement: Additional file 5 — Figures S1 to S42. Figure S1: PREX1-CPNE1 fusion detected from SK-BR-3 WTS data. Figures S2 to S5: integrative genomics views (IGVs) of read alignments at the two GSR breakpoints (four break-ends) underlying PREX1-CPNE1. Figures S6 to S9: IGVs of GSR breakpoints underlying MTBP-SAMD12. Figures S10 and S11: IGVs of GSR breakpoints underlying WDR67-ZNF704. Figure S12: whole genome somatic copy number alteration (log2) of A09I. Figure S13: the NF1-NLE1 fusions detected in A09I. Figure S14: somatic copy number alteration on chromosome 17 of A09I with red vertical lines marking the GSR breakpoints that support the NF1-NLE1 fusion. Figures S15 to S18: IGV of the two GSR breakpoints (four break-ends) that underlie NF1-NLE1. Figure S19: the PPP1R1B-PIPOX fusion detected in A0D1. Figure S20: whole-genome somatic copy number alteration (log2) of A0D1. Figure S21: somatic copy number alteration on chromosome 17 of A0D1 with red vertical lines marking the GSR breakpoints that support the PPP1R1B-PIPOX fusion. Figures S22 to S27: IGVs of the three GSR breakpoints (6 break-ends) that underlie PPP1R1B-PIPOX. Figure S28: the PPP3R1-TTC27 fusion detected in A0YG. Figure S29: whole-genome somatic copy number alteration (log2) of A0YG. Figure S30: somatic copy number alteration on chromosome 2 of A0YG with red vertical lines marking the GSR breakpoints that support the PPP3R1-TTC27 fusion. Figure S31: zoomed-in view of Figure S29 at the chromosome 2 chromothripsis that harbors the fusion. Figures S32 to S37: IGVs of the three GSR breakpoints (6 break-ends) that underlie PPP3R1-TTC27. Figures S38 to S41: PCR validation of TCGA BRCA genomic breakpoints. Figure S42: capillary sequencing trace of a PCR product that was not visible in the gel. [file gb-2013-14-8-r87-S5.PPTX]

## Slide 1
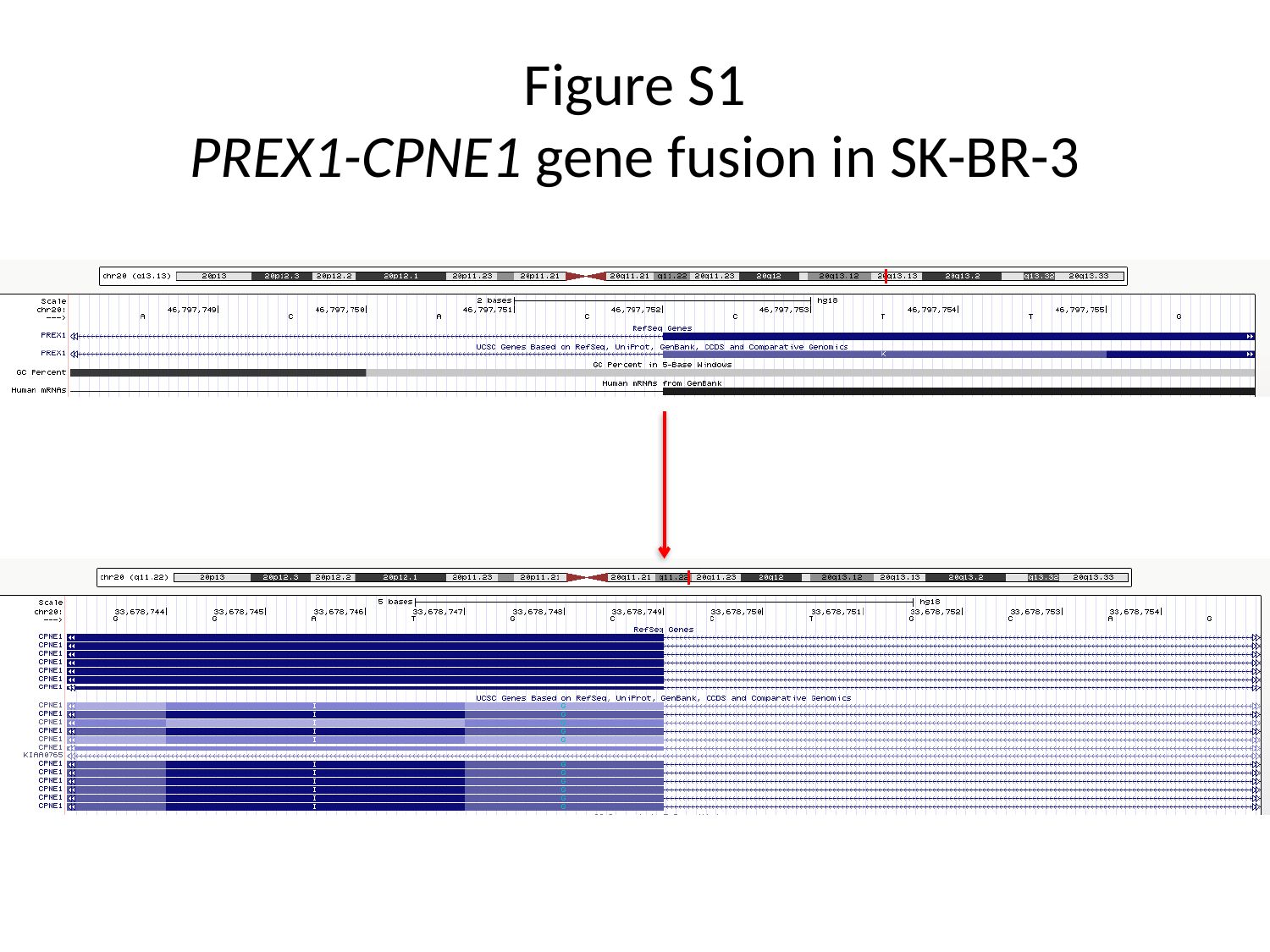

# Figure S1 PREX1-CPNE1 gene fusion in SK-BR-3

## Slide 2
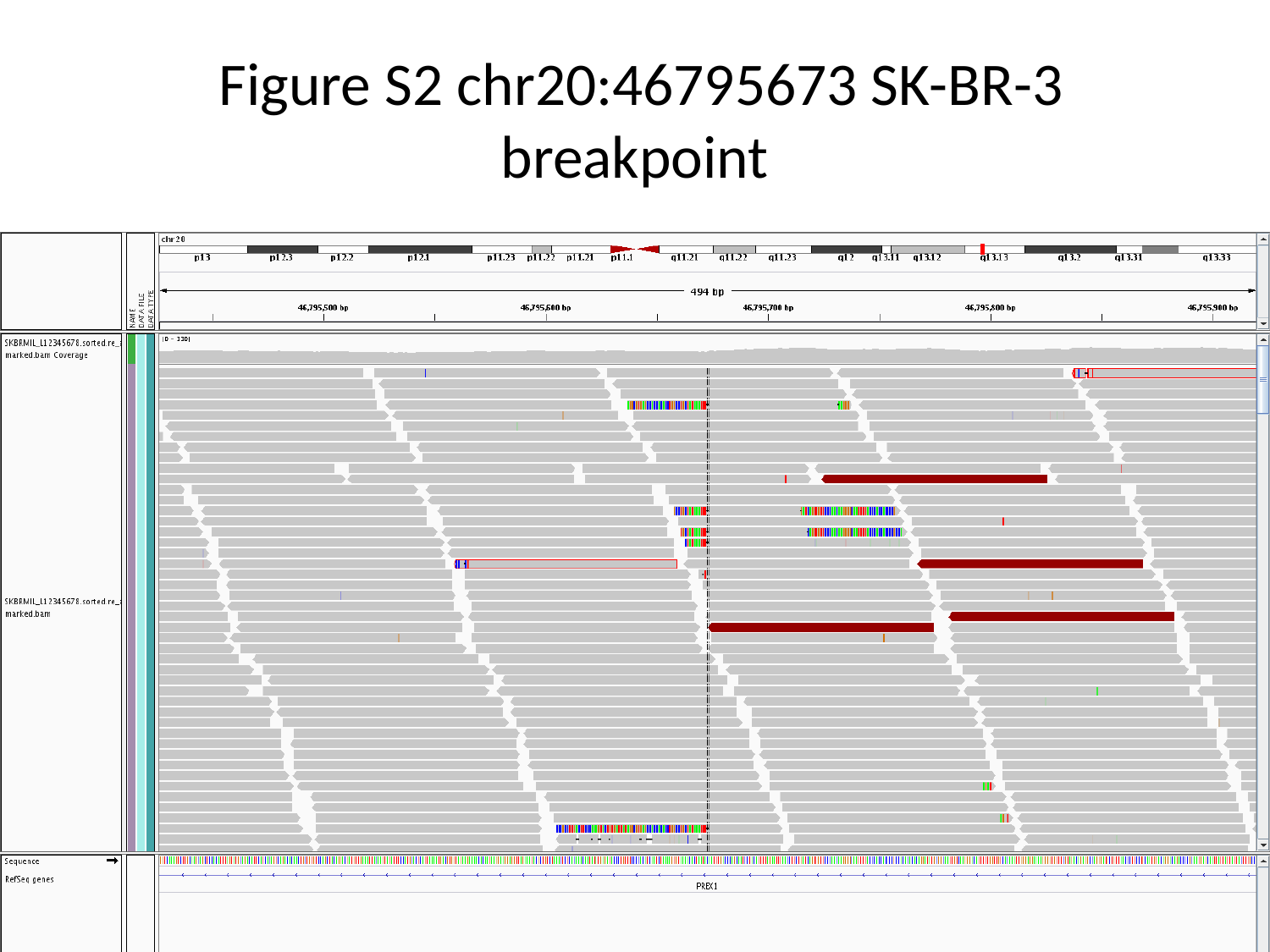

# Figure S2 chr20:46795673 SK-BR-3 breakpoint

## Slide 3
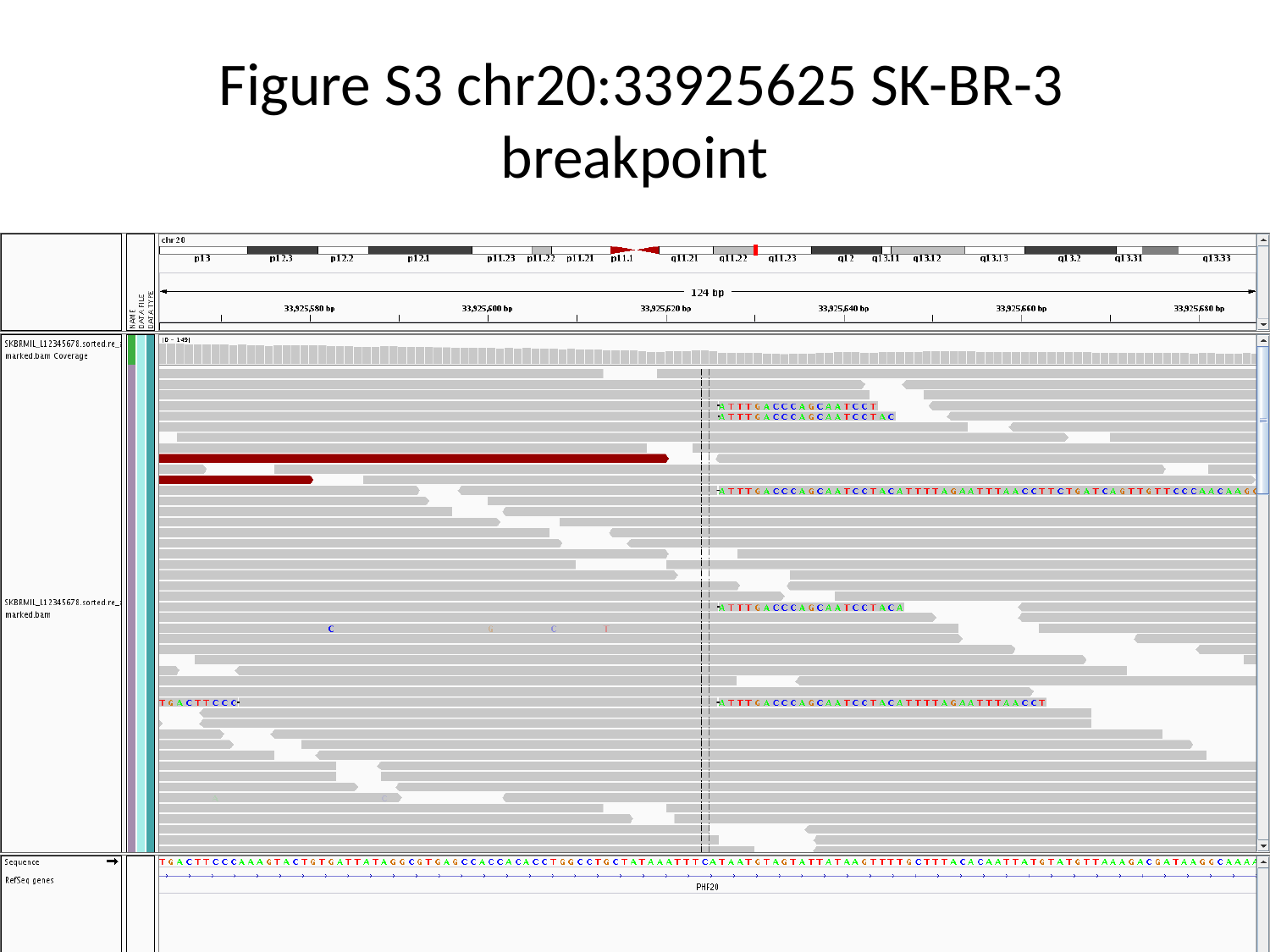

# Figure S3 chr20:33925625 SK-BR-3 breakpoint

## Slide 4
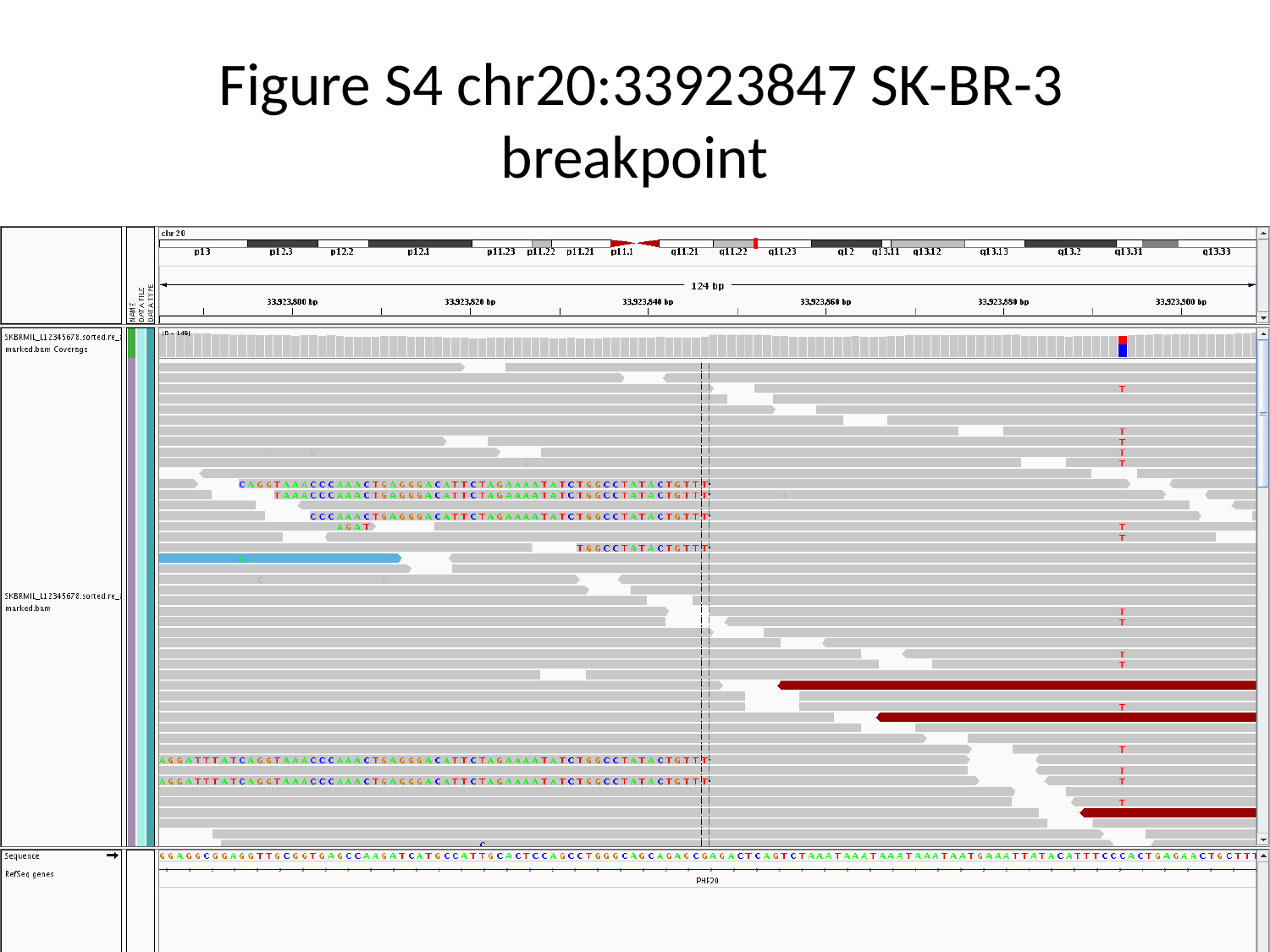

# Figure S4 chr20:33923847 SK-BR-3 breakpoint

## Slide 5
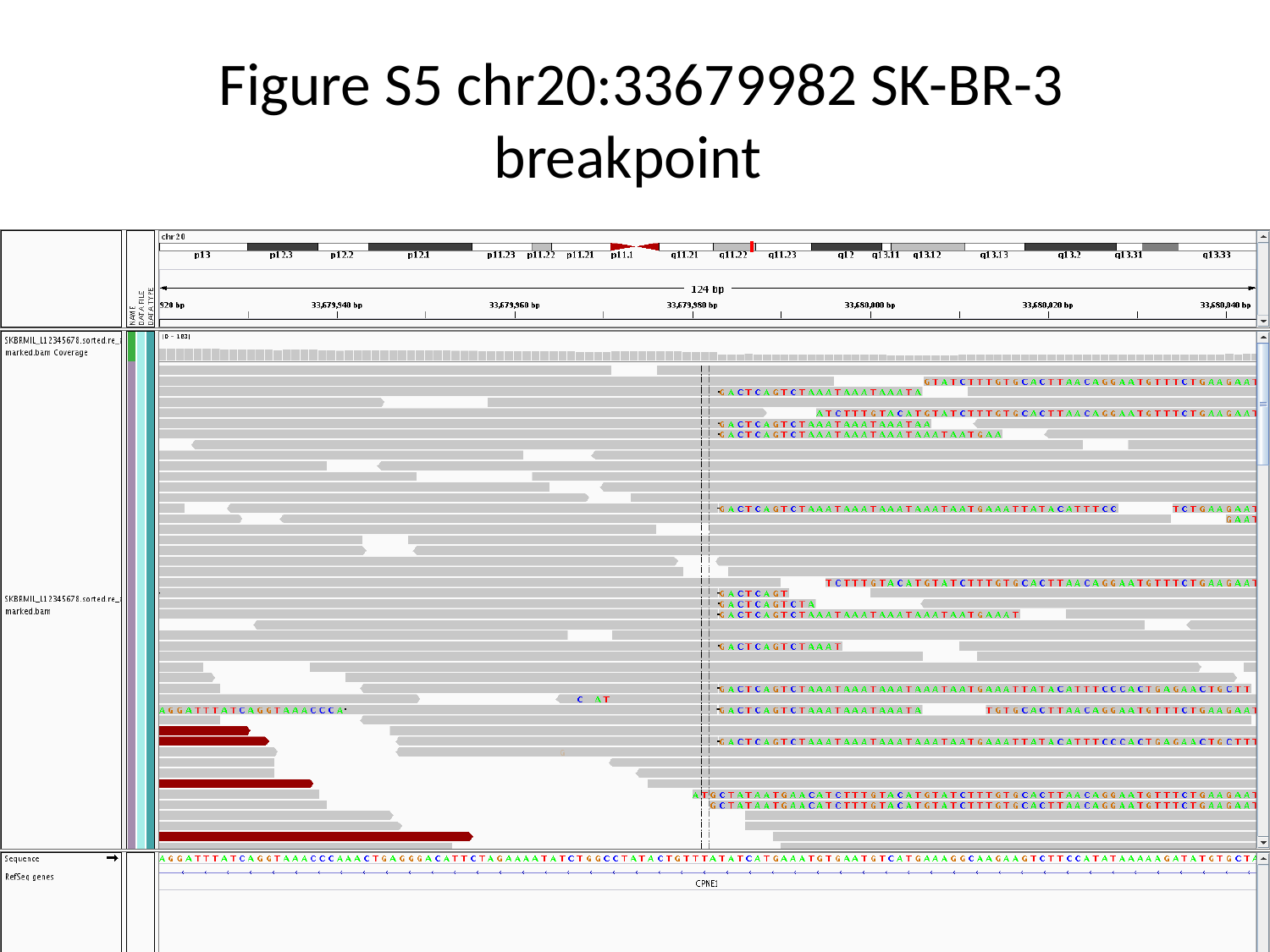

# Figure S5 chr20:33679982 SK-BR-3 breakpoint

## Slide 6
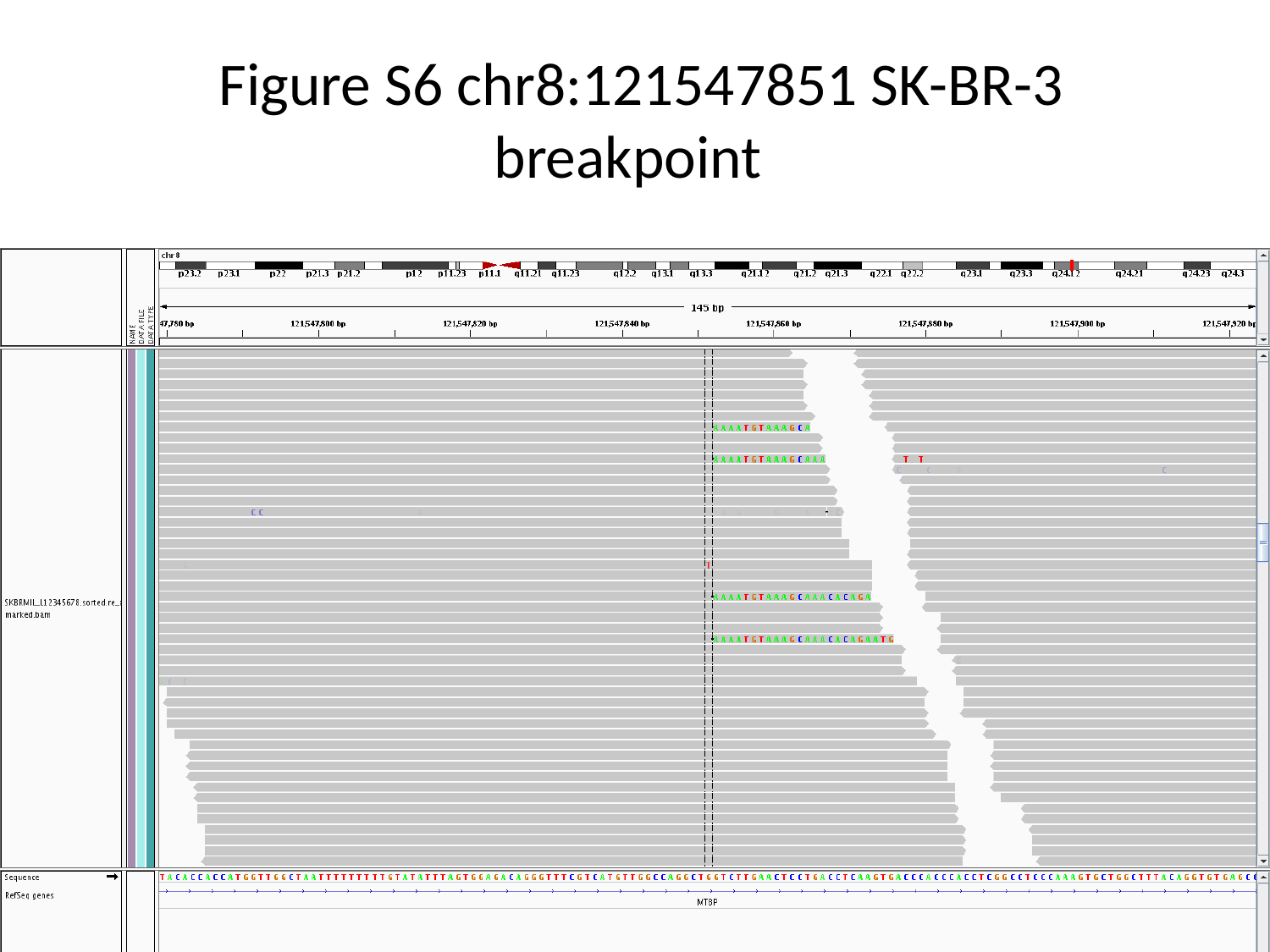

# Figure S6 chr8:121547851 SK-BR-3 breakpoint

## Slide 7
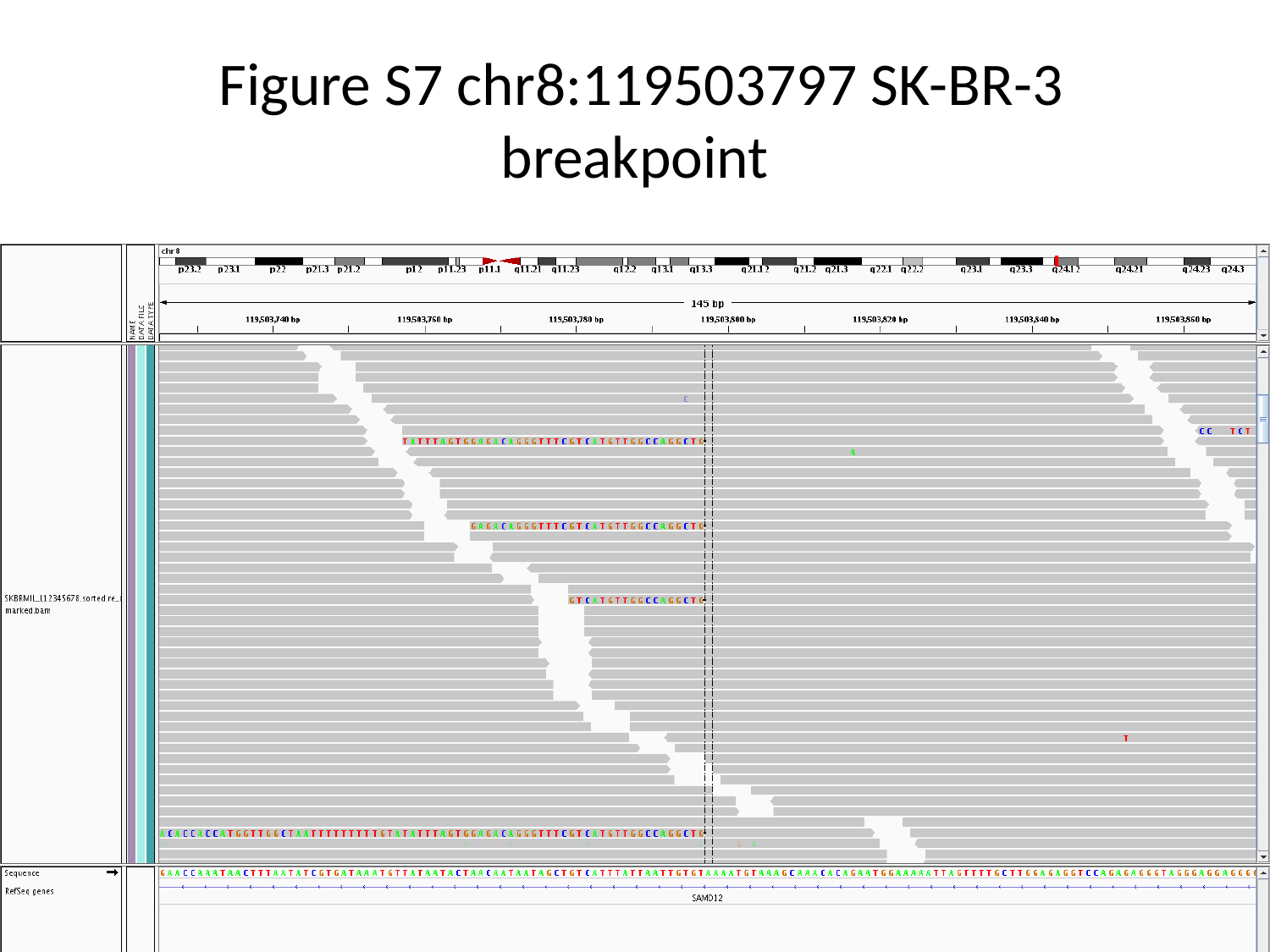

# Figure S7 chr8:119503797 SK-BR-3 breakpoint

## Slide 8
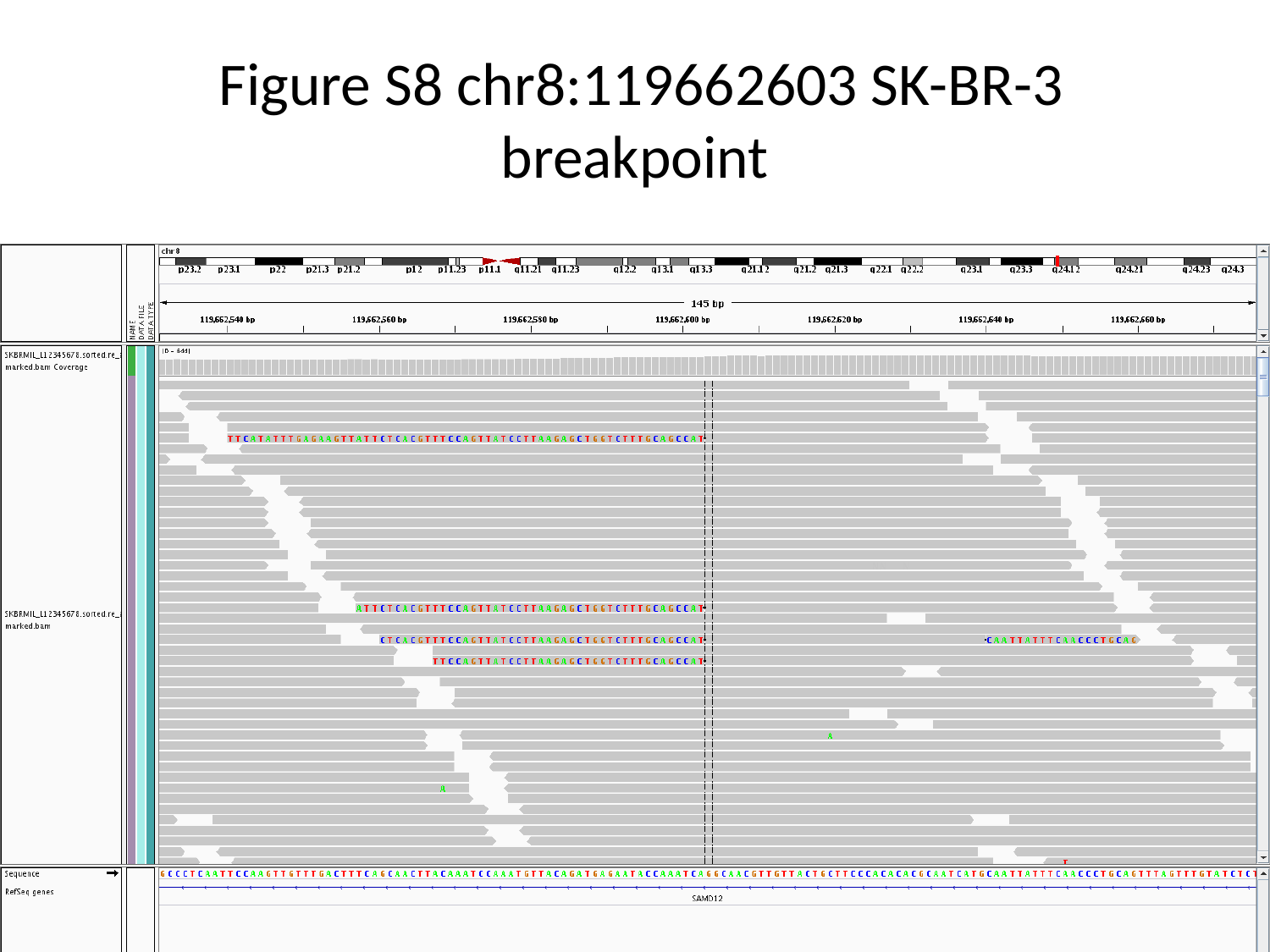

# Figure S8 chr8:119662603 SK-BR-3 breakpoint

## Slide 9
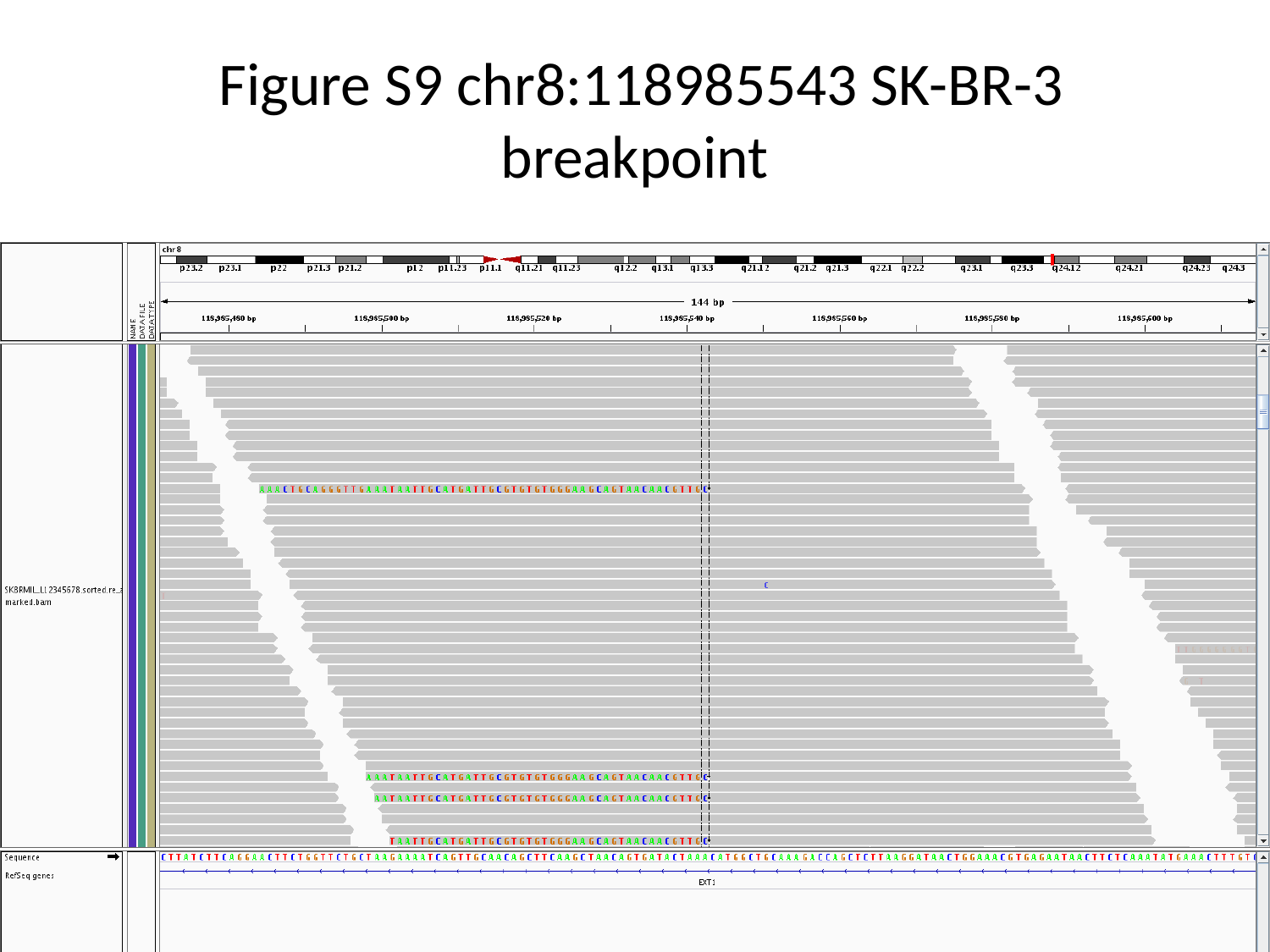

# Figure S9 chr8:118985543 SK-BR-3 breakpoint

## Slide 10
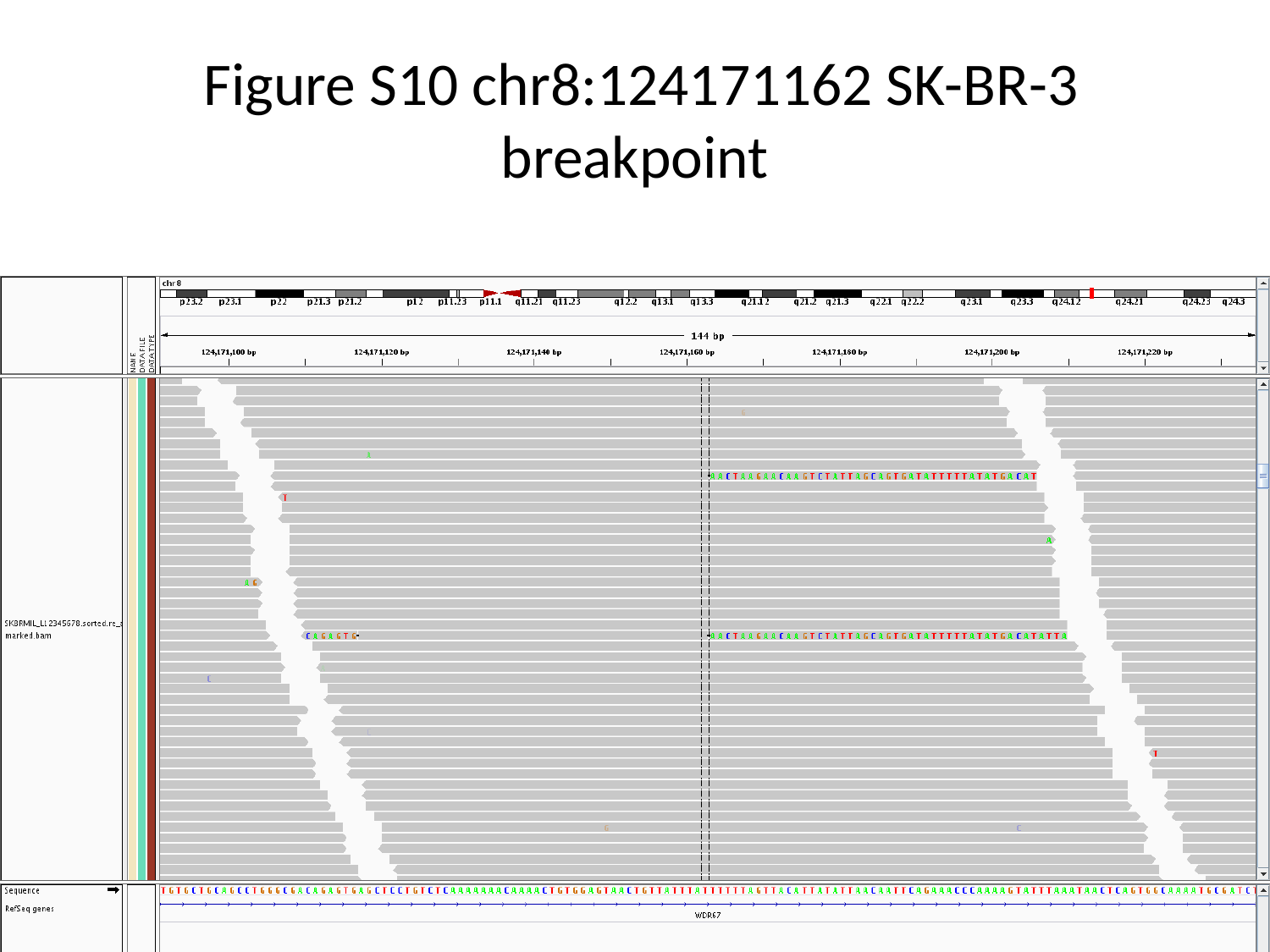

# Figure S10 chr8:124171162 SK-BR-3 breakpoint

## Slide 11
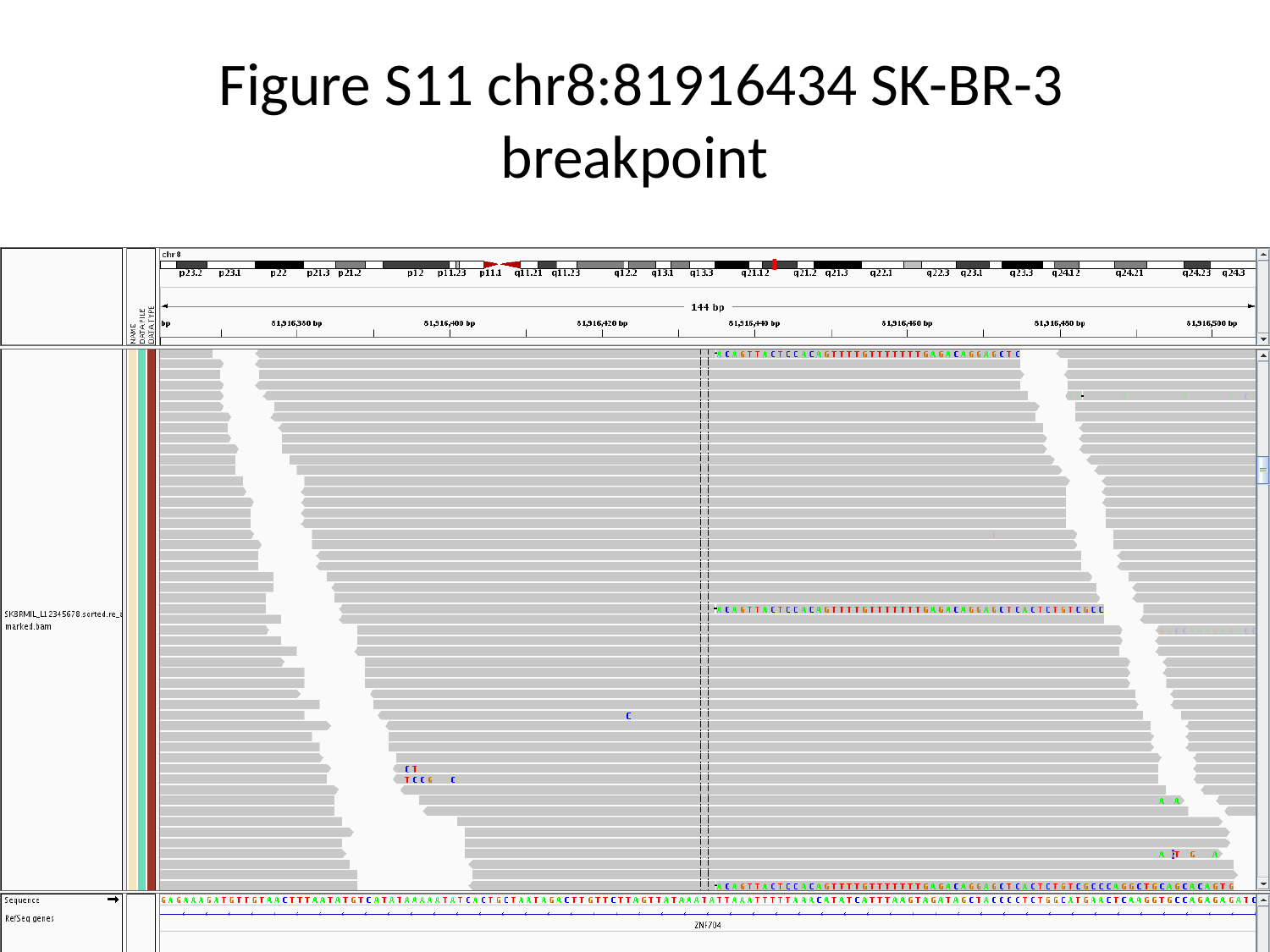

# Figure S11 chr8:81916434 SK-BR-3 breakpoint

## Slide 12
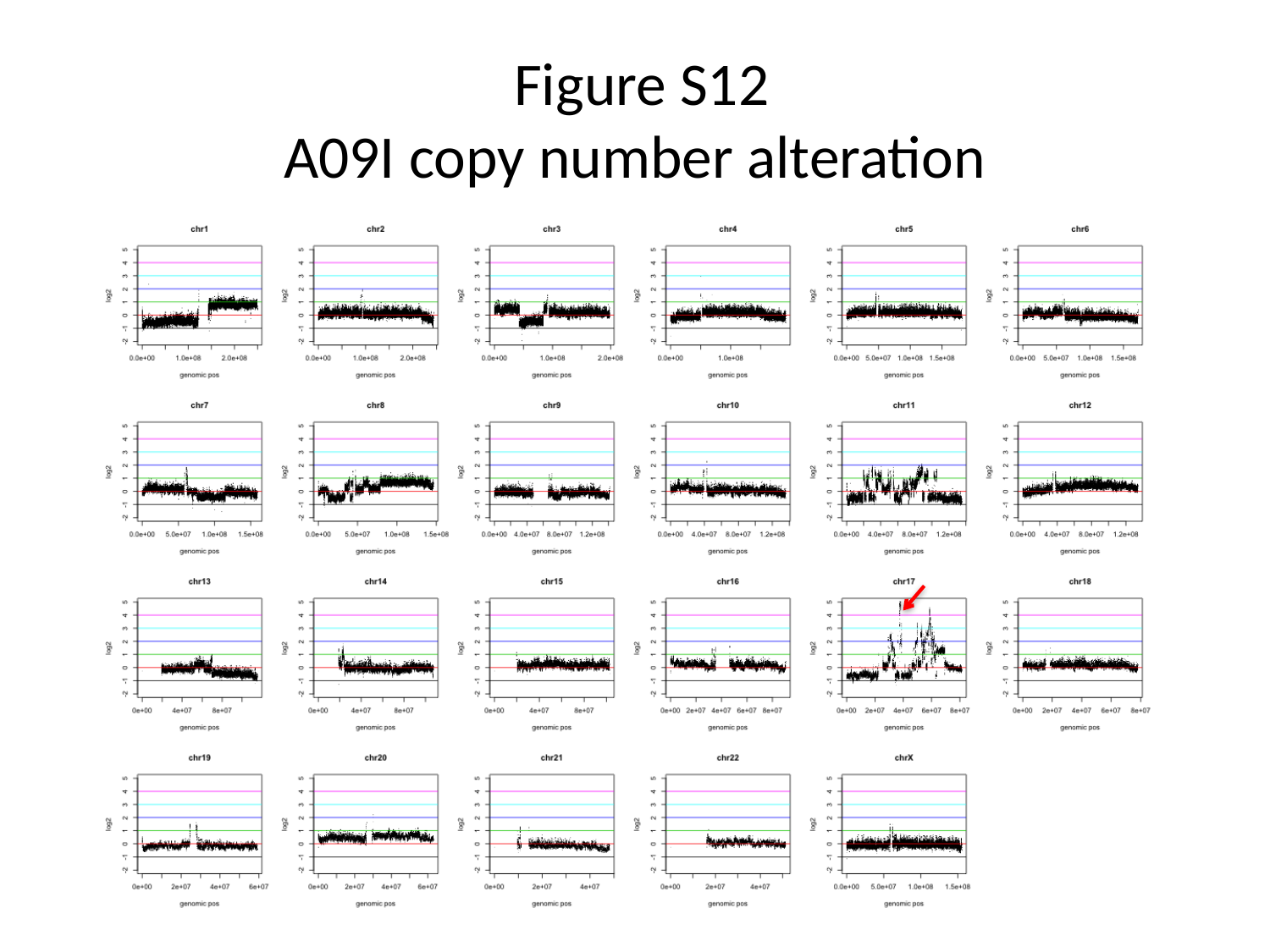

# Figure S12 A09I copy number alteration

## Slide 13
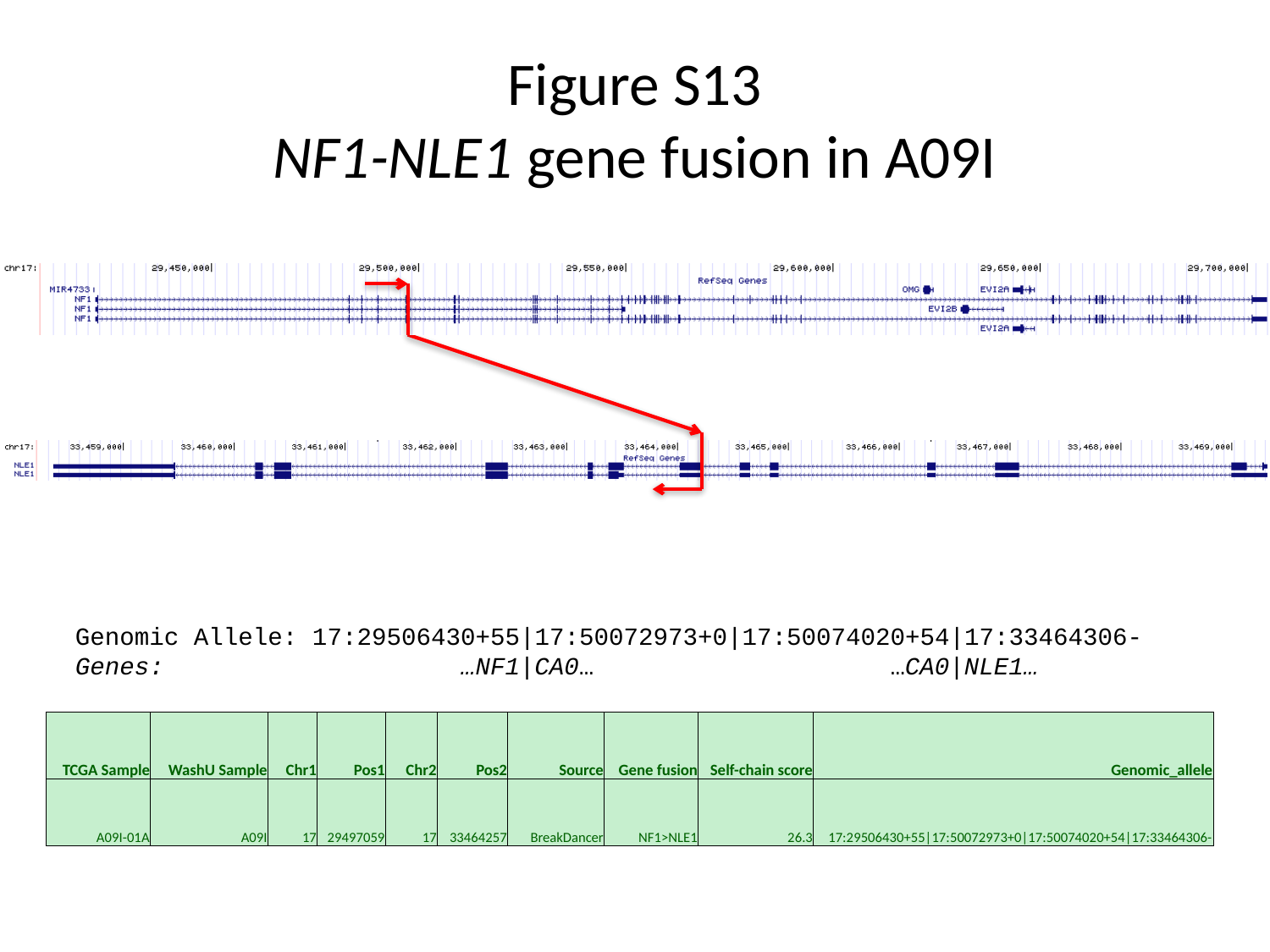

# Figure S13 NF1-NLE1 gene fusion in A09I
Genomic Allele: 17:29506430+55|17:50072973+0|17:50074020+54|17:33464306-
Genes: …NF1|CA0… …CA0|NLE1…
| TCGA Sample | WashU Sample | Chr1 | Pos1 | Chr2 | Pos2 | Source | Gene fusion | Self-chain score | Genomic\_allele |
| --- | --- | --- | --- | --- | --- | --- | --- | --- | --- |
| A09I-01A | A09I | 17 | 29497059 | 17 | 33464257 | BreakDancer | NF1>NLE1 | 26.3 | 17:29506430+55|17:50072973+0|17:50074020+54|17:33464306- |

## Slide 14
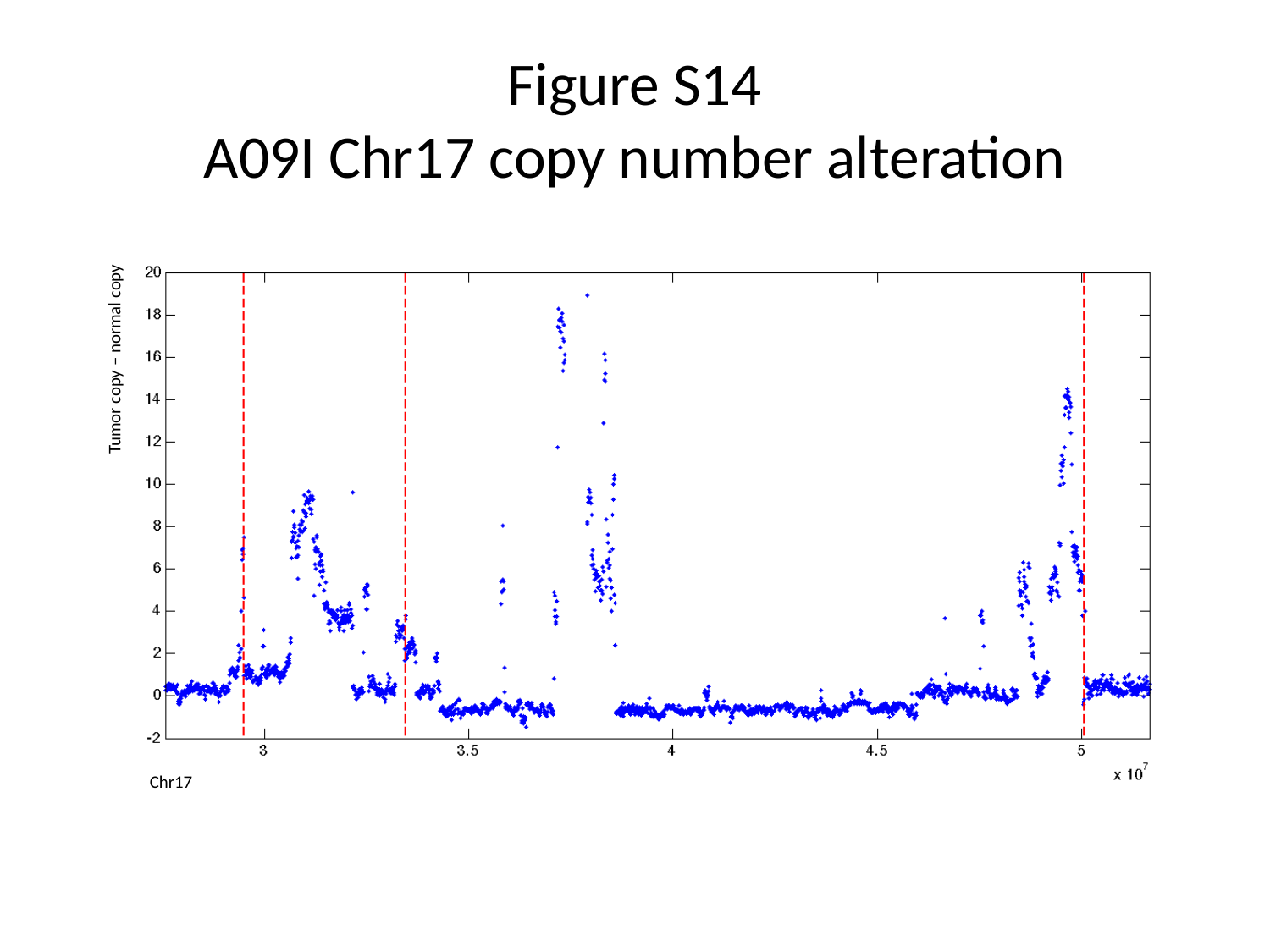

# Figure S14 A09I Chr17 copy number alteration
Tumor copy – normal copy
Chr17

## Slide 15
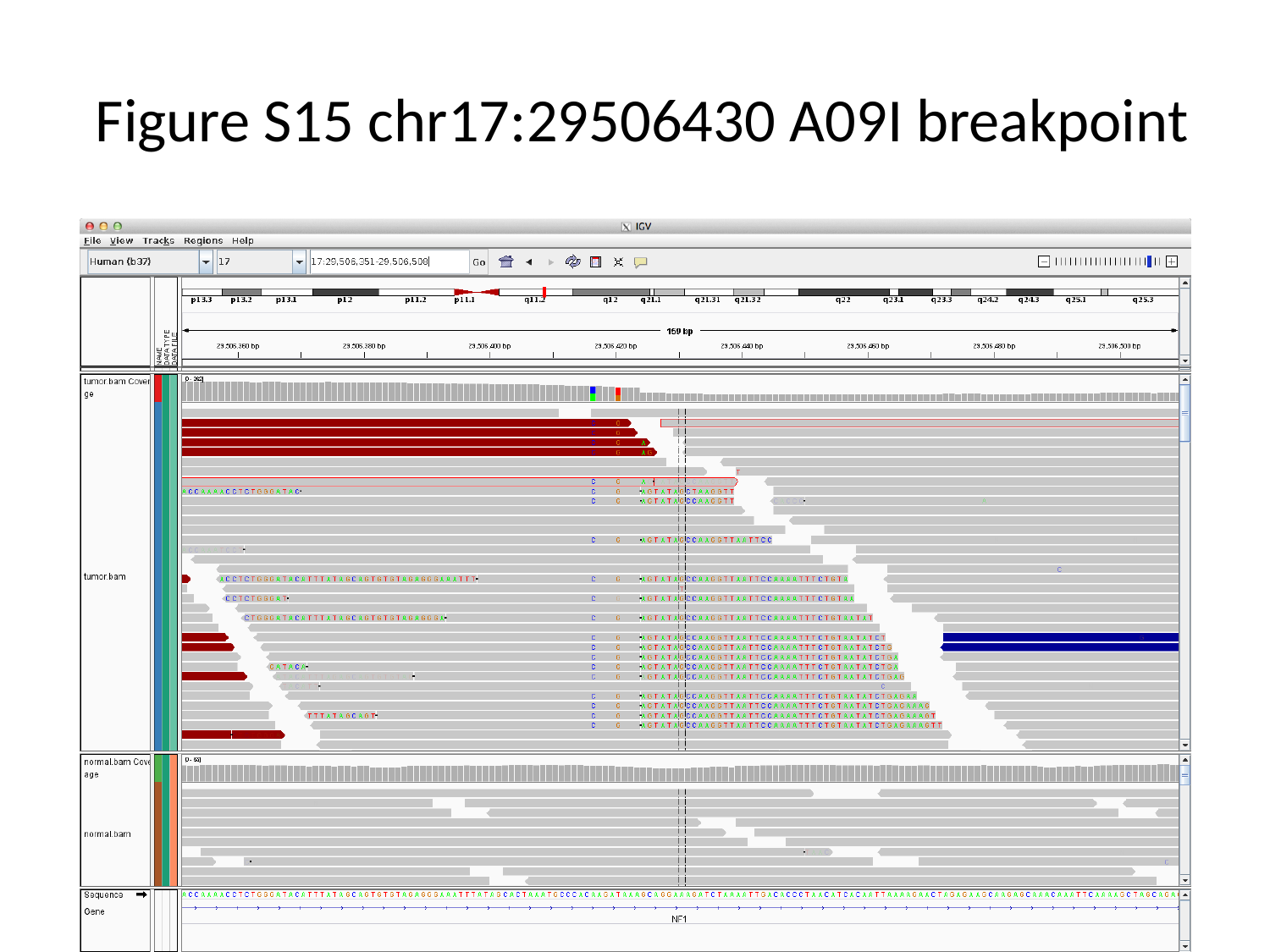

# Figure S15 chr17:29506430 A09I breakpoint

## Slide 16
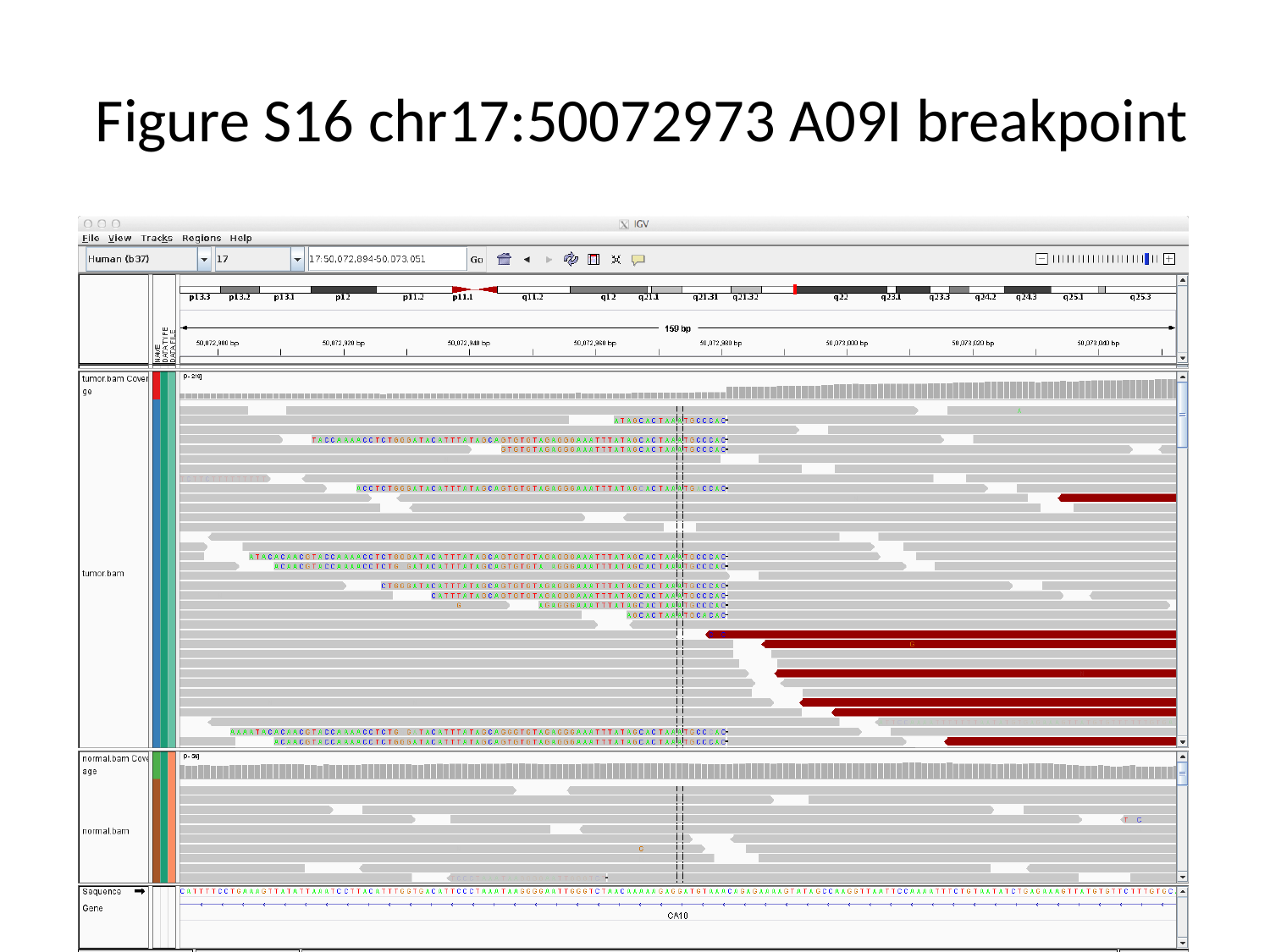

# Figure S16 chr17:50072973 A09I breakpoint

## Slide 17
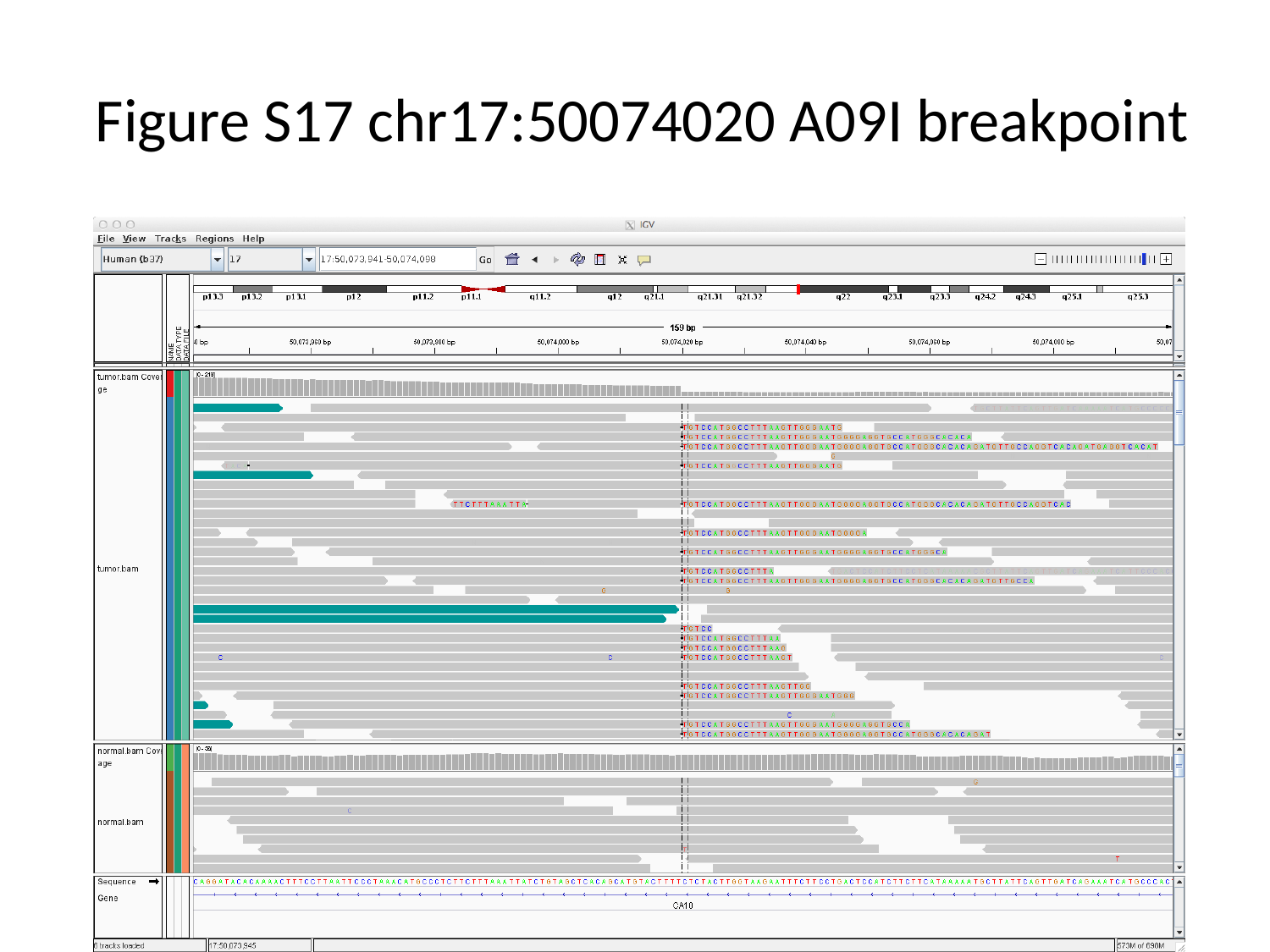

# Figure S17 chr17:50074020 A09I breakpoint

## Slide 18
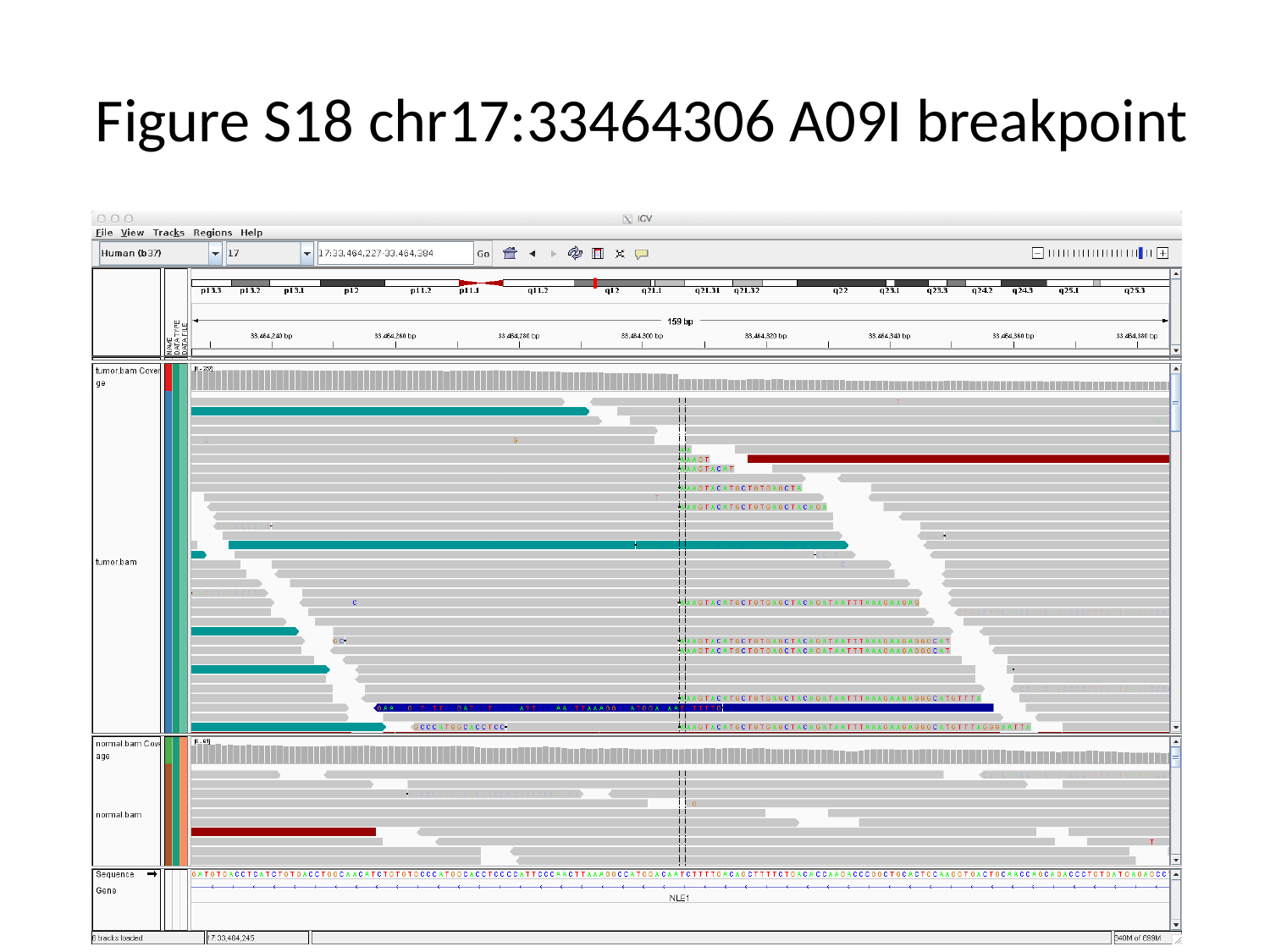

# Figure S18 chr17:33464306 A09I breakpoint

## Slide 19
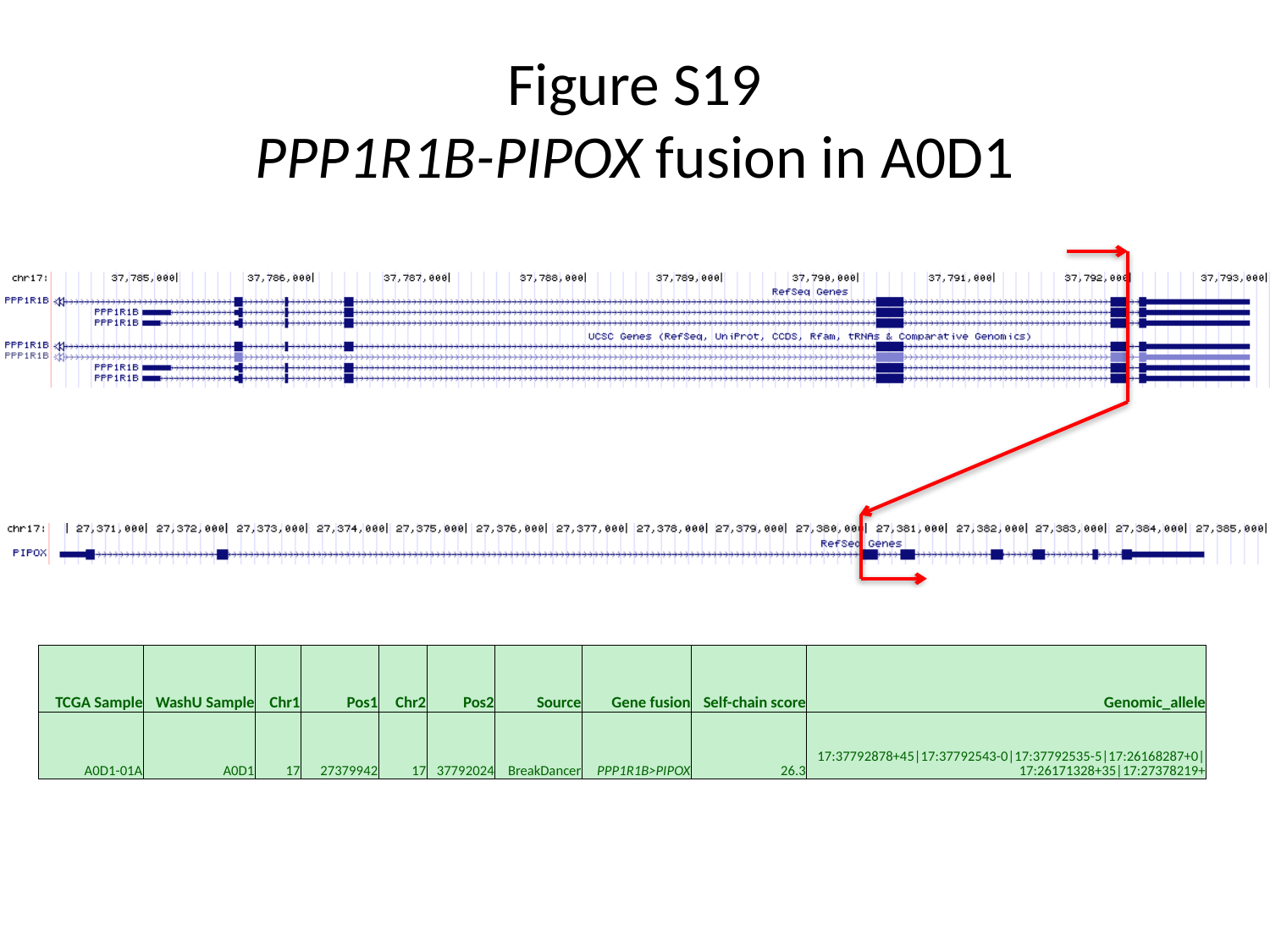

# Figure S19 PPP1R1B-PIPOX fusion in A0D1
| TCGA Sample | WashU Sample | Chr1 | Pos1 | Chr2 | Pos2 | Source | Gene fusion | Self-chain score | Genomic\_allele |
| --- | --- | --- | --- | --- | --- | --- | --- | --- | --- |
| A0D1-01A | A0D1 | 17 | 27379942 | 17 | 37792024 | BreakDancer | PPP1R1B>PIPOX | 26.3 | 17:37792878+45|17:37792543-0|17:37792535-5|17:26168287+0|17:26171328+35|17:27378219+ |

## Slide 20
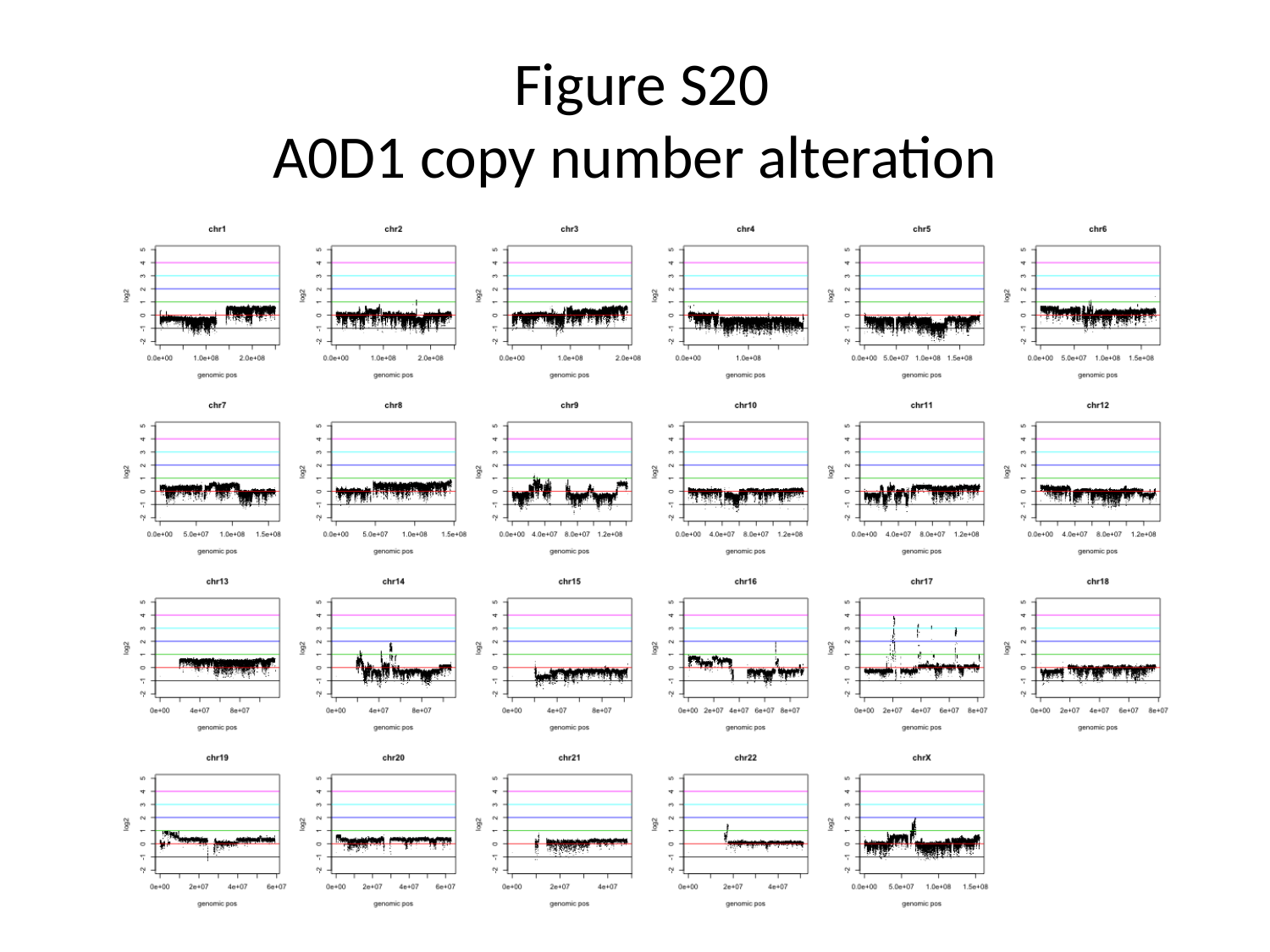

# Figure S20 A0D1 copy number alteration

## Slide 21
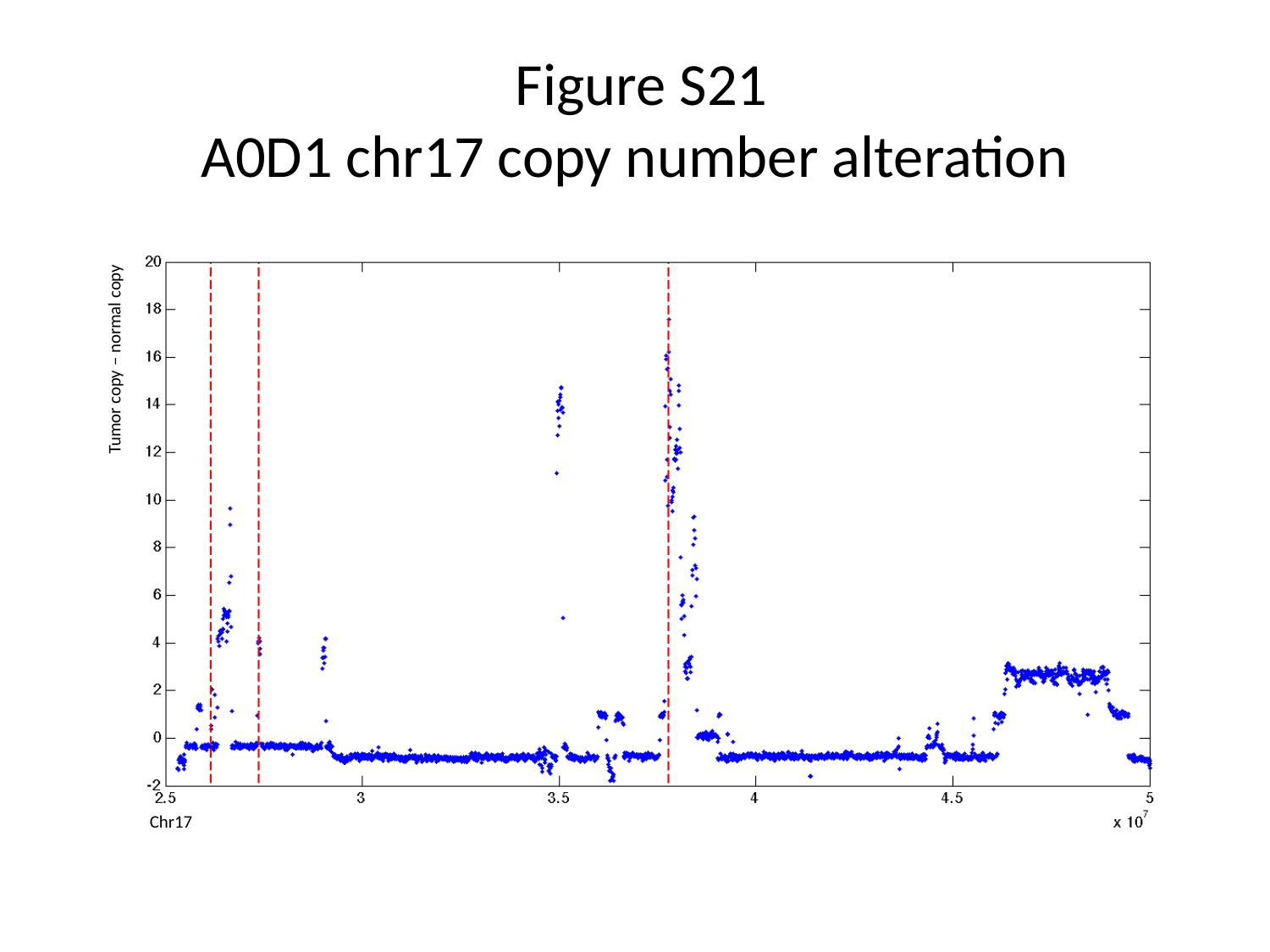

# Figure S21 A0D1 chr17 copy number alteration
Tumor copy – normal copy
Chr17

## Slide 22
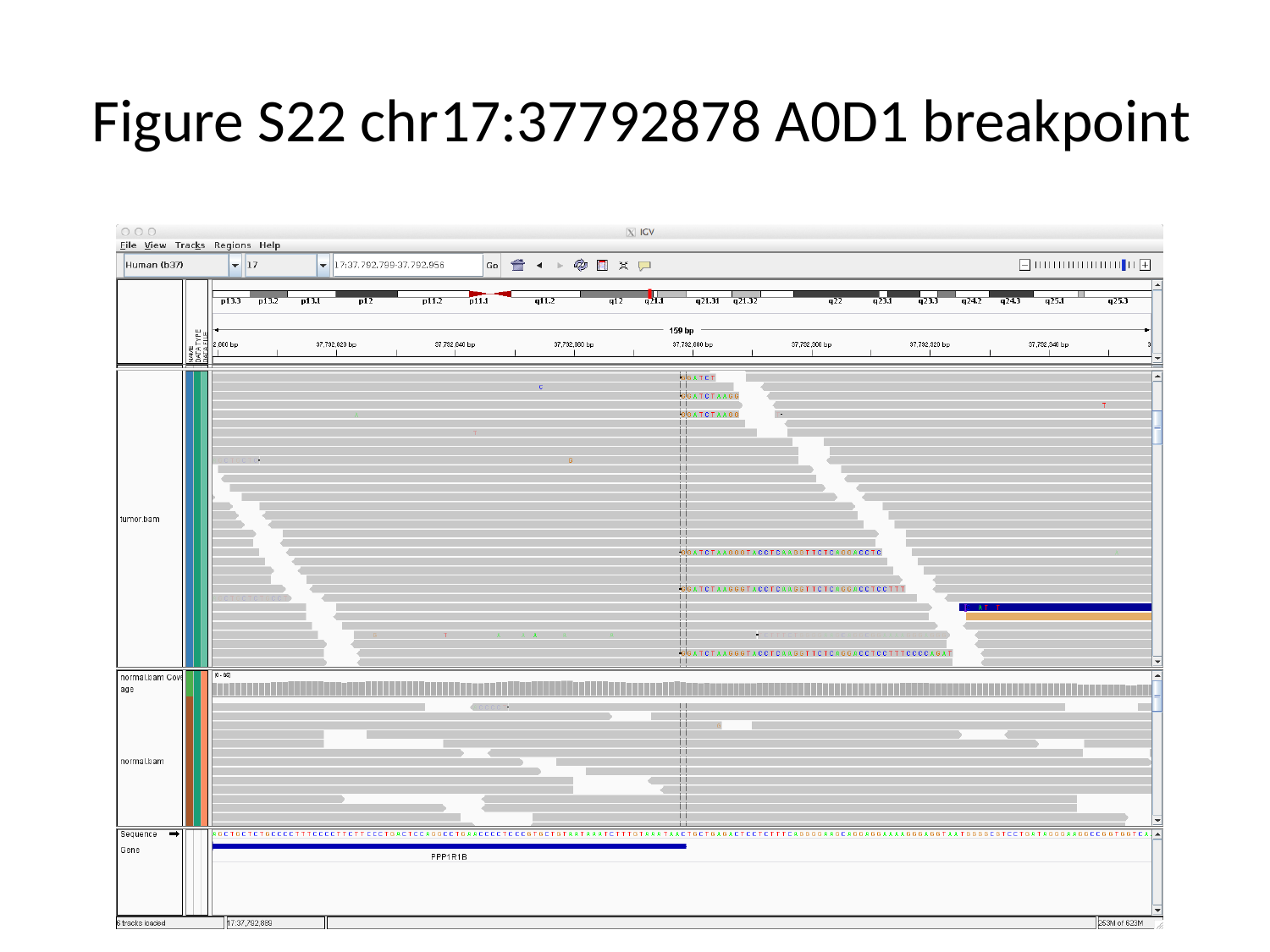

# Figure S22 chr17:37792878 A0D1 breakpoint

## Slide 23
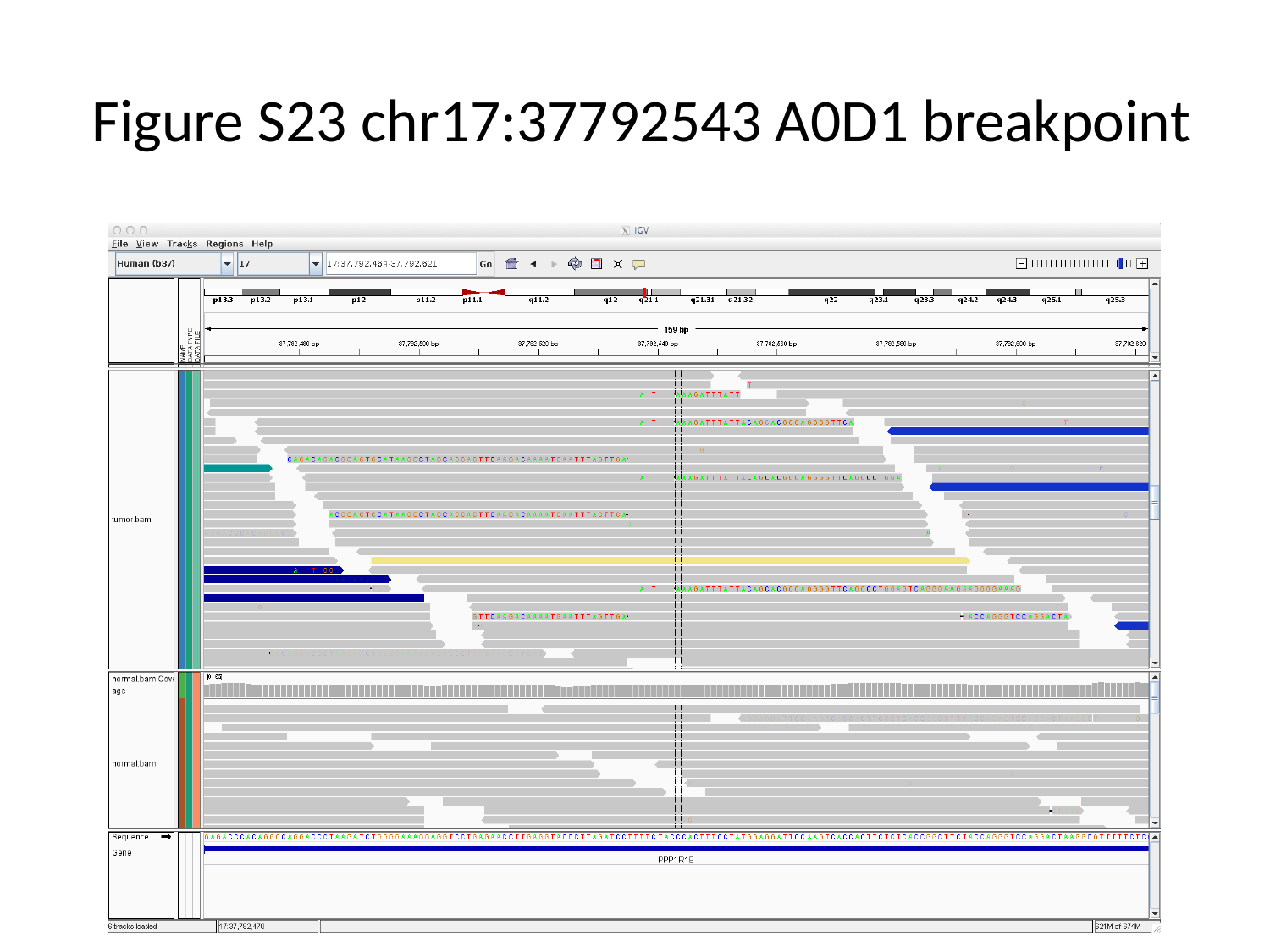

# Figure S23 chr17:37792543 A0D1 breakpoint

## Slide 24
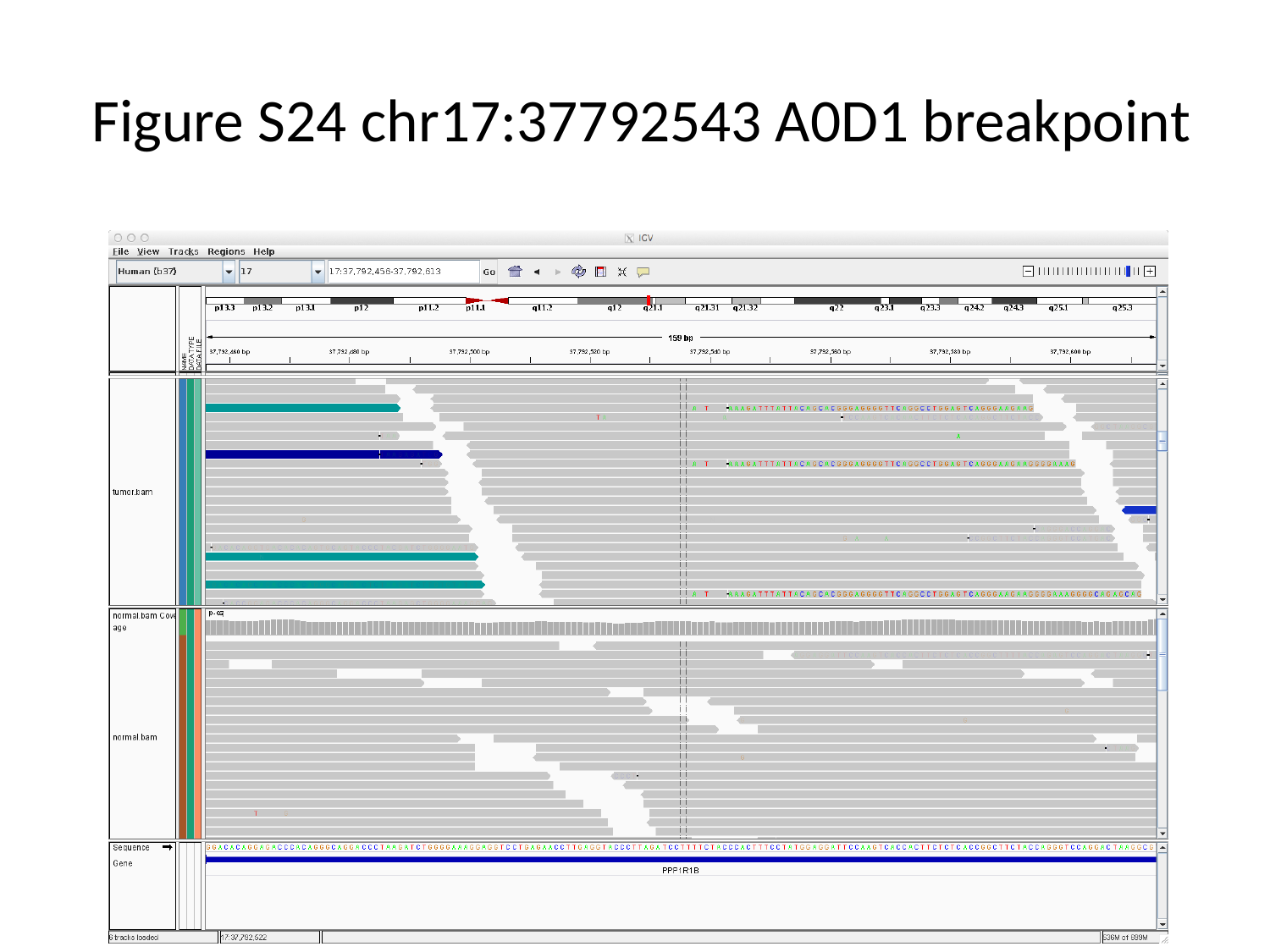

# Figure S24 chr17:37792543 A0D1 breakpoint

## Slide 25
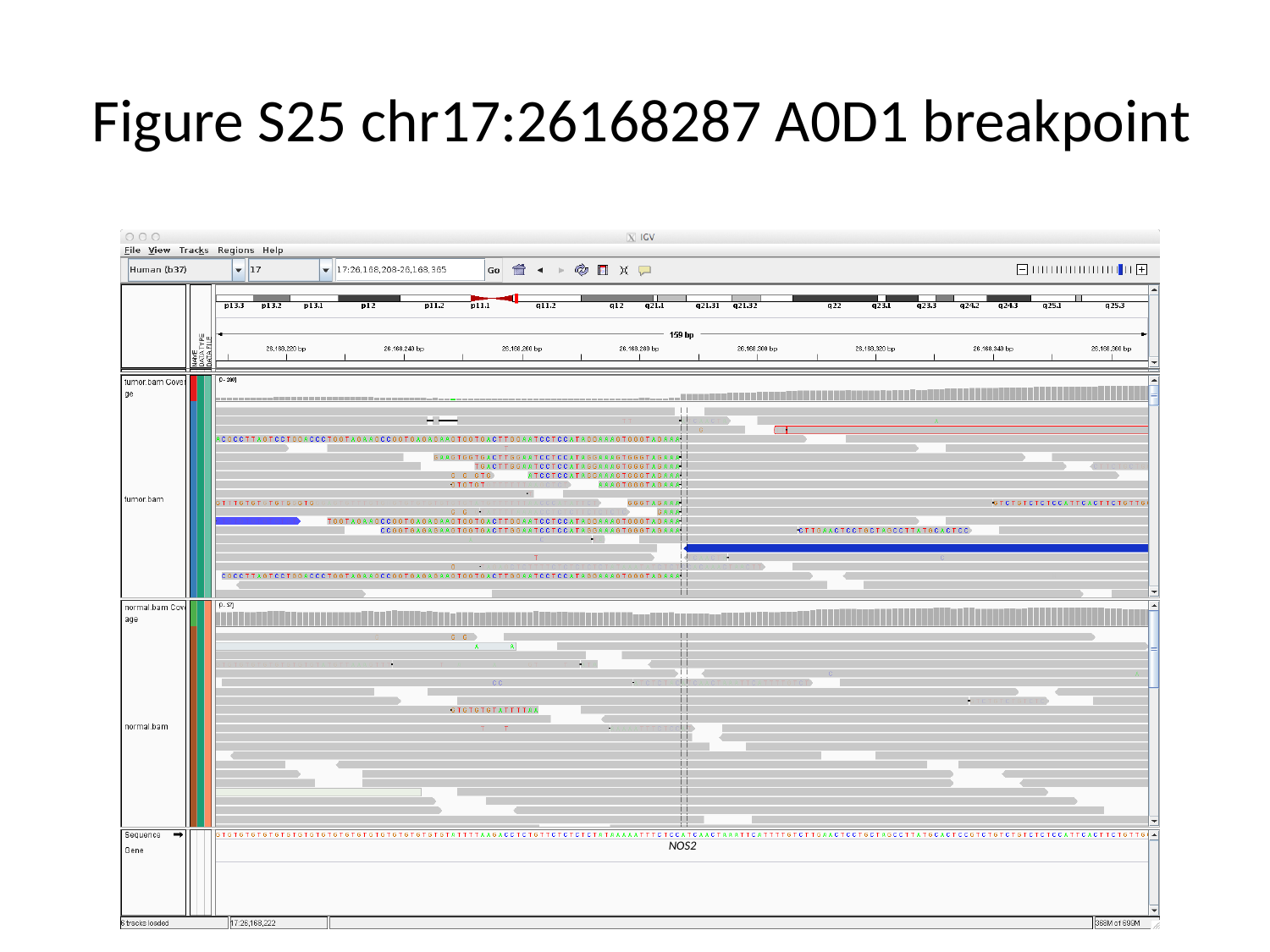

# Figure S25 chr17:26168287 A0D1 breakpoint
NOS2

## Slide 26
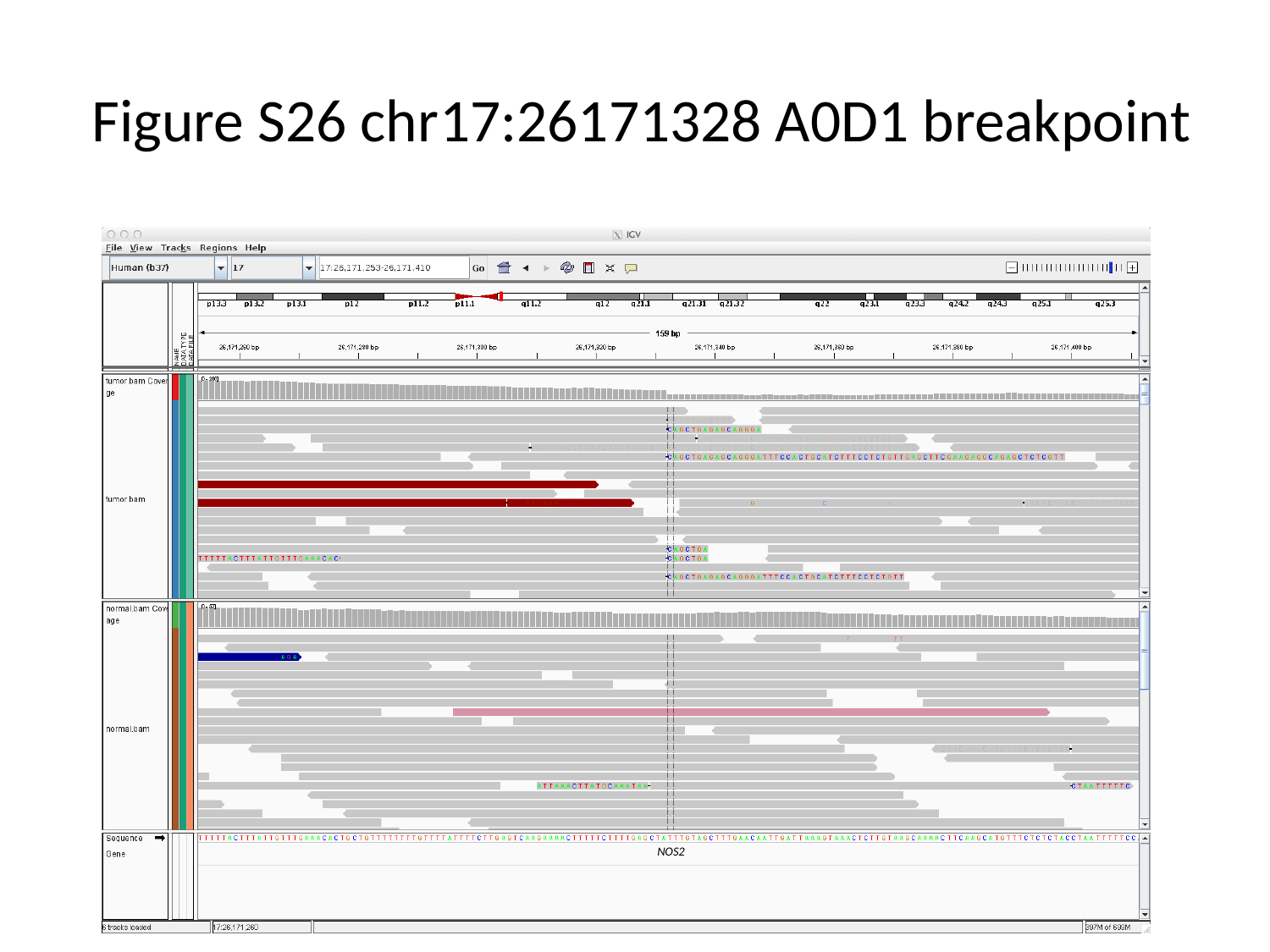

# Figure S26 chr17:26171328 A0D1 breakpoint
NOS2

## Slide 27
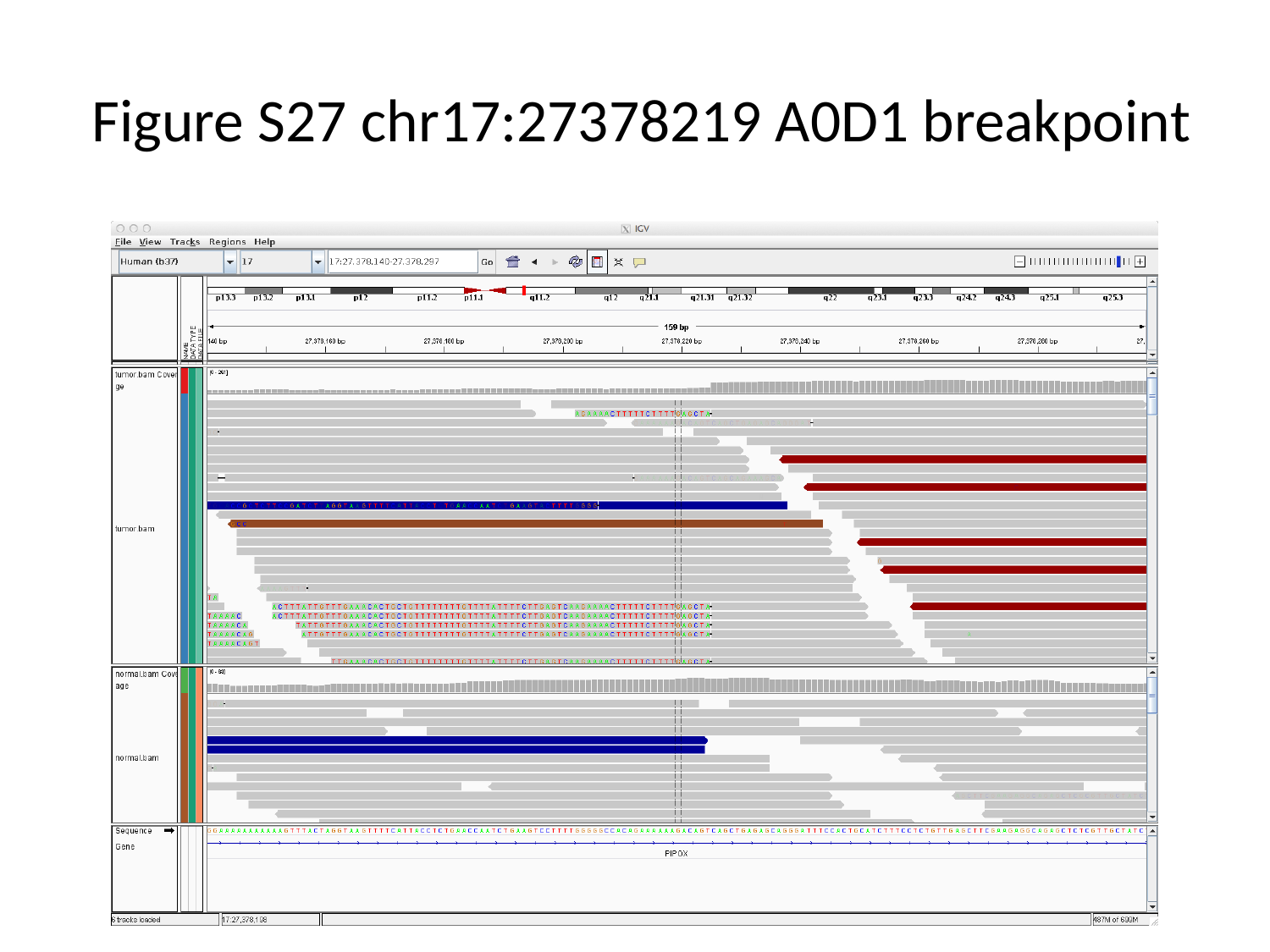

# Figure S27 chr17:27378219 A0D1 breakpoint

## Slide 28
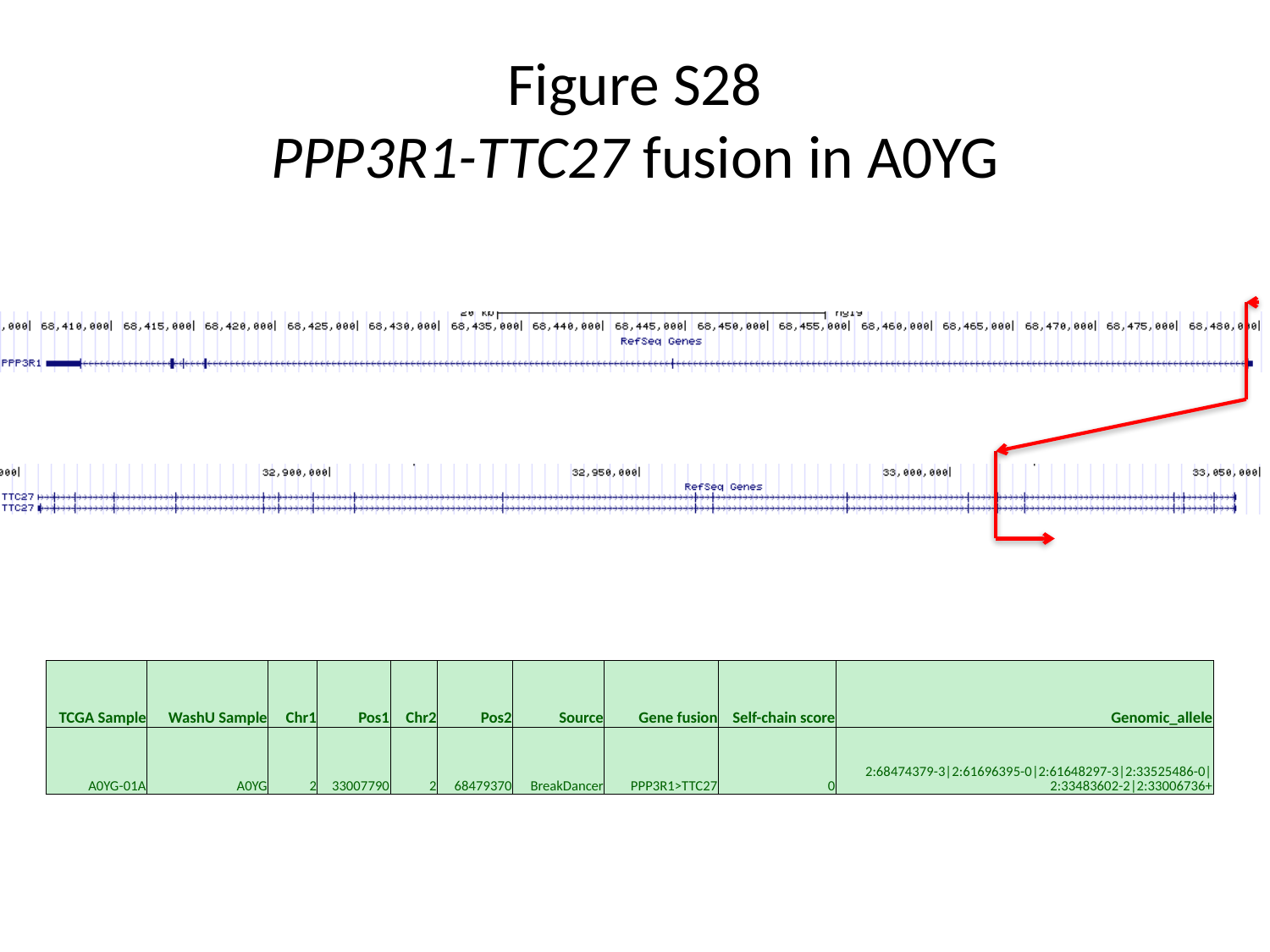

# Figure S28 PPP3R1-TTC27 fusion in A0YG
| TCGA Sample | WashU Sample | Chr1 | Pos1 | Chr2 | Pos2 | Source | Gene fusion | Self-chain score | Genomic\_allele |
| --- | --- | --- | --- | --- | --- | --- | --- | --- | --- |
| A0YG-01A | A0YG | 2 | 33007790 | 2 | 68479370 | BreakDancer | PPP3R1>TTC27 | 0 | 2:68474379-3|2:61696395-0|2:61648297-3|2:33525486-0|2:33483602-2|2:33006736+ |

## Slide 29
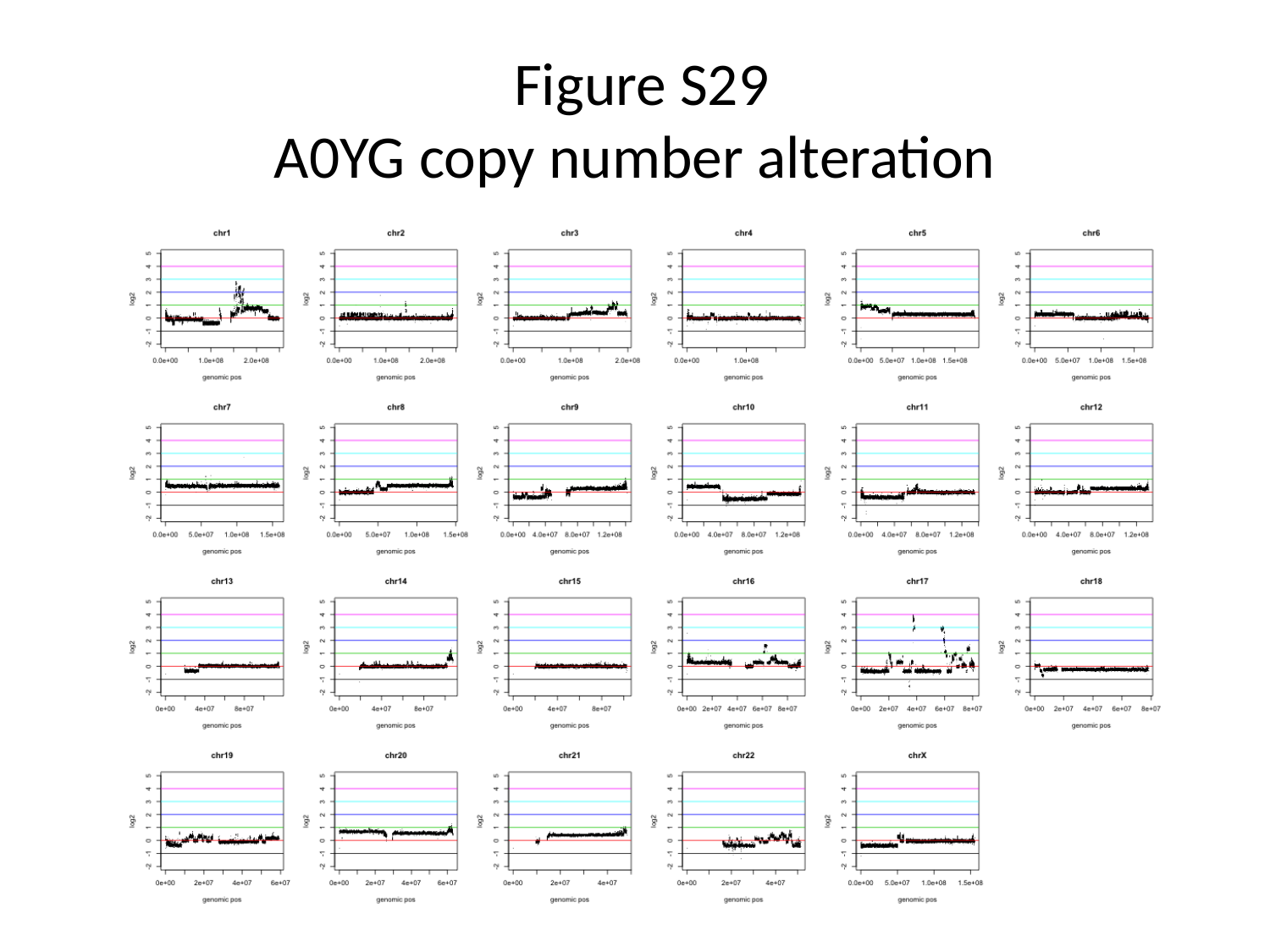

# Figure S29 A0YG copy number alteration

## Slide 30
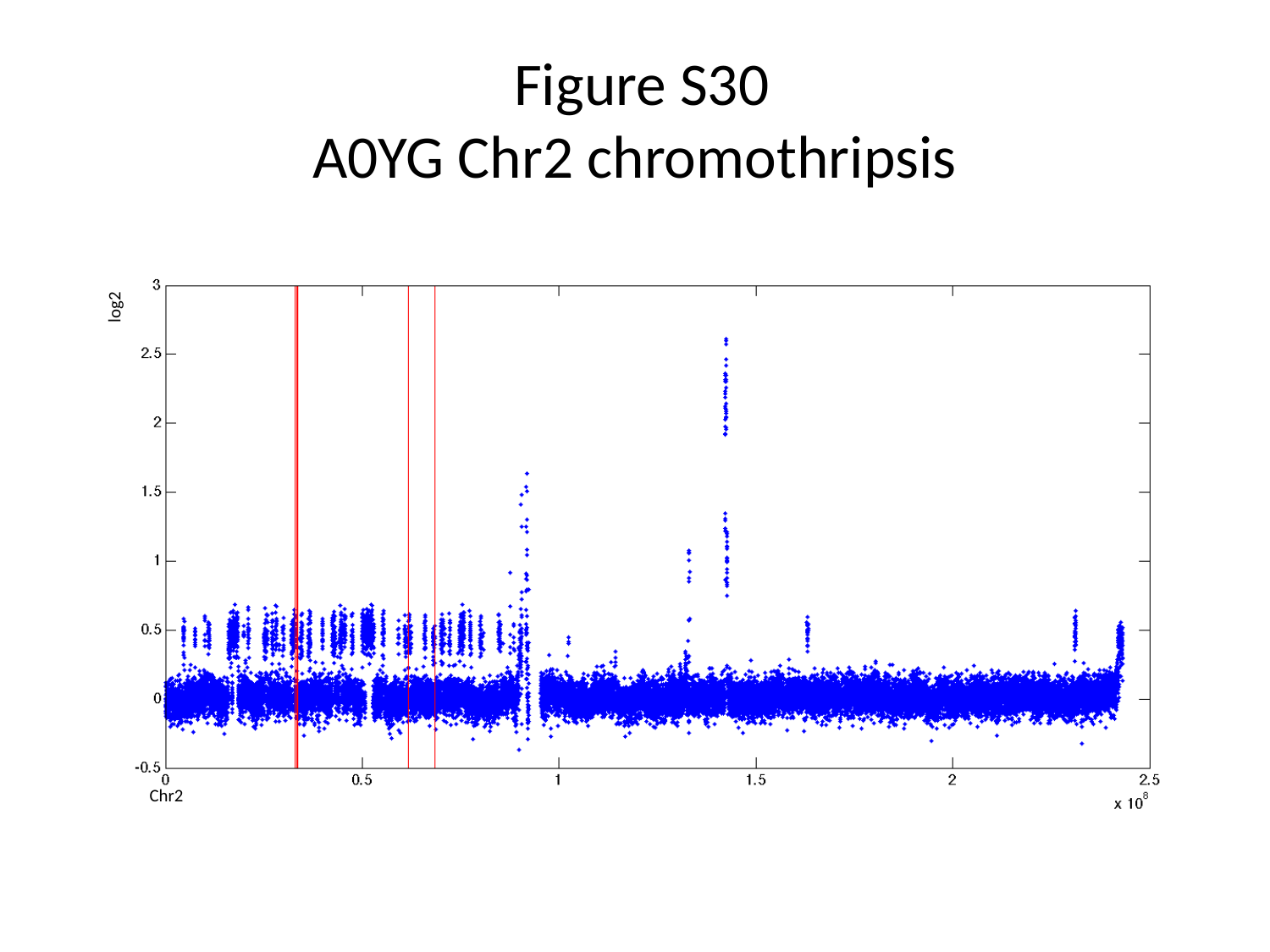

# Figure S30 A0YG Chr2 chromothripsis
log2
Chr2

## Slide 31
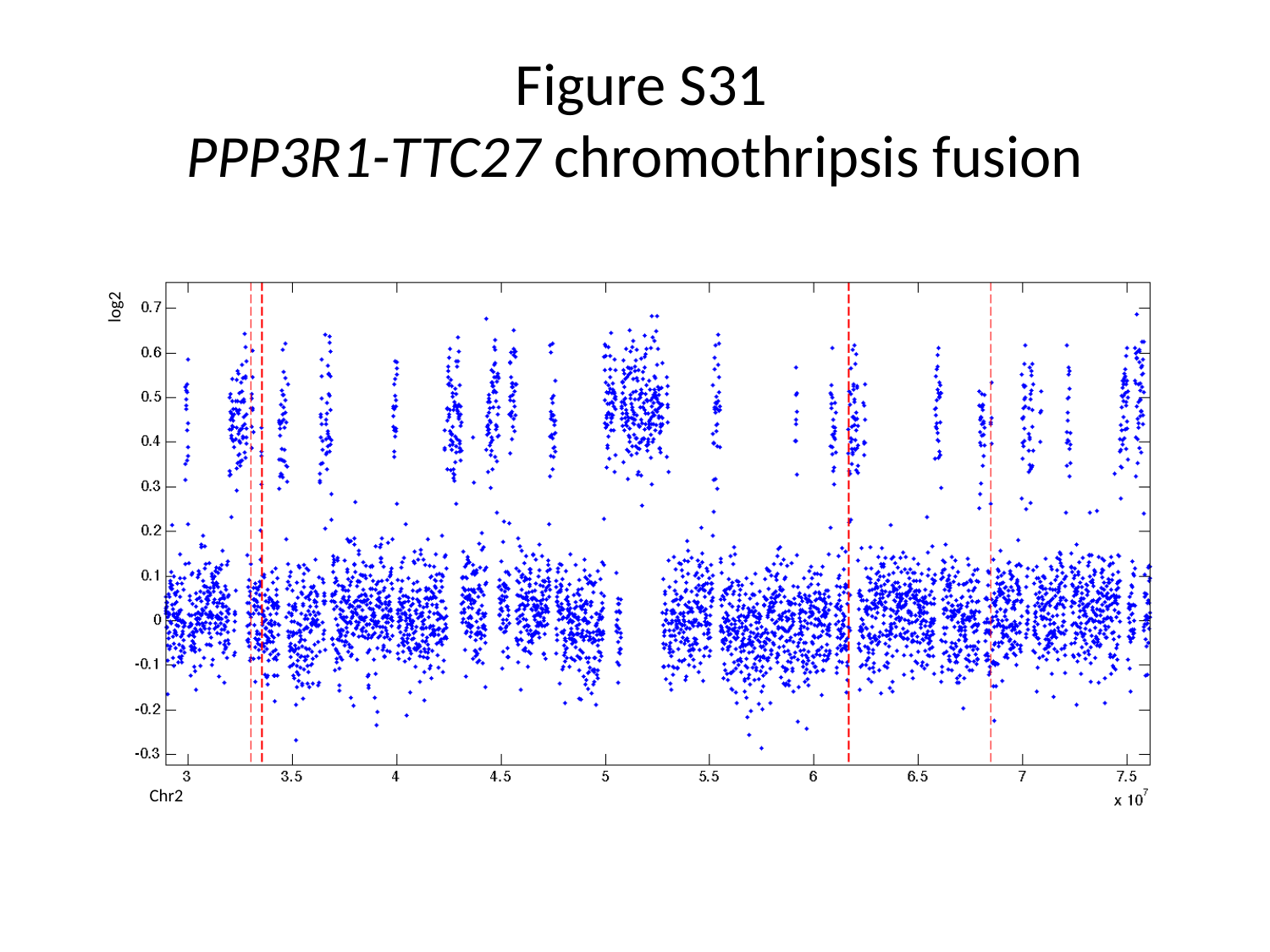

# Figure S31 PPP3R1-TTC27 chromothripsis fusion
log2
Chr2

## Slide 32
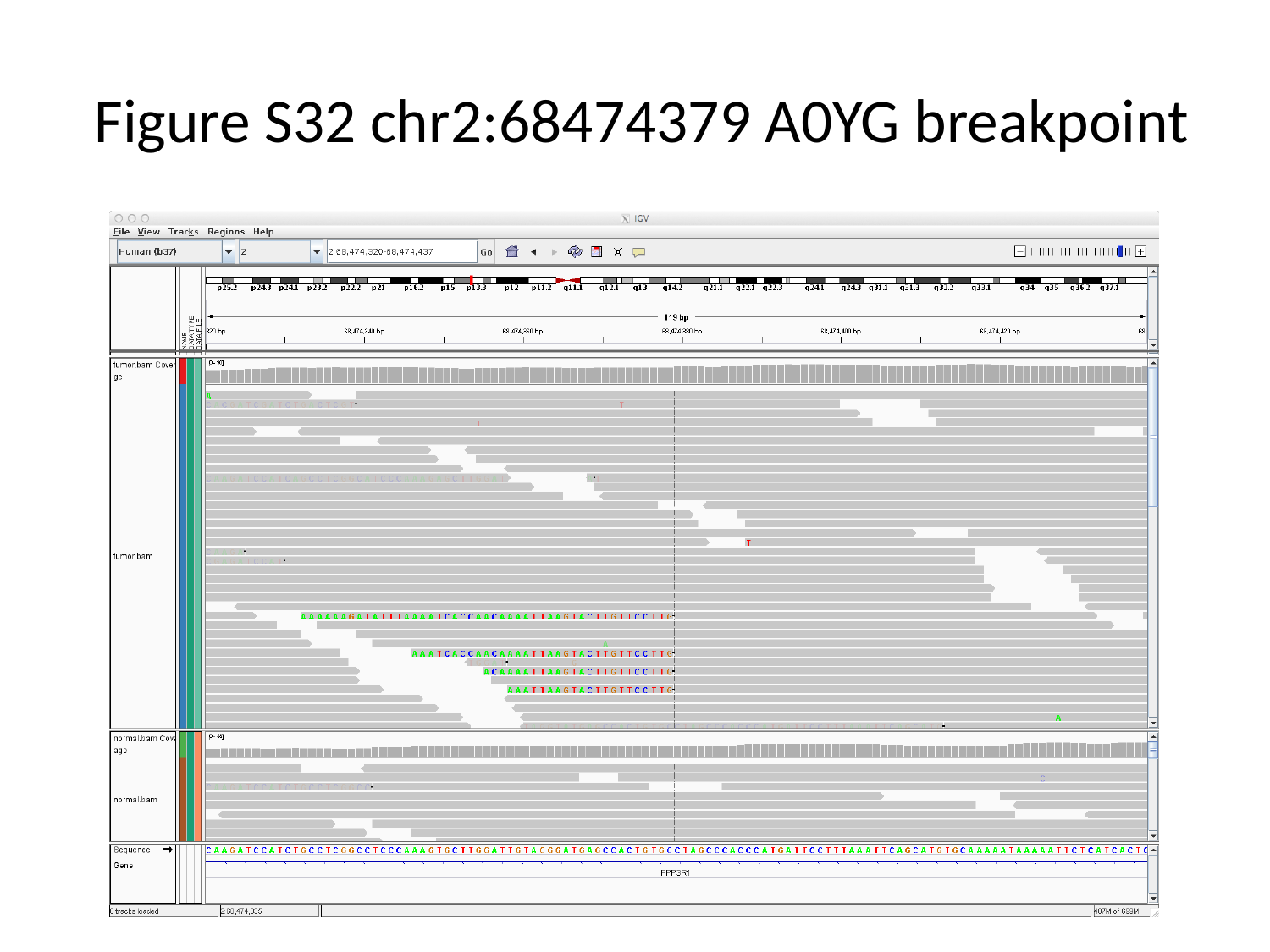

# Figure S32 chr2:68474379 A0YG breakpoint

## Slide 33
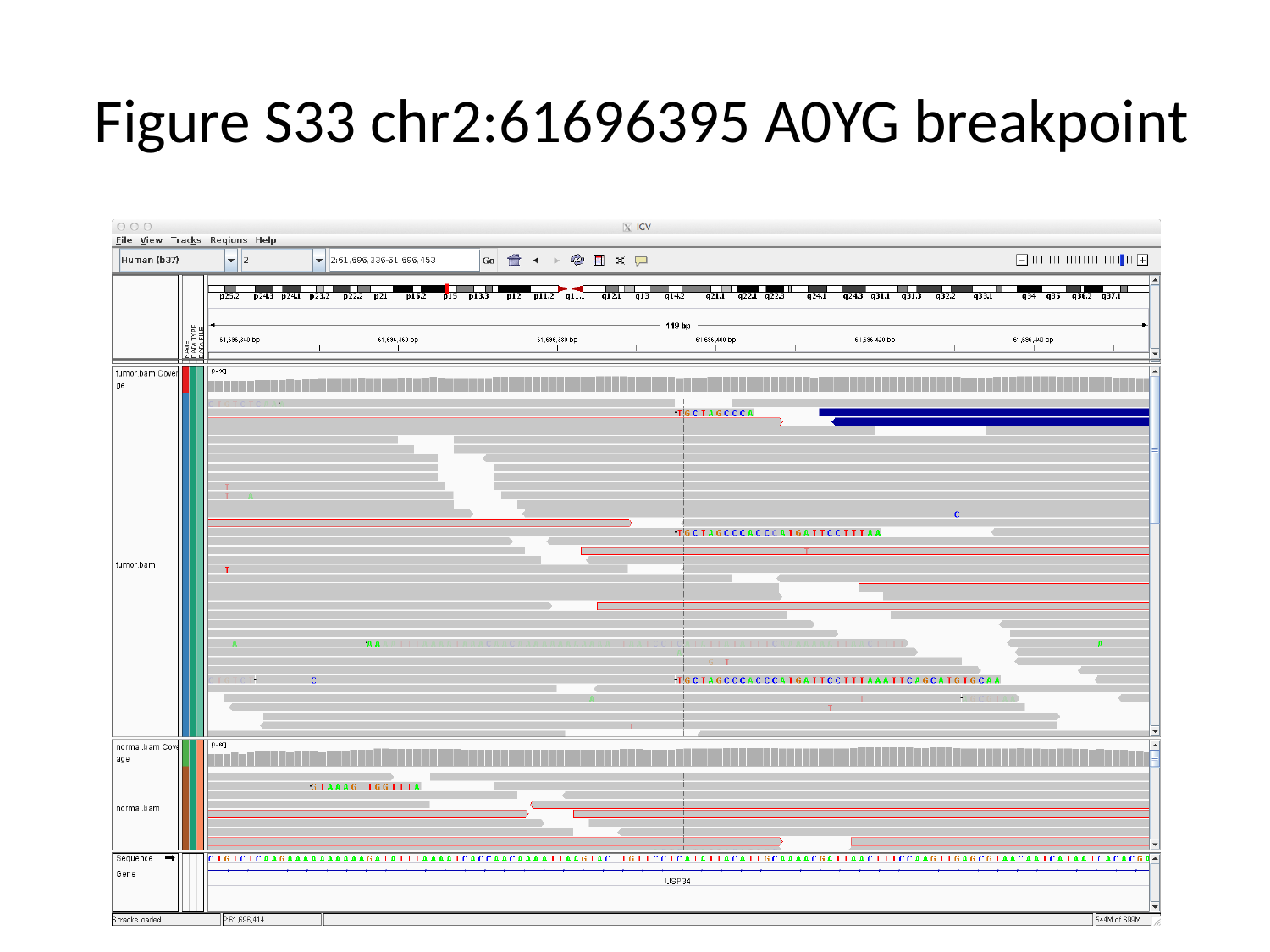

# Figure S33 chr2:61696395 A0YG breakpoint

## Slide 34
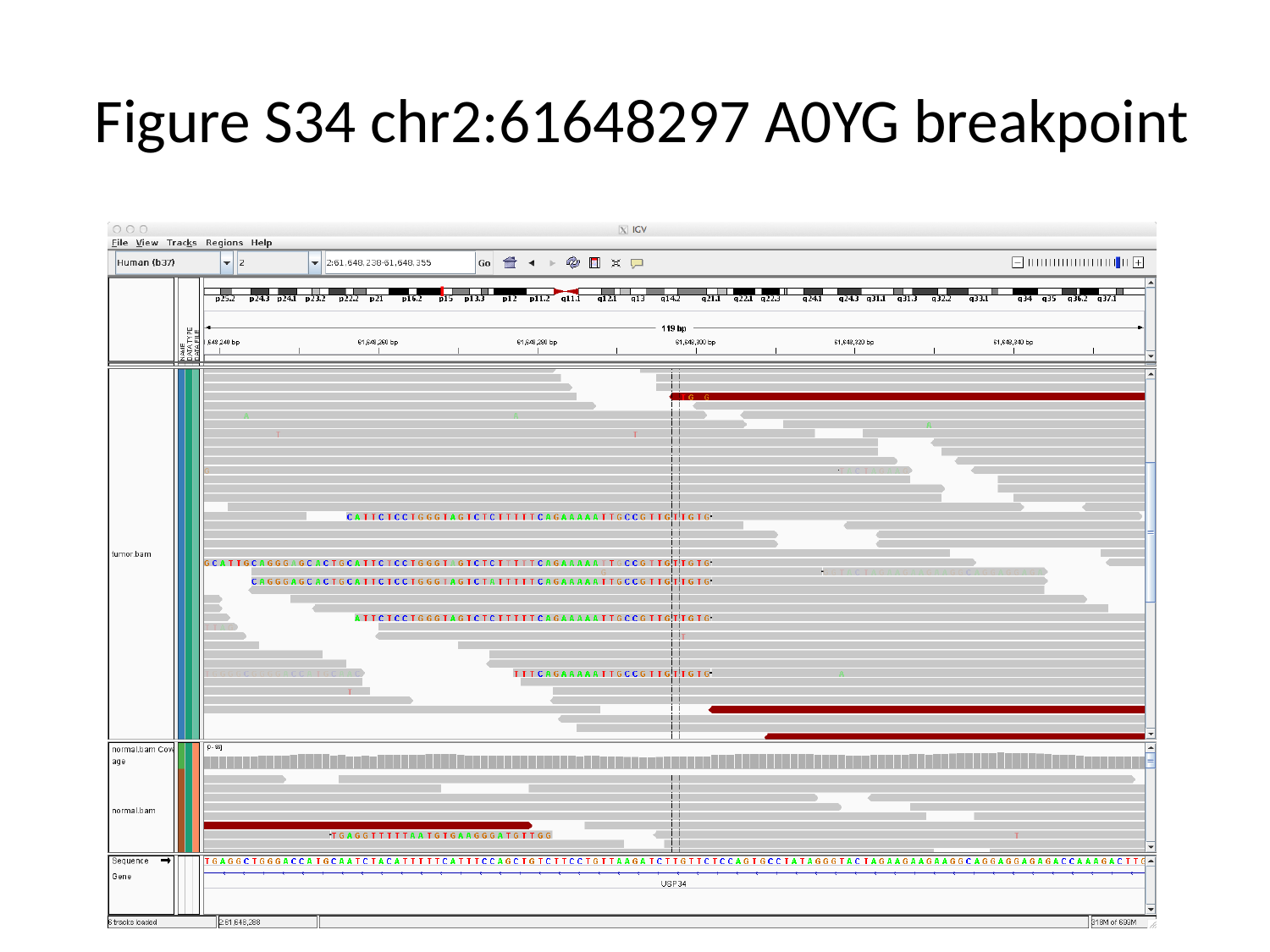

# Figure S34 chr2:61648297 A0YG breakpoint

## Slide 35
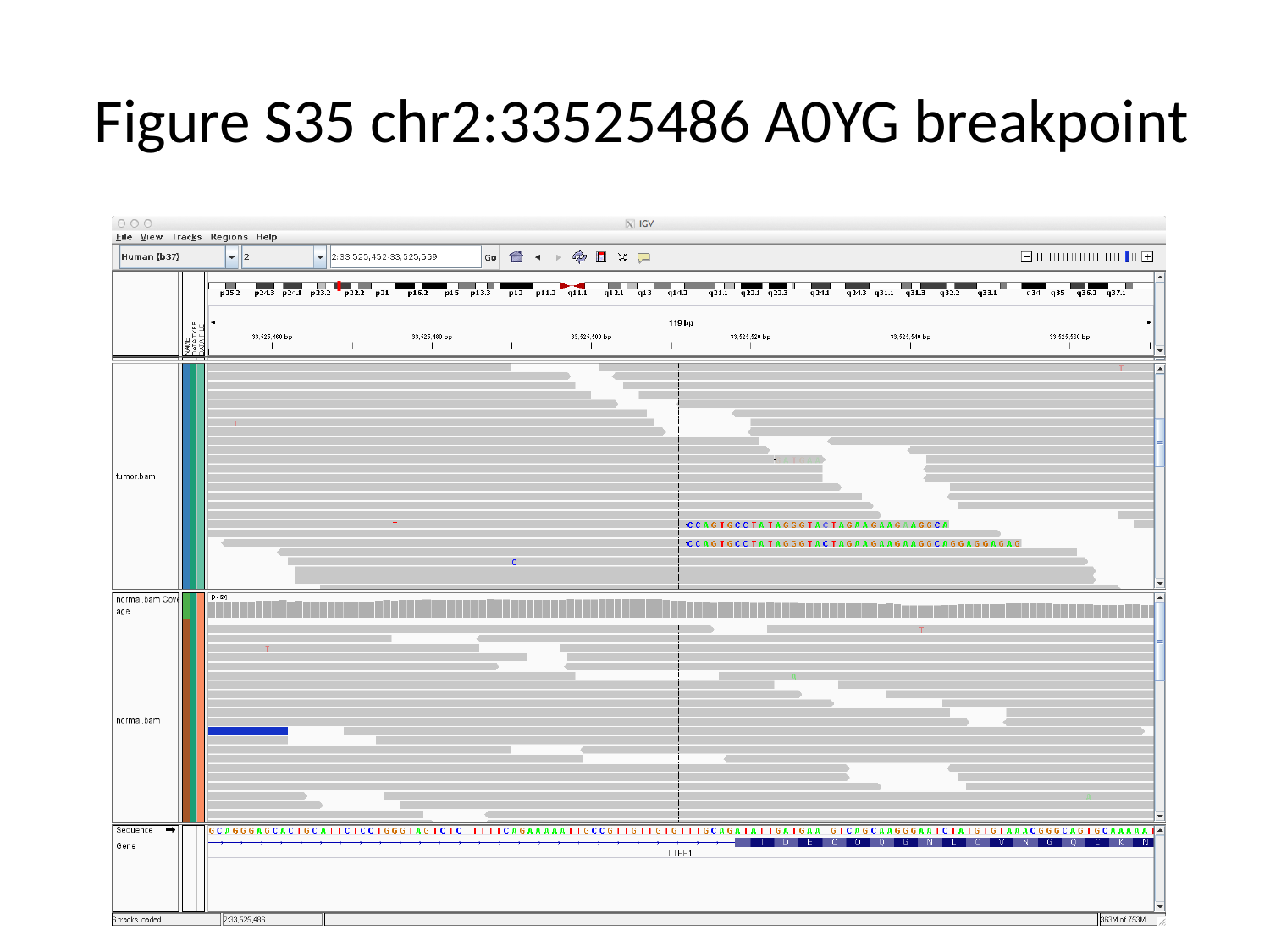

# Figure S35 chr2:33525486 A0YG breakpoint

## Slide 36
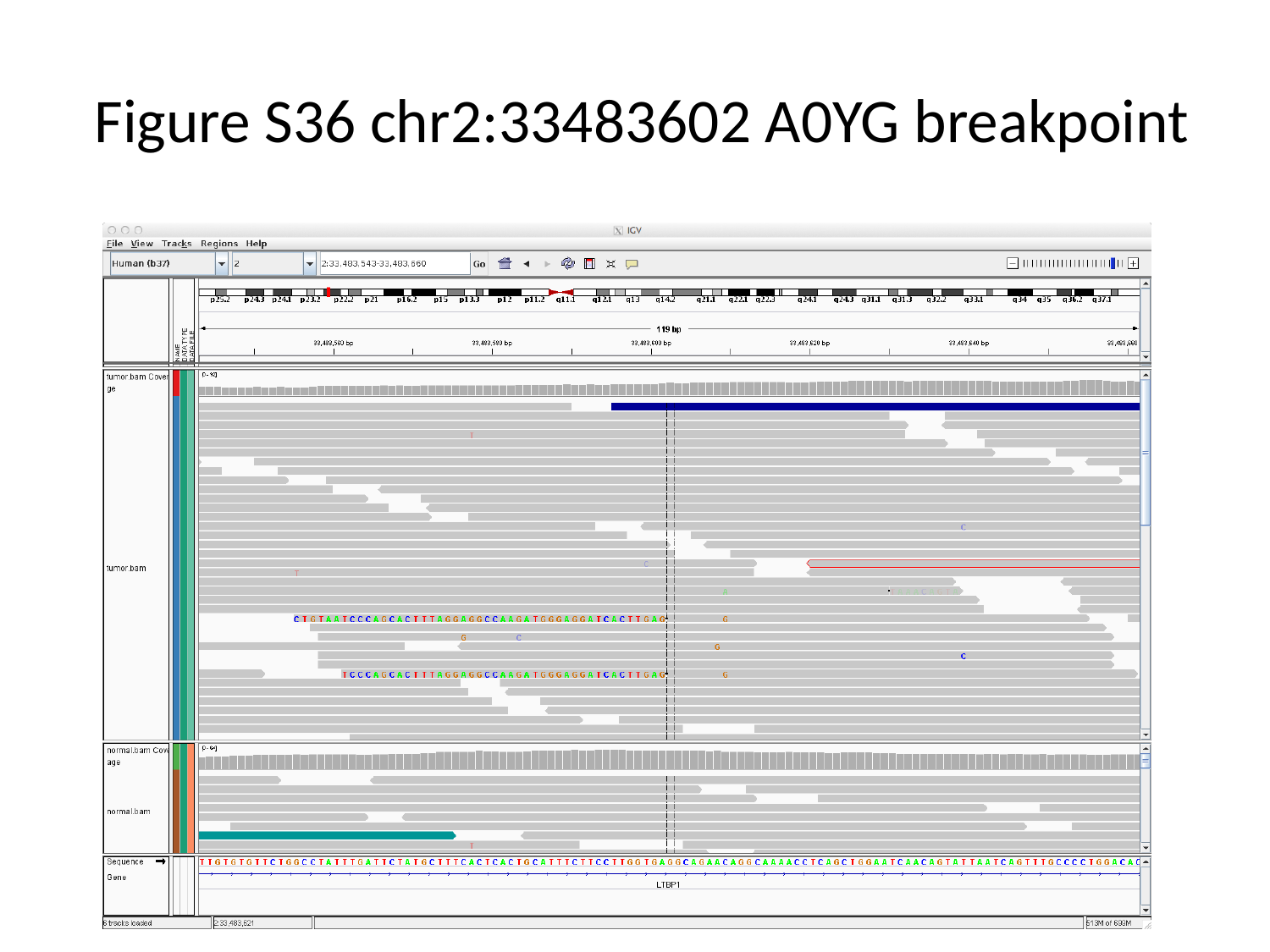

# Figure S36 chr2:33483602 A0YG breakpoint

## Slide 37
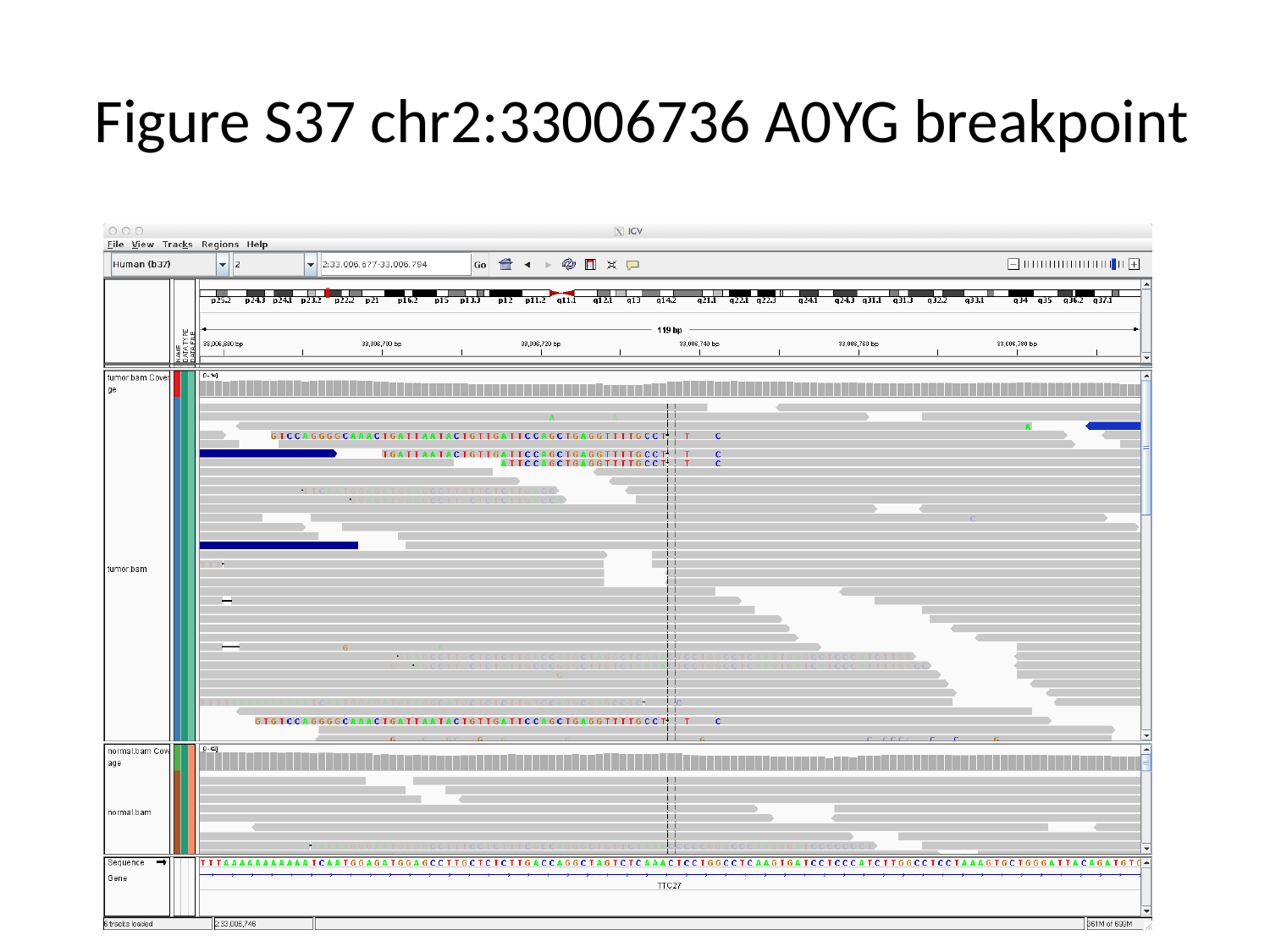

# Figure S37 chr2:33006736 A0YG breakpoint

## Slide 38
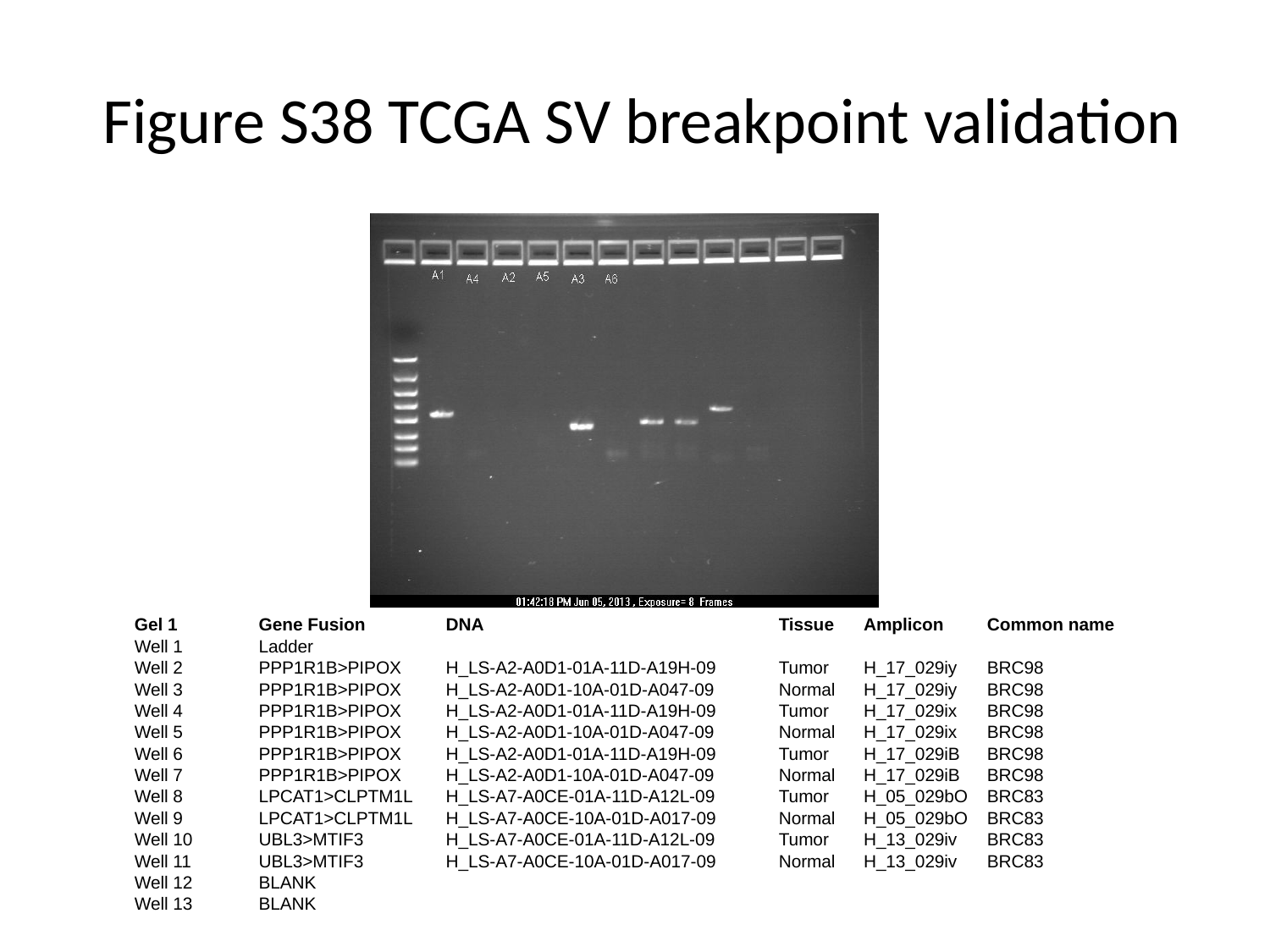

# Figure S38 TCGA SV breakpoint validation
| Gel 1 | Gene Fusion | DNA | Tissue | Amplicon | Common name |
| --- | --- | --- | --- | --- | --- |
| Well 1 | Ladder | | | | |
| Well 2 | PPP1R1B>PIPOX | H\_LS-A2-A0D1-01A-11D-A19H-09 | Tumor | H\_17\_029iy | BRC98 |
| Well 3 | PPP1R1B>PIPOX | H\_LS-A2-A0D1-10A-01D-A047-09 | Normal | H\_17\_029iy | BRC98 |
| Well 4 | PPP1R1B>PIPOX | H\_LS-A2-A0D1-01A-11D-A19H-09 | Tumor | H\_17\_029ix | BRC98 |
| Well 5 | PPP1R1B>PIPOX | H\_LS-A2-A0D1-10A-01D-A047-09 | Normal | H\_17\_029ix | BRC98 |
| Well 6 | PPP1R1B>PIPOX | H\_LS-A2-A0D1-01A-11D-A19H-09 | Tumor | H\_17\_029iB | BRC98 |
| Well 7 | PPP1R1B>PIPOX | H\_LS-A2-A0D1-10A-01D-A047-09 | Normal | H\_17\_029iB | BRC98 |
| Well 8 | LPCAT1>CLPTM1L | H\_LS-A7-A0CE-01A-11D-A12L-09 | Tumor | H\_05\_029bO | BRC83 |
| Well 9 | LPCAT1>CLPTM1L | H\_LS-A7-A0CE-10A-01D-A017-09 | Normal | H\_05\_029bO | BRC83 |
| Well 10 | UBL3>MTIF3 | H\_LS-A7-A0CE-01A-11D-A12L-09 | Tumor | H\_13\_029iv | BRC83 |
| Well 11 | UBL3>MTIF3 | H\_LS-A7-A0CE-10A-01D-A017-09 | Normal | H\_13\_029iv | BRC83 |
| Well 12 | BLANK | | | | |
| Well 13 | BLANK | | | | |

## Slide 39
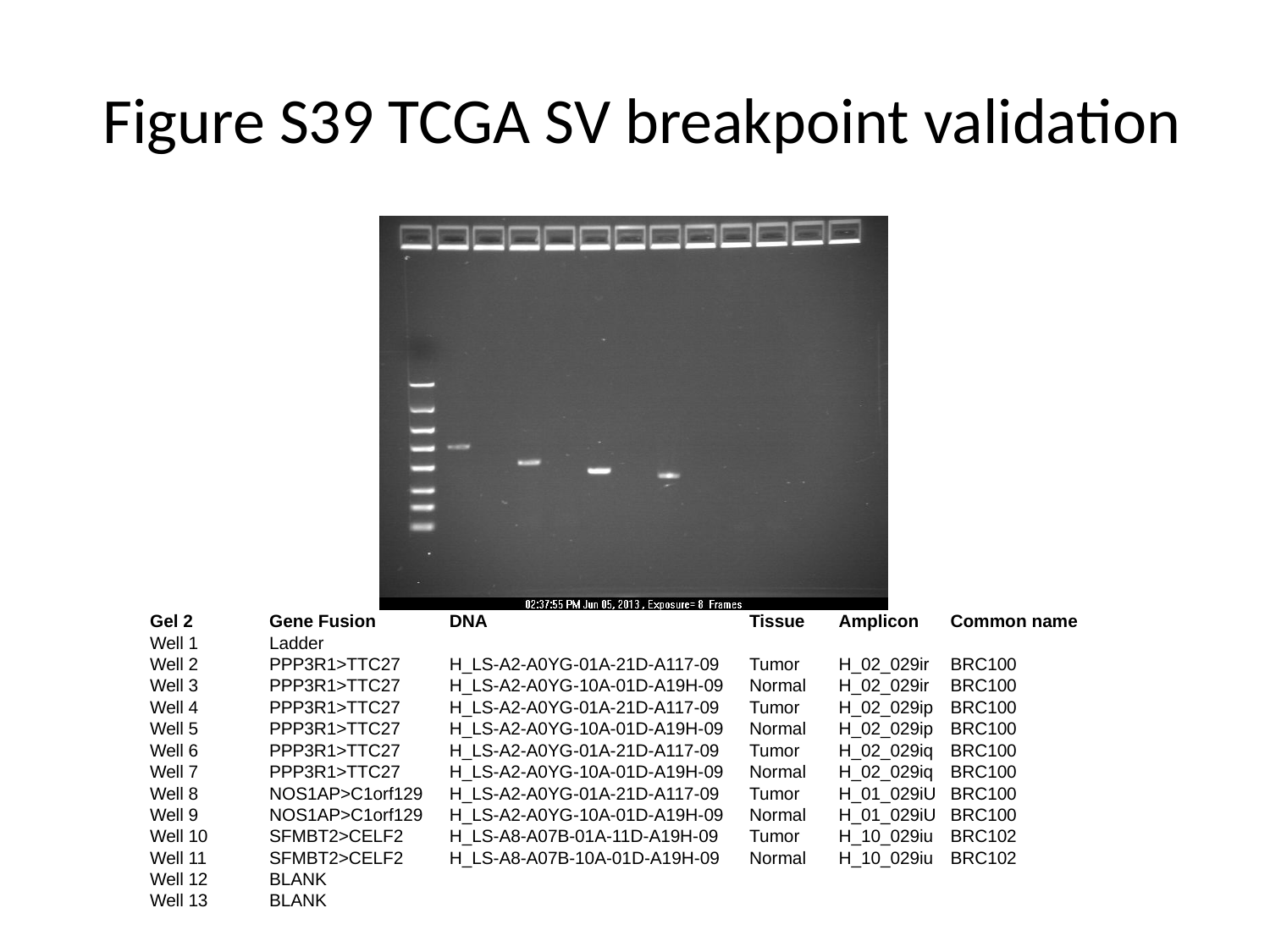

# Figure S39 TCGA SV breakpoint validation
| Gel 2 | Gene Fusion | DNA | Tissue | Amplicon | Common name |
| --- | --- | --- | --- | --- | --- |
| Well 1 | Ladder | | | | |
| Well 2 | PPP3R1>TTC27 | H\_LS-A2-A0YG-01A-21D-A117-09 | Tumor | H\_02\_029ir | BRC100 |
| Well 3 | PPP3R1>TTC27 | H\_LS-A2-A0YG-10A-01D-A19H-09 | Normal | H\_02\_029ir | BRC100 |
| Well 4 | PPP3R1>TTC27 | H\_LS-A2-A0YG-01A-21D-A117-09 | Tumor | H\_02\_029ip | BRC100 |
| Well 5 | PPP3R1>TTC27 | H\_LS-A2-A0YG-10A-01D-A19H-09 | Normal | H\_02\_029ip | BRC100 |
| Well 6 | PPP3R1>TTC27 | H\_LS-A2-A0YG-01A-21D-A117-09 | Tumor | H\_02\_029iq | BRC100 |
| Well 7 | PPP3R1>TTC27 | H\_LS-A2-A0YG-10A-01D-A19H-09 | Normal | H\_02\_029iq | BRC100 |
| Well 8 | NOS1AP>C1orf129 | H\_LS-A2-A0YG-01A-21D-A117-09 | Tumor | H\_01\_029iU | BRC100 |
| Well 9 | NOS1AP>C1orf129 | H\_LS-A2-A0YG-10A-01D-A19H-09 | Normal | H\_01\_029iU | BRC100 |
| Well 10 | SFMBT2>CELF2 | H\_LS-A8-A07B-01A-11D-A19H-09 | Tumor | H\_10\_029iu | BRC102 |
| Well 11 | SFMBT2>CELF2 | H\_LS-A8-A07B-10A-01D-A19H-09 | Normal | H\_10\_029iu | BRC102 |
| Well 12 | BLANK | | | | |
| Well 13 | BLANK | | | | |

## Slide 40
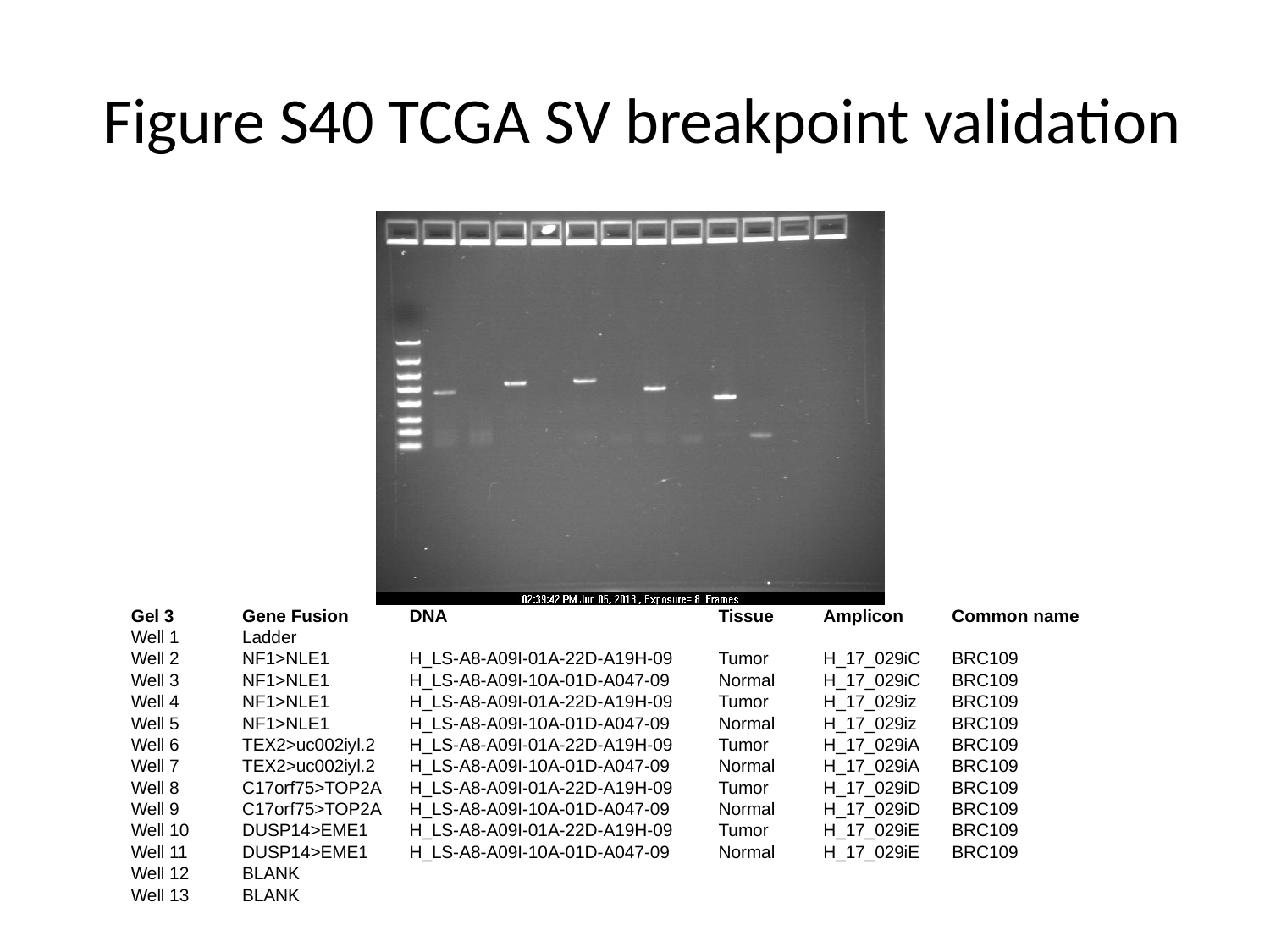

# Figure S40 TCGA SV breakpoint validation
| Gel 3 | Gene Fusion | DNA | Tissue | Amplicon | Common name |
| --- | --- | --- | --- | --- | --- |
| Well 1 | Ladder | | | | |
| Well 2 | NF1>NLE1 | H\_LS-A8-A09I-01A-22D-A19H-09 | Tumor | H\_17\_029iC | BRC109 |
| Well 3 | NF1>NLE1 | H\_LS-A8-A09I-10A-01D-A047-09 | Normal | H\_17\_029iC | BRC109 |
| Well 4 | NF1>NLE1 | H\_LS-A8-A09I-01A-22D-A19H-09 | Tumor | H\_17\_029iz | BRC109 |
| Well 5 | NF1>NLE1 | H\_LS-A8-A09I-10A-01D-A047-09 | Normal | H\_17\_029iz | BRC109 |
| Well 6 | TEX2>uc002iyl.2 | H\_LS-A8-A09I-01A-22D-A19H-09 | Tumor | H\_17\_029iA | BRC109 |
| Well 7 | TEX2>uc002iyl.2 | H\_LS-A8-A09I-10A-01D-A047-09 | Normal | H\_17\_029iA | BRC109 |
| Well 8 | C17orf75>TOP2A | H\_LS-A8-A09I-01A-22D-A19H-09 | Tumor | H\_17\_029iD | BRC109 |
| Well 9 | C17orf75>TOP2A | H\_LS-A8-A09I-10A-01D-A047-09 | Normal | H\_17\_029iD | BRC109 |
| Well 10 | DUSP14>EME1 | H\_LS-A8-A09I-01A-22D-A19H-09 | Tumor | H\_17\_029iE | BRC109 |
| Well 11 | DUSP14>EME1 | H\_LS-A8-A09I-10A-01D-A047-09 | Normal | H\_17\_029iE | BRC109 |
| Well 12 | BLANK | | | | |
| Well 13 | BLANK | | | | |

## Slide 41
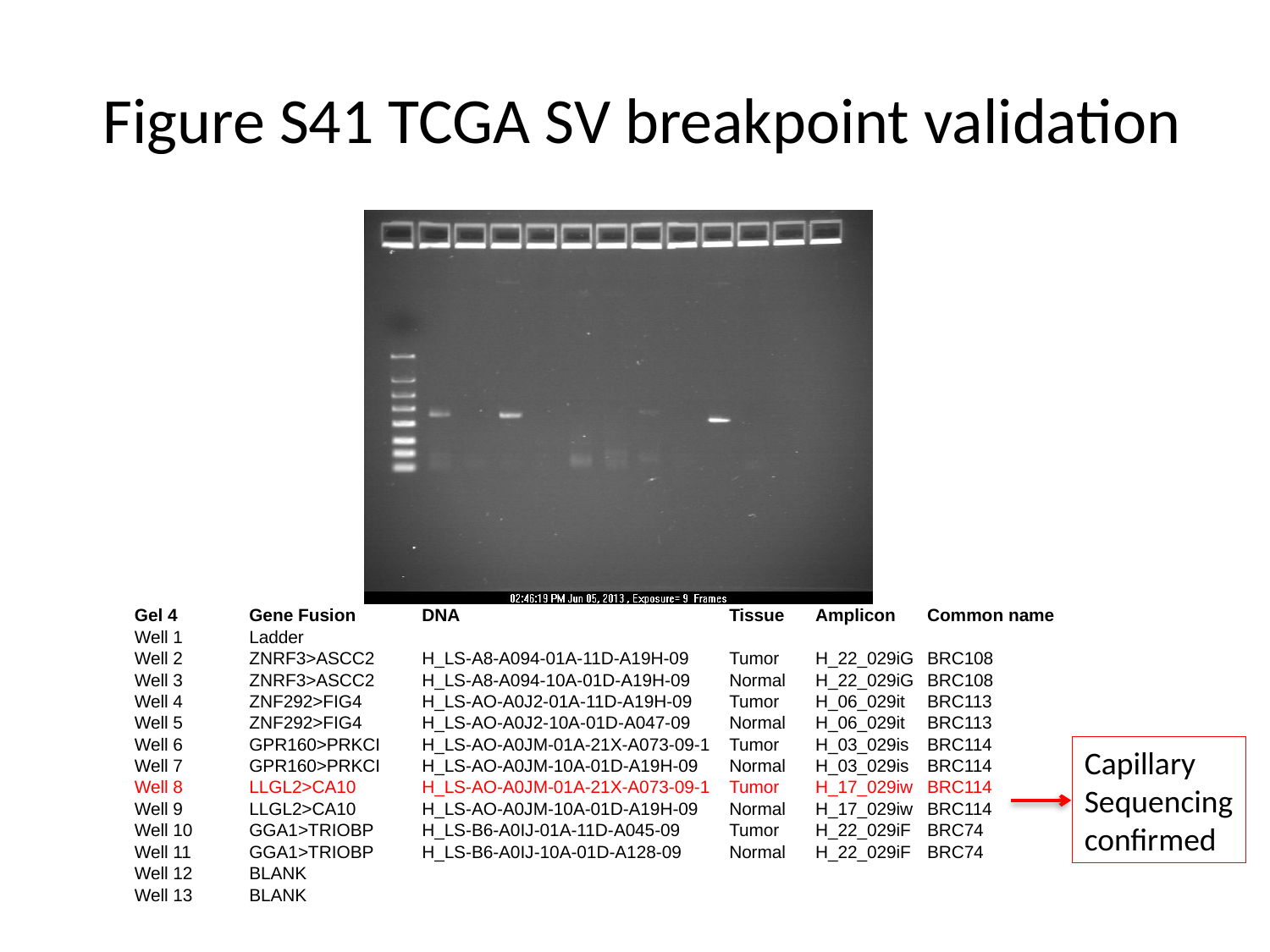

# Figure S41 TCGA SV breakpoint validation
| Gel 4 | Gene Fusion | DNA | Tissue | Amplicon | Common name |
| --- | --- | --- | --- | --- | --- |
| Well 1 | Ladder | | | | |
| Well 2 | ZNRF3>ASCC2 | H\_LS-A8-A094-01A-11D-A19H-09 | Tumor | H\_22\_029iG | BRC108 |
| Well 3 | ZNRF3>ASCC2 | H\_LS-A8-A094-10A-01D-A19H-09 | Normal | H\_22\_029iG | BRC108 |
| Well 4 | ZNF292>FIG4 | H\_LS-AO-A0J2-01A-11D-A19H-09 | Tumor | H\_06\_029it | BRC113 |
| Well 5 | ZNF292>FIG4 | H\_LS-AO-A0J2-10A-01D-A047-09 | Normal | H\_06\_029it | BRC113 |
| Well 6 | GPR160>PRKCI | H\_LS-AO-A0JM-01A-21X-A073-09-1 | Tumor | H\_03\_029is | BRC114 |
| Well 7 | GPR160>PRKCI | H\_LS-AO-A0JM-10A-01D-A19H-09 | Normal | H\_03\_029is | BRC114 |
| Well 8 | LLGL2>CA10 | H\_LS-AO-A0JM-01A-21X-A073-09-1 | Tumor | H\_17\_029iw | BRC114 |
| Well 9 | LLGL2>CA10 | H\_LS-AO-A0JM-10A-01D-A19H-09 | Normal | H\_17\_029iw | BRC114 |
| Well 10 | GGA1>TRIOBP | H\_LS-B6-A0IJ-01A-11D-A045-09 | Tumor | H\_22\_029iF | BRC74 |
| Well 11 | GGA1>TRIOBP | H\_LS-B6-A0IJ-10A-01D-A128-09 | Normal | H\_22\_029iF | BRC74 |
| Well 12 | BLANK | | | | |
| Well 13 | BLANK | | | | |
Capillary
Sequencing
confirmed

## Slide 42
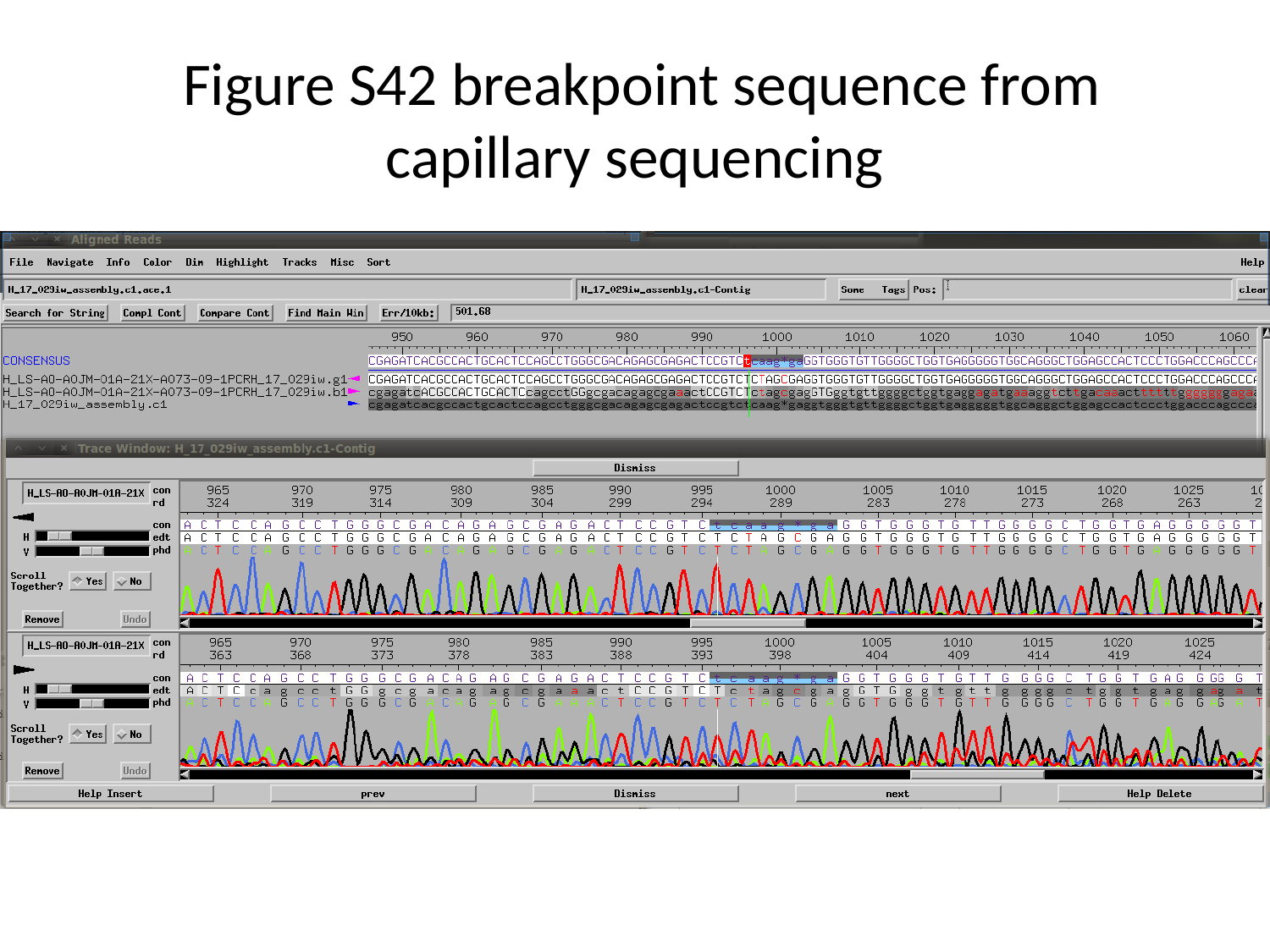

# Figure S42 breakpoint sequence from capillary sequencing
